# Supplementary material for: Economic valuation of temperature-related mortality attributed to urban heat islands in European cities
Source: Nat Commun. 2023 Nov 17;14:7438. doi: 10.1038/s41467-023-43135-z (PMC10656443; doi:10.1038/s41467-023-43135-z)
Supplement: Supplementary file 1 — Supplementary Information [file 41467_2023_43135_MOESM1_ESM.pdf]

Supplementary material for: Economic valuation of temperature-related mortality attributed to urban heat islands in European cities

Wan Ting Katty Huang[1,2], Pierre Masselot[3], Elie Bou-Zeid[4], Simone Fatichi[5], Athanasios Paschalis[6], Ting Sun[7], Antonio Gasparrini[3,8,9], and Gabriele Manoli[1,10]*

[1] Department of Civil, Environmental and Geomatic Engineering, University College London, London, UK
[2] Met Office, Exeter, UK
[3] Department of Public Health, Environments and Society, London School of Hygiene & Tropical Medicine, London, UK
[4] Department of Civil and Environmental Engineering, Princeton University, Princeton, USA
[5] Department of Civil & Environmental Engineering, National University of Singapore, Singapore
[6] Department of Civil & Environmental Engineering, Imperial College London, UK
[7] Institute for Risk and Disaster Reduction, University College London, London, UK
[8] Centre for Statistical Methodology, London School of Hygiene & Tropical Medicine, London, UK
[9] Centre on Climate Change and Planetary Health, London School of Hygiene & Tropical Medicine, London, UK
[10] Laboratory of Urban and Environmental Systems, School of Architecture, Civil and Environmental Engineering, Ecole Polytechnique Fédérale de Lausanne (EPFL), Lausanne, Switzerland

* gabriele.manoli@epfl.ch

1 Population density and land imperviousness

UHI is driven by both the properties of the built environment as well as additional heat released by human activities. Consequently, increased UHI impact on mortality risk can also be observed with increasing population density within each city (Fig. S1). Given that densely populated areas also tend to be highly built up, correlation can be noted between population density and land imperviousness (mean Spearman correlation of 0.46 across 84 cities, each statistically significant with p-value less than 0.005). Exceptions include business and industrial districts without accompanying residential housing, as well as large car parks and airports. For instance, few people live in the highly built-up London city centre (despite high daytime working populations [1], which is not considered here given that population density is defined by residential address; Fig. S2e). Conversely, strategic urban greening can also lower the land imperviousness in highly populated areas [e.g. 2].

Elevated mortality risk can be noted in areas of either high population density or high land imperviousness, but risk is particularly pronounced in areas that are both highly populated and highly built-up (Fig. S2a,d,g). The relationship between population density and UHI is especially relevant when considering the impact on mortality risk, as more of the population live in areas with greater UHI. This bias in population exposure within the city, which is not considered in the analyses in the main text, leads to a 6.4% median (interquartile range, IQR: 2.9 to 9.4%) urban mortality risk enhancement during heat days and a 1.3% reduction (IQR: -2.3 to -0.7%) during cold days (Fig. S3). For the outlier cases of Brussels, Dublin, Paris, and Lyon, the urban mortality risk during heat days can be more than 25% greater when spatial correlation between where people live and the intensity of UHI is considered.

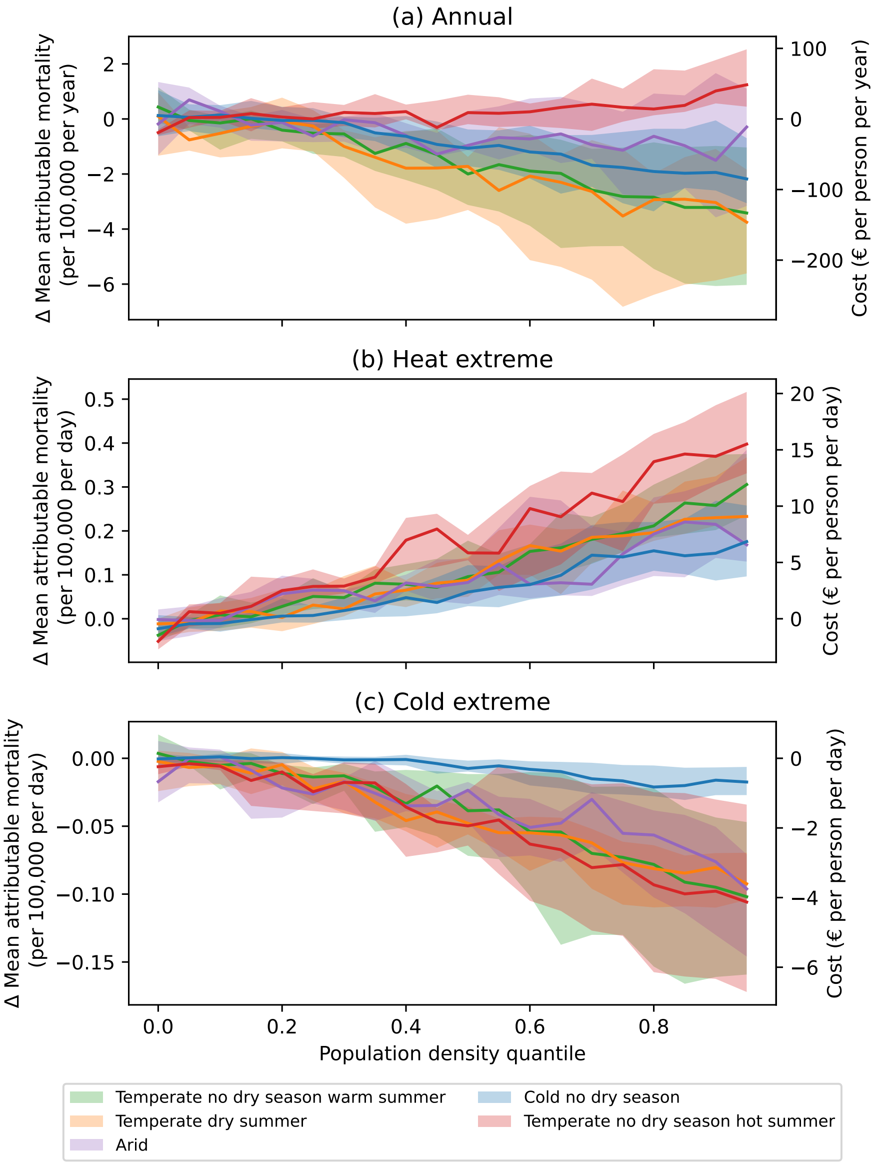

Figure S1: Same as Fig. 2 in the main text but with population density quantiles on the x-axis.

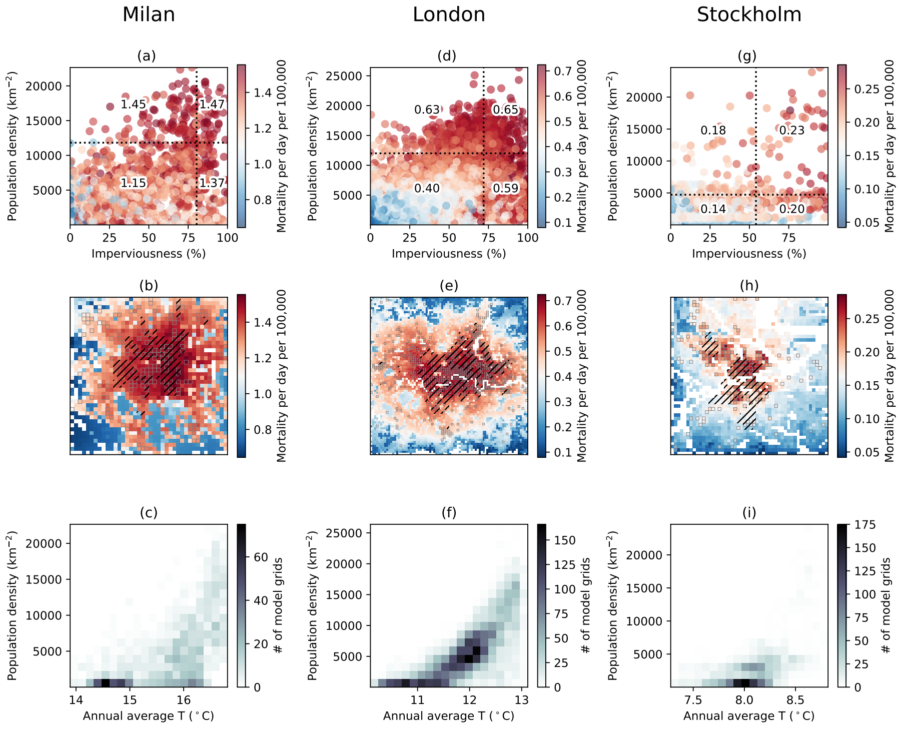

Figure S2: Relationship between land imperviousness, population density, and mortality risk in (a-c) Milan, (d-f) London, and (g-i) Stockholm. In panels (a,d,g), mortality risk during heat extreme days are shown on the population density-imperviousness phase space. Dotted vertical/horizontal lines indicate the 90th percentile imperviousness/population density. Numbers indicate the average mortality risk in each quardrants of the phase space as divided by the 90th percentile lines. Panels (b,e,h) show the geospatial perspective with model grids of imperviousness above the 90th percentile outlined in boxes and areas of population density greater than the 90th percentile indicated by hatching. Panels (c,f,i) show the relationship between population density and annual average temperature.

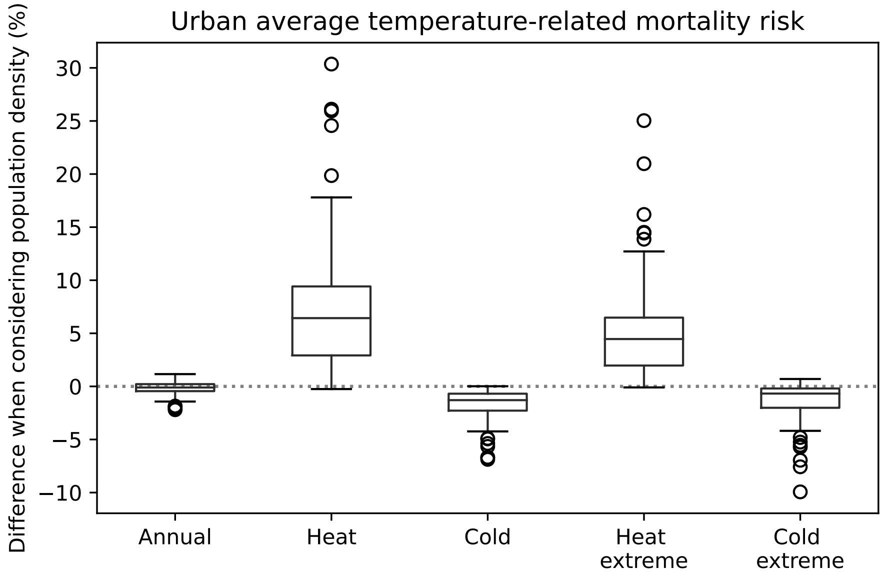

Figure S3: Difference in the urban mortality risk estimation for each city when the bias in population distribution within the urban fabric (more people living in higher UHI areas) is considered. Comparison is made between the estimation of risk per capita averaged across the urban domain and the average weighted by the population density within each model grid.

2 Population age structure

Unless otherwise specified, the current study includes consideration for differences in population age structure between cities. To exclude the role of population age in modulating mortality risk and for ease of comparison with other studies, the 2013 European standard population age structure [3] may be used for all cities. While the city populations examined in this study are largely in line with the standard population, variability exist between cities, and overall, slightly more cities have a younger rather than older population compared to the European standard (Fig. S4a). As the standard population is representative of all people regardless of their urban/rural residence, this may be indicative of populations in cities being younger than those in rural regions. Geographically, cities in western Europe have a greater likelihood to be older than the standard population (Fig. S4b), likely also reflecting the population age in these countries overall. Compared to the mortality risk estimated for the standard population, estimates for local populations are on median 9% lower (Fig. S4c), with geographical patterns (Fig. S4d) largely reflecting that of the age structure differences (Fig. S4b).

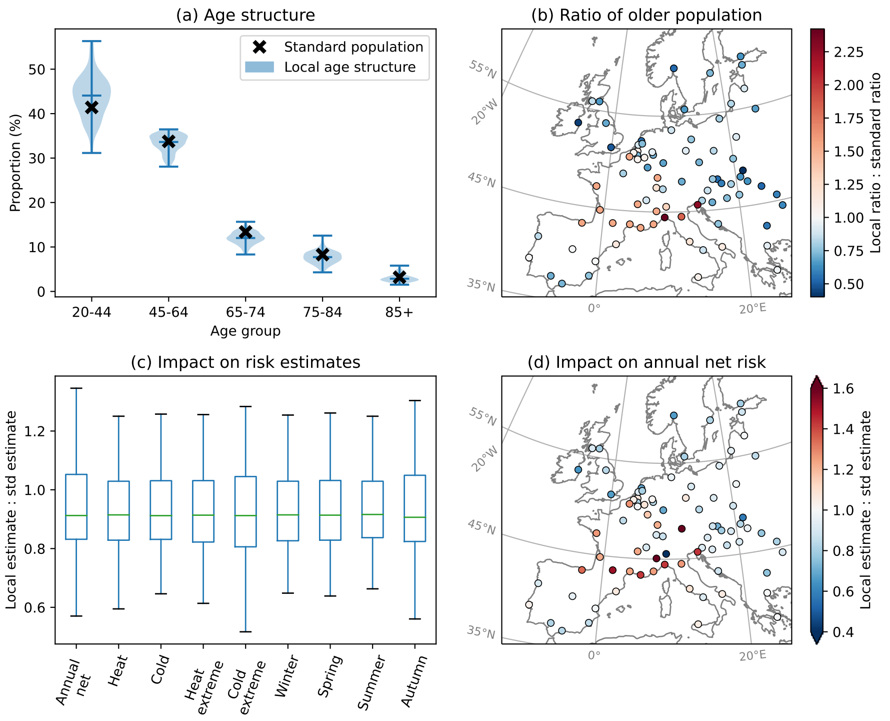

Figure S4: Comparison of the local population age structure to the 2013 European standard population, and the impact of assumed population age structure on the mortality risk estimation. Panel (a) shows the comparison of age structures between the standard population (X's) and local populations (violin plots, with bars indicating the maximum, median, and minimum). Panel (b) maps the degree to which cities' populations are older (expressed as the 85+ to 20-44 age group ratio) compared to the standard population. Panel (c) shows the ratio of the risk estimated for each city's local population compared to that for the 2013 European standard population. The boxes indicate the median and the first and third quartiles, and the whiskers the minimum/maximum value within 1.5 times the interquartile range from the box. Outliers are not shown. Panel (d) shows the geographical distribution of the age structure impact on the annual net mortality risk estimate.

3 Factors controlling differences between climate groups

There is strong variability in the UHI impact between cities within each Köppen-Geiger climate group and significant overlap between different climate groups (Fig. 2 in the main text). A few notable differences include significantly lower UHI protective effects during cold extreme days for cities with cold climates (Fig. 2c) and a tendency for greater adverse effects during heat extreme days for cities in temperature climate with no dry season and hot summers (Fig. 2b). The latter cities are also more likely to experience an annual net adverse effect from UHIs, while greater proportions of the cities with temperate dry summer climates are likely to experience greater protective effect on average (Fig. 2a).

Some of this difference between climate groups is due to differences in their population age structures. In particular, UHI's impacts during both temperature extremes are notably reduced for the temperate, no dry season, hot summer climate group when population age is standardised (Fig. S5b,c), indicating more vulnerable older populations in these cities. After removing the role of population age structure, all climate groups show similar responses during extreme heat. However, differences persist during cold extremes, some of which can be traced back to difference between the temperature-mortality exposure-response functions (ERFs). For instance, cities for which the protective UHI effect during cold extreme days is less pronounced (the cold climate group and parts of the temperate climate groups with no dry seasons) tend to have ERFs with more gradual increases in risk toward colder temperatures (Fig. S6).

The strength and yearly cycle of UHI, which have been found to differ across climate zones [4], can also influence its impact under different climates. Among climate groups examined in this study, weaker UHIs can be noted with statistical significance (Table S1) for cities with cold climates during cold extreme days compared to all other climate groups (Fig. S7). This additionally contributes to the lower UHI mortality impact in these cities.

Lastly, the annual net balance of adverse and protective effects further de-

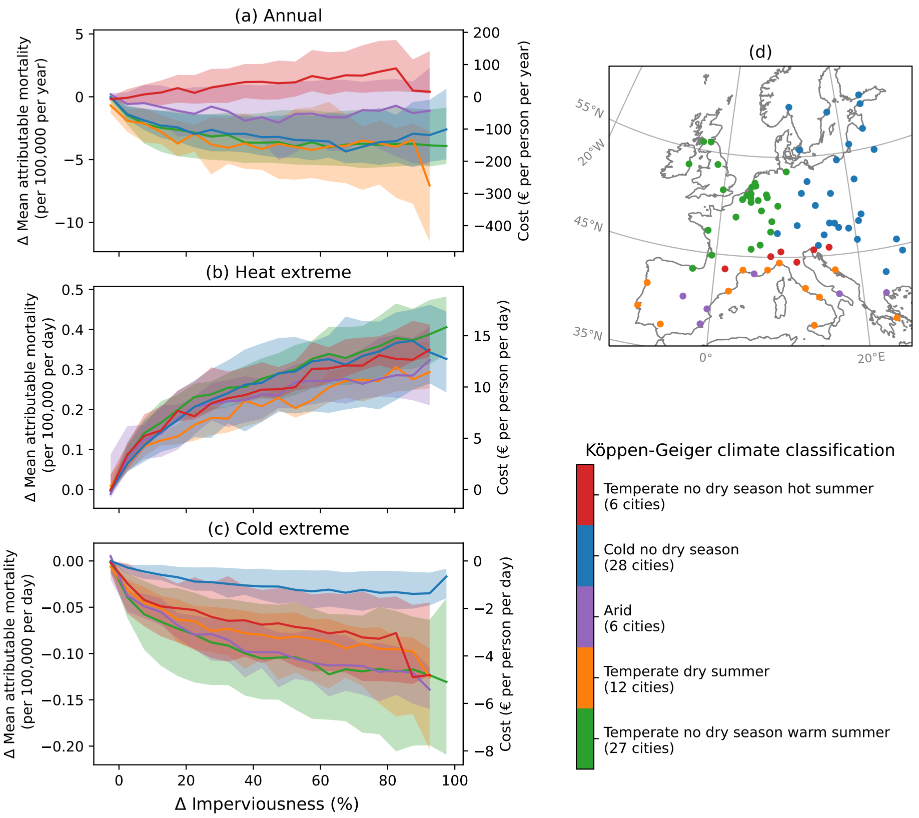

Figure S5: As Fig. 2 in the main text but with population age structures standardised according to the 2013 European standard population.

pends on the balance of cold and warm days observed in each city. This leads to differences, for instance, in the net impact for cities with arid climates compared to those with dry summer climates (Fig. 2a) despite their similar responses to temperature extremes.

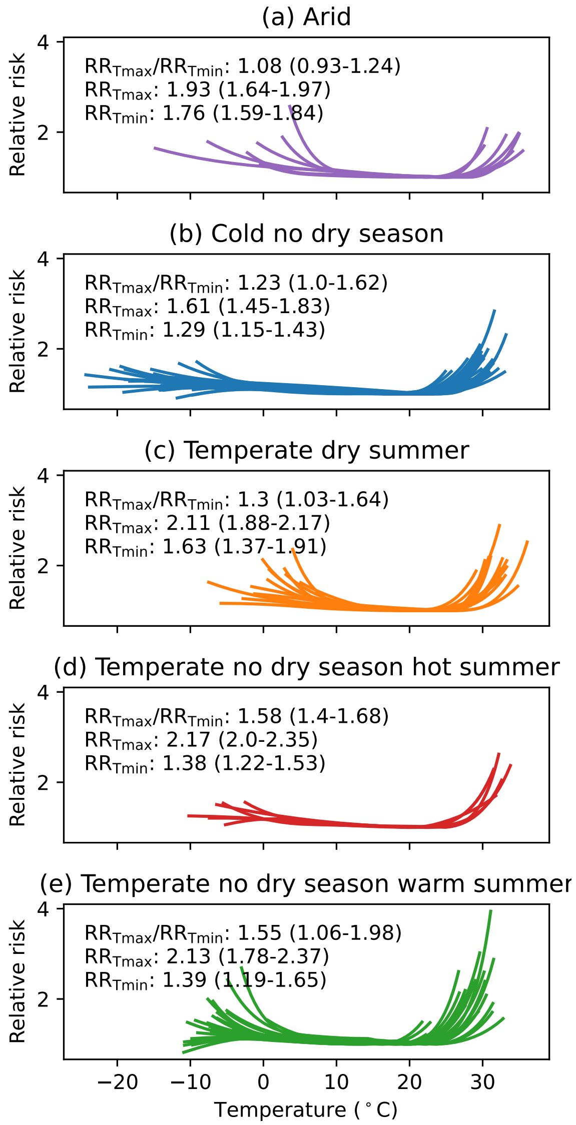

Figure S6: Exposure-response functions for the 65 to 74 age group, grouped by Köppen-Geiger climate classification. Numbers shown in each panel indicate the median and interquartile range of the cities' heat-to-cold vulnerability ratio, expressed as the ratio of the relative risk at each city's maximum temperature (RR_{Tmax}) versus that at the city's minimum temperature (RR_{Tmin}), as well as the cities' RR_{Tmax} and RR_{Tmin} shown separately.

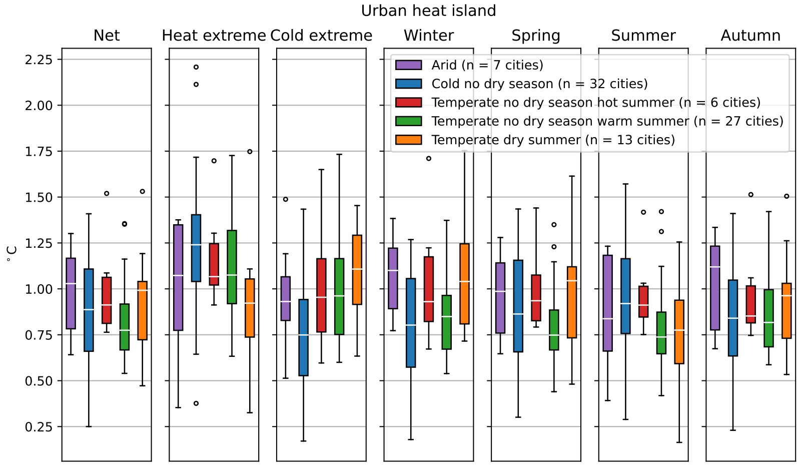

Figure S7: Magnitude of the urban heat island, defined as the difference in average temperature between the urban and rural parts of the UrbClim model domain for each city, during different time subsets. The number of cities in each Köppen-Geiger climate classification group is indicated in the figure legend. The box indicates the first to third quartiles, the white bar the median, the whiskers the minimum/maximum value within 1.5 times the interquartile range from the first/third quartiles, and dots the outliers.

Table S1: Ratio of the two U statistics (lower:higher) from the two-sided Mann-Whitney U test of the urban heat island (UHI) magnitude (difference of urban and rural mean temperatures) between pairs of climate groups. A ratio closer to one indicates greater likelihood that the distributions of UHI magnitudes are identical between the two climate groups. * denotes statistical significance (p-value < 0.1), ** denotes p-value < 0.05. Exact p-values are listed in table S2. Annual average UHI (Net) as well as the UHI during the warmest/coldest 2 % (22) days (Heat/Cold extreme) are examined. Köppen-Geiger climate group names are abbreviated as follows: Cold = cold no dry season, Hot summer = Temperate no dry season hot summer, Warm summer = Temperate no dry season warm summer, Dry summer = Temperate dry summer.

	Arid	Cold	Hot summer	Warm summer	Dry summer
**Net**					
Arid	1.00	0.65	0.91	0.50*	0.69
Cold	0.65	1.00	0.63	0.81	0.82
Hot summer	0.91	0.63	1.00	0.41*	0.81
Warm summer	0.50*	0.81	0.41*	1.00	0.67
Dry summer	0.69	0.82	0.81	0.67	1.00
**Heat extreme**					
Arid	1.00	0.56	0.75	0.77	0.63
Cold	0.56	1.00	0.70	0.67*	0.30**
Hot summer	0.75	0.70	1.00	0.86	0.30**
Warm summer	0.77	0.67*	0.86	1.00	0.42**
Dry summer	0.63	0.30**	0.30**	0.42**	1.00
**Cold extreme**					
Arid	1.00	0.45*	1.00	0.82	0.60
Cold	0.45*	1.00	0.40*	0.34**	0.27**
Hot summer	1.00	0.40*	1.00	0.95	0.70
Warm summer	0.82	0.34**	0.95	1.00	0.77
Dry summer	0.60	0.27**	0.70	0.77	1.00

Table S2: P-values of the two-sided Mann-Whitney U tests in table S1.

	Arid	Cold	Hot summer	Warm summer	Dry summer
**Net**					
Arid	0.474	0.195	0.472	0.093	0.263
Cold	0.195	0.497	0.195	0.249	0.304
Hot summer	0.472	0.195	0.468	0.059	0.379
Warm summer	0.093	0.249	0.059	0.497	0.163
Dry summer	0.263	0.304	0.379	0.163	0.490
**Heat extreme**					
Arid	0.474	0.125	0.361	0.305	0.214
Cold	0.125	0.497	0.255	0.099	0.002
Hot summer	0.361	0.255	0.468	0.399	0.036
Warm summer	0.305	0.099	0.399	0.497	0.020
Dry summer	0.214	0.002	0.036	0.020	0.490
**Cold extreme**					
Arid	0.474	0.064	0.472	0.351	0.192
Cold	0.064	0.497	0.053	0.001	0.001
Hot summer	0.472	0.053	0.468	0.472	0.284
Warm summer	0.351	0.001	0.472	0.497	0.263
Dry summer	0.192	0.001	0.284	0.263	0.490

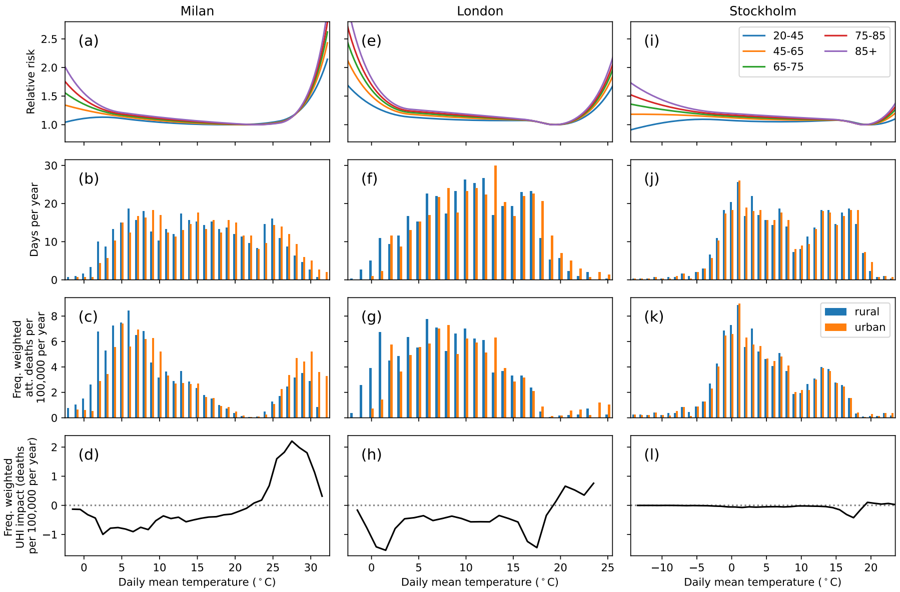

Figure S8: Frequency and magnitude of impact across each city's temperature spectrum for (a-d) Milan, (e-h) London, and (i-l) Stockholm. Panels (a,e,i) show the exposure-response relationship for each age group. Panels (b,f,j) show frequency of occurrence of different daily mean temperatures in the years 2015 to 2017, contrasting the average in urban vs rural areas. Panels (c,g,k) show the frequency weighted temperature-related mortality risk across each city's temperature spectrum. Higher values imply more frequent occurrence and/or greater acute risk. Panels (d,h,l) show the frequency weighted UHI impact on mortality risk as a function of rural mean temperature. Higher values imply greater impact of urban-rural difference, due to more frequent occurrence and/or greater acute risk. Note that this is not the same as the difference between urban and rural values in panels (c,g,k). Rather, the UHI impact is calculated for each day then binned by rural mean temperature.

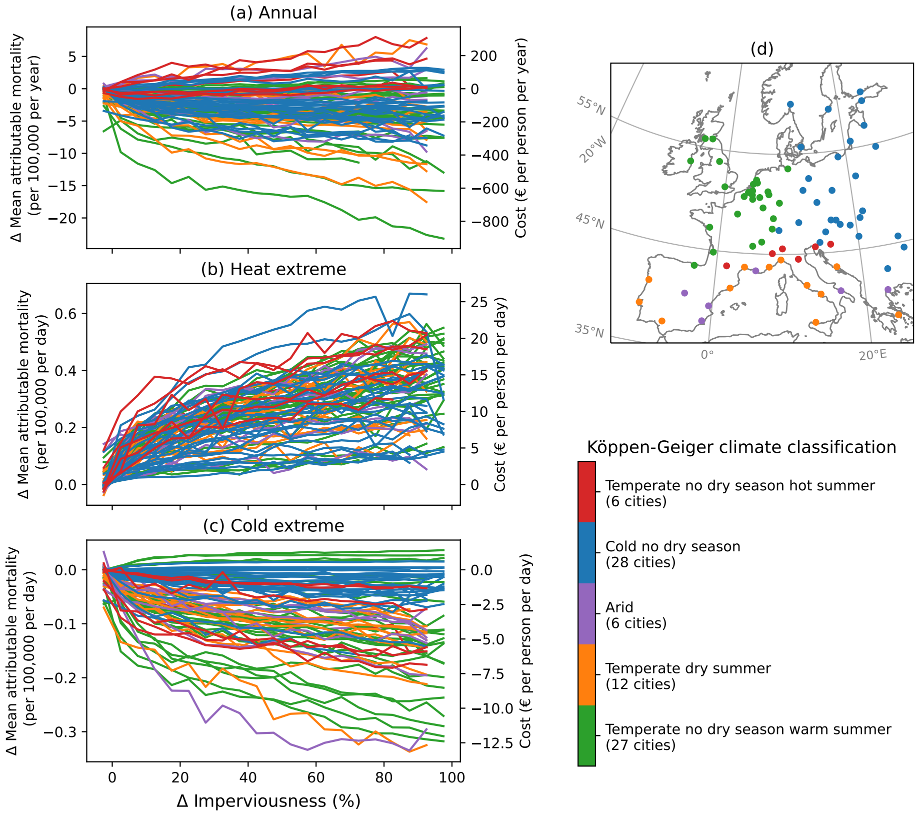

Figure S9: As Fig. 2 in the main text but showing individual cities within each climate group.

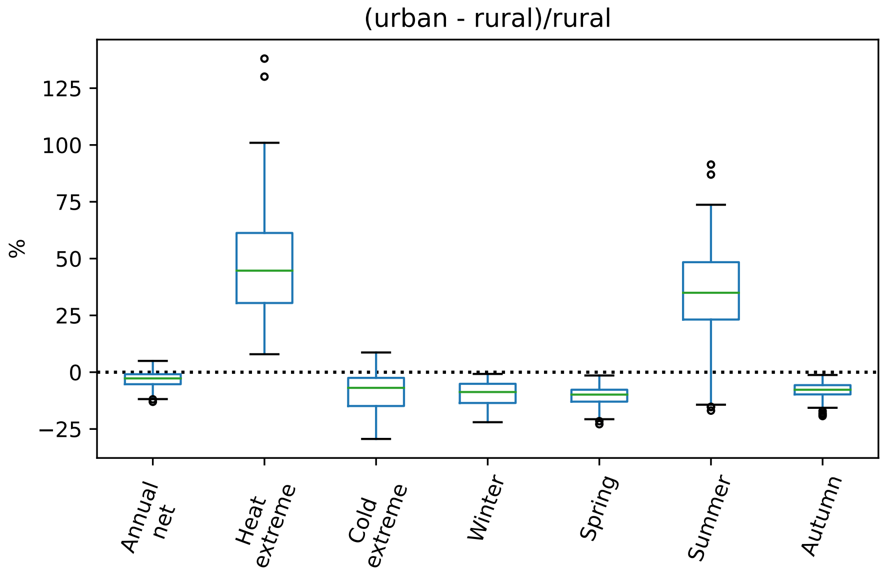

Figure S10: As Fig. 3a in the main text but showing the percentage difference instead.

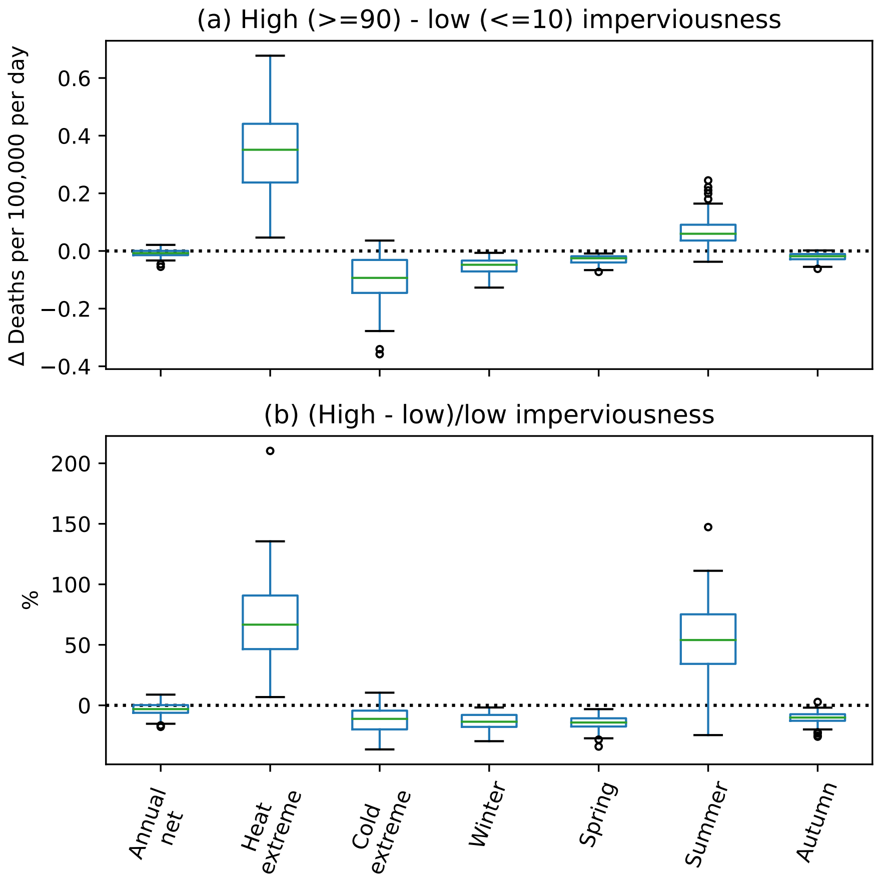

Figure S11: Difference in mortality risk between the most built-up parts of the city (imperviousness $\geq$ 90) and the least built-up parts (imperviousness $\leq$ 10). Boxplots show the spread across 85 European cities, with the box indicating the first to third quartiles, the green line the median, the whiskers the minimum/maximum value within 1.5 times the interquartile range from the first/third quartiles, and dots the outliers. Panel (a) shows the difference and panel (b), the percent difference.

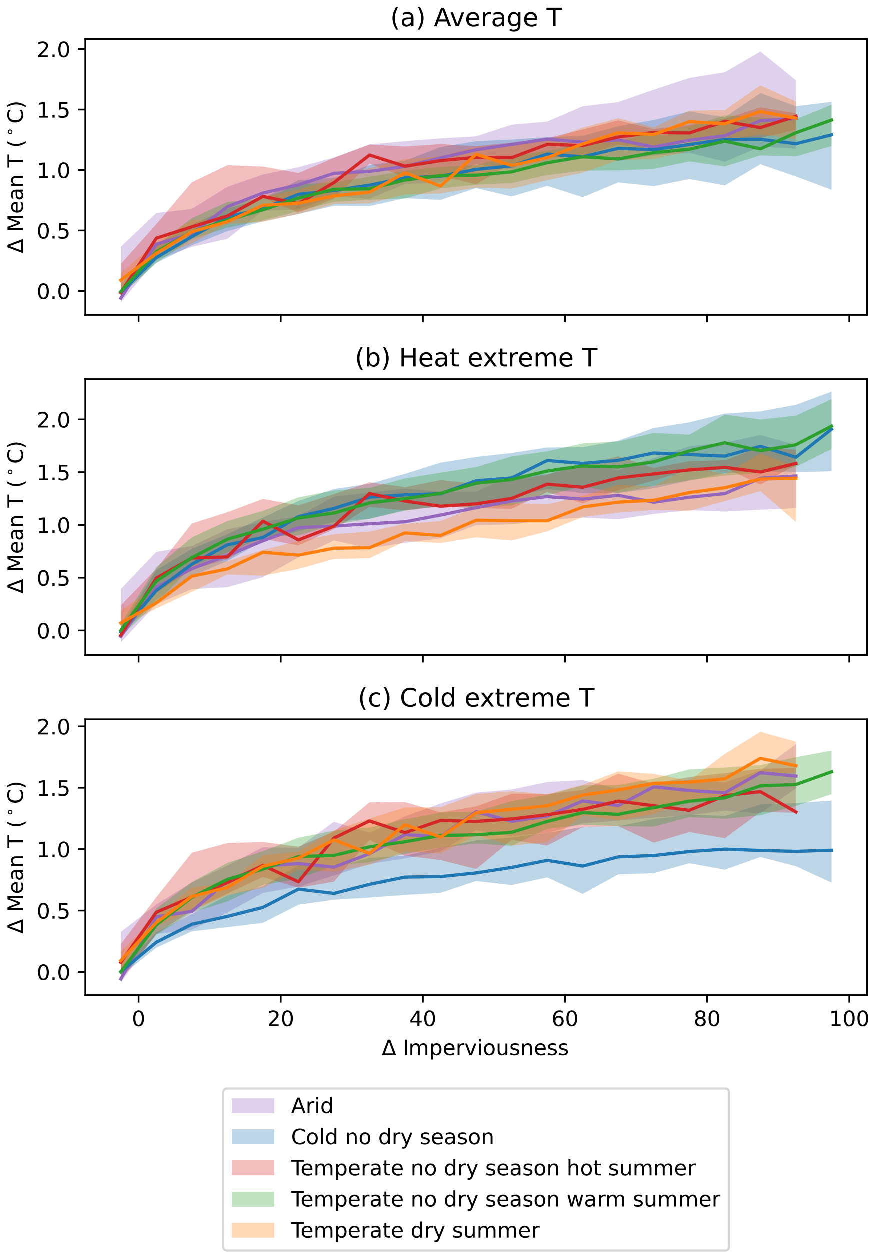

Figure S12: As Fig. 2 in the main text but showing the difference in average air temperature instead of mortality.

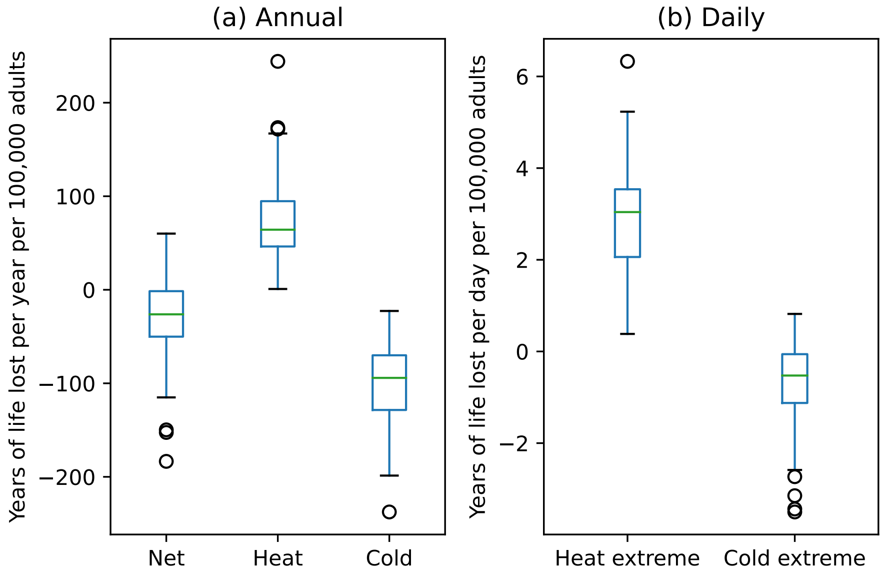

Figure S13: Years of life lost due to UHIs' impact on human mortality. Panel (a) shows the annual total, and panel (b) the daily mean impact of the warmest and coldest 2% days in 2015-2017. Boxplots show the spread of the 85 European cities. Boxes indicate the median and the first and third quartiles, and the whiskers the minimum/maximum value within 1.5 times the interquartile range from the box.

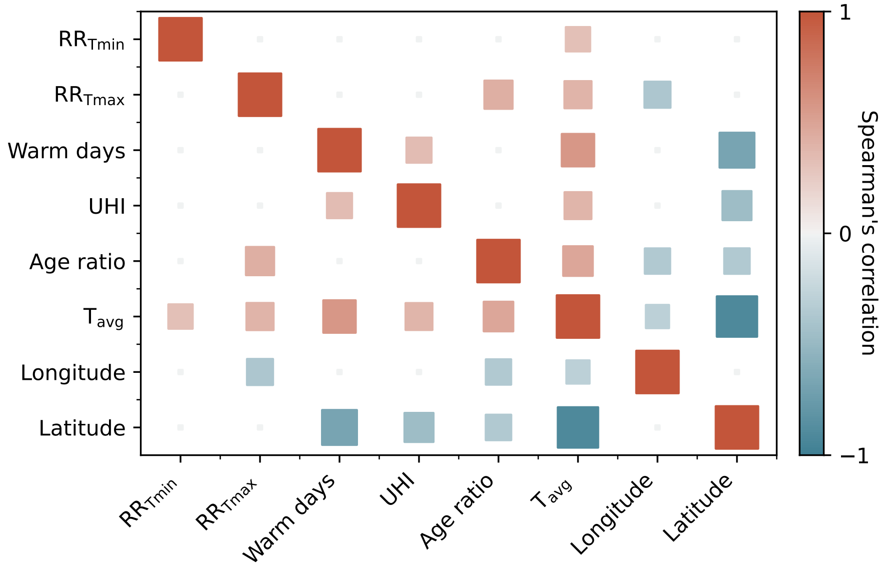

Figure S14: Spearman's correlations between metrics as shown and explained in Fig. 4 in the main text.

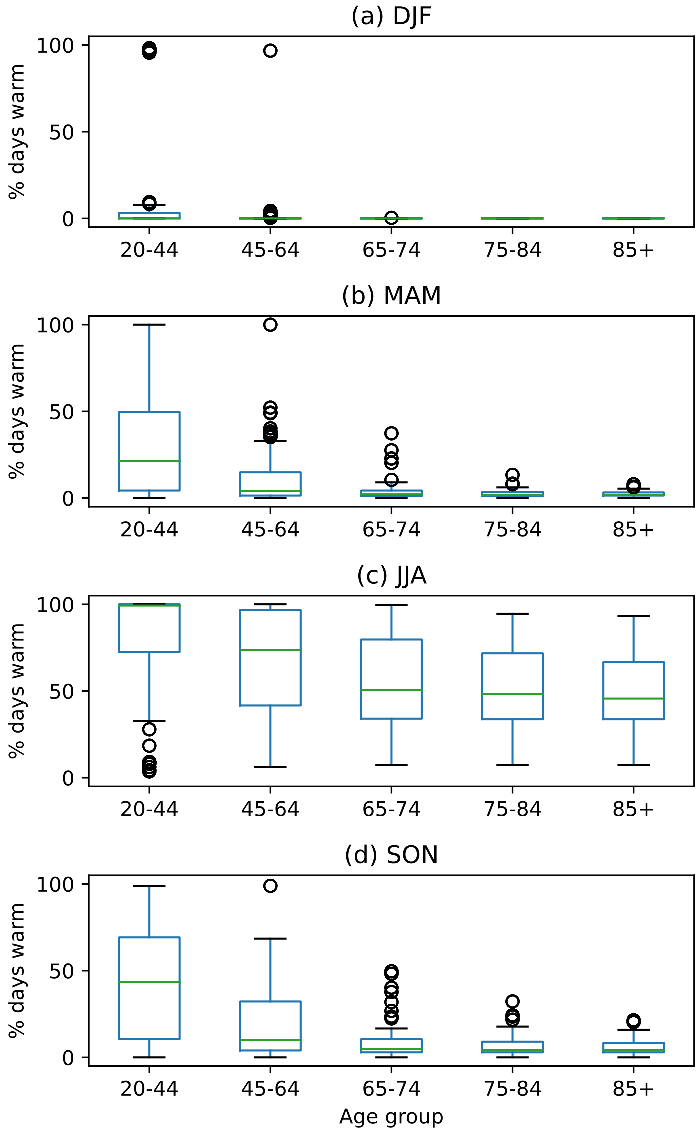

Figure S15: Percentage of (a) winter, (b) spring, (c) summer, and (d) autumn days considered warm (above the age-specific minimum mortality temperature) for each age group. Boxplots show the variation among cities. The box indicates the first to third quartiles, the green line the median, the whiskers the minimum/maximum value within 1.5 times the interquartile range from the first/third quartiles, and dots the outliers.

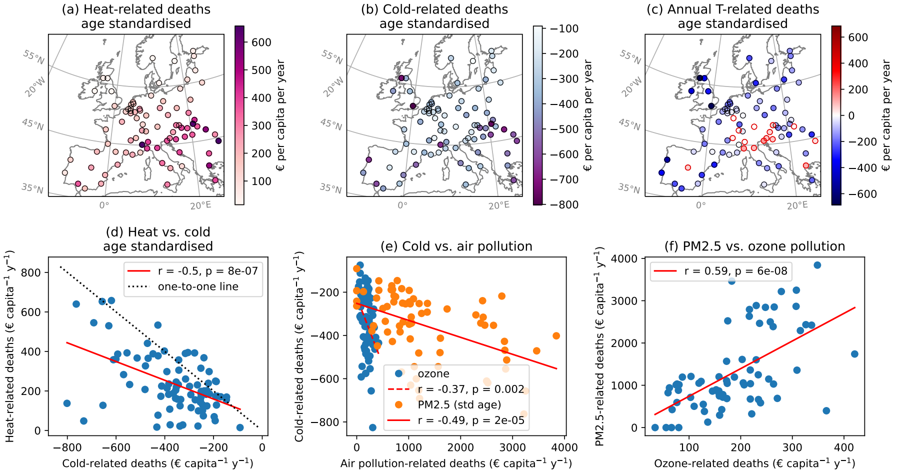

Figure S16: As Fig. 5 in the main text except with population age structure standardised following the 2013 European standard population, except for ozone, and with panel (e) showing comparisons with UHI's impact on cold-related mortality and panel (f) showing comparisons between $PM_{2.5}$ and ozone pollution. Panels (a-c) show the annual net economic impact of UHI-induced mortality. Panels (d-f) show correlations between cities' UHI impact on heat-related mortality, that on cold-related mortality, and air pollution-related mortality.

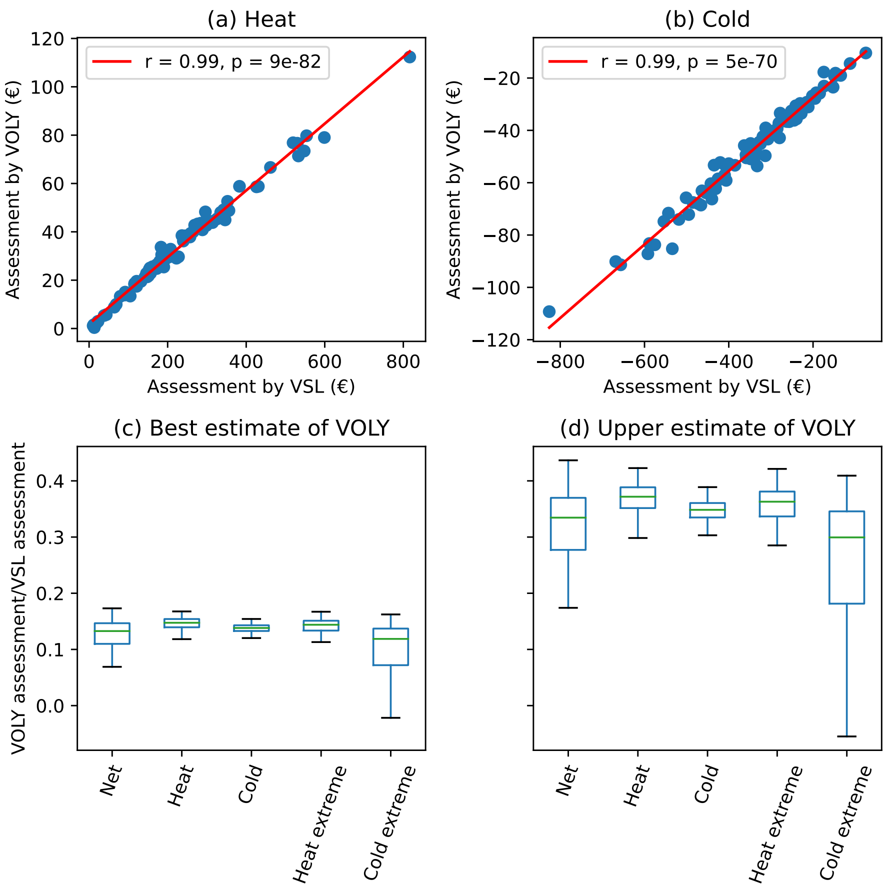

Figure S17: Comparison of economic impact assessments through value of life year (VOLY) and through value of statistical life (VSL) approaches. Panels (a) and (b) show correlations between the two approaches for annual UHI heat- and cold-related impacts. Legends indicate the Spearman's rank correlation values. Panels (c) and (d) show comparisons of the magnitude of the assessed impacts, represented as the VOLY assessment divided by the VSL assessment. In panel (c) the best estimate for VOLY of 46,000 2021-EUR is assumed, while in panel (d) the upper estimate of 116,000 2021-EUR is assumed. Boxes in the boxplots indicate the median and the first and third quartiles, and the whiskers the minimum/maximum value within 1.5 times the interquartile range from the box. Outliers in the boxplots are not shown.

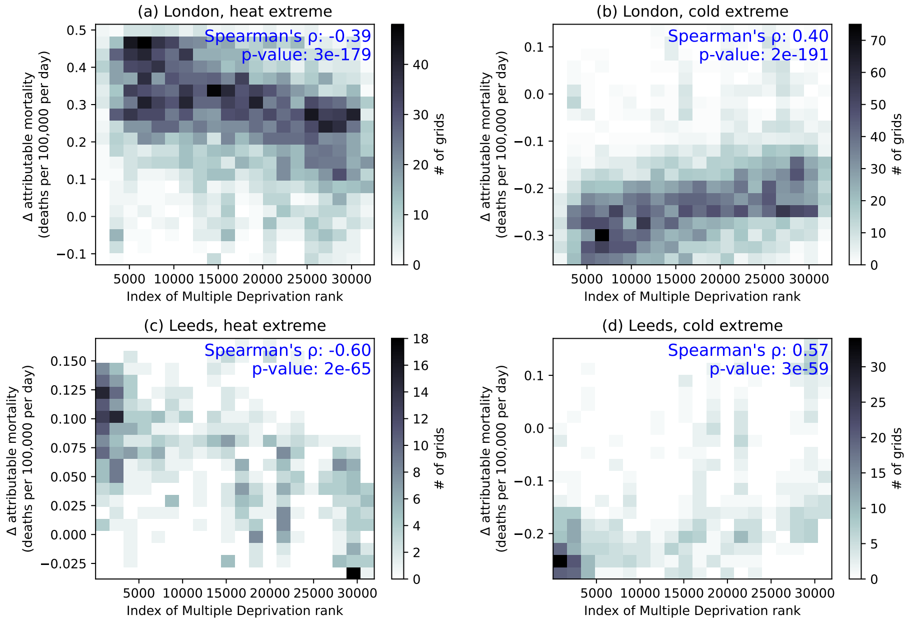

Figure S18: Correlation between socioeconomic deprivation and UHI's impact on mortality risk for (a,b) London and (c,d) Leeds in the UK during the (a,c) warmest and (b,d) coldest 2% days in 2015-2017. UHI impact is shown as the difference in mortality risk compared to the rural average, assuming local population age structure. Socioeconomic deprivation is represented by the 2019 English Index of Multiple Deprivation (IMD, [5], contains public sector information licensed under the Open Government Licence v3.0), which considers income, employment, education, health, crime, housing access, and living environment, at Lower-layer Super Output Areas (LSOA). The index ranks 32,844 LSOA areas in England from most deprived (low IMD rank) to least deprived (high IMD rank).

Table S3: List of cities in each climate group. * indicates cities not included in Fig. 2 in the main text due to having maximum urban-rural imperviousness difference that is less than 80%.

**Climate group**	**Cities**
Arid	Bari, Madrid, Marseille, Murcia, Thessaloniki, Valencia, Varna*
Cold no dry season	Berlin, Brasov*, Bratislava, Bucharest, Budapest, Cluj-Napoca*, Copenhagen, Debrecen*, Gdansk, Graz, Gyor*, Helsinki, Klaipeda*, Kosice*, Krakow*, Leipzig, Ljubljana, Miskolc*, Munich, Oslo, Pecs*, Prague, Riga*, Sofia*, Stockholm, Szeged*, Tallinn, Vienna, Vilnius*, Warsaw, Wroclaw, Zurich
Temperate dry summer	Athens, Barcelona, Genoa, Lisbon, Malaga*, Montpellier, Naples, Nice, Palermo, Porto, Rome, Sevilla, Split*
Temperate no dry season hot summer	Bologna, Milan, Toulouse, Trieste, Turin, Zagreb*
Temperate no dry season warm summer	Amsterdam, Antwerp, Basel, Bilbao, Bordeaux, Brussels, Charleroi, Cologne, Dublin*, Dusseldorf, Edinburgh, Frankfurt am Main, Geneva, Ghent, Glasgow, Hamburg, Leeds, Liege, Lille, London, Luxembourg, Lyon, Nantes, Paris, Rotterdam, Strasbourg, Utrecht

Table S4: Mortality associated with UHI, per 100,000 urban adult population, local population age structure. Values in brackets indicate the 90% confidence interval bounds.

City	Net [per year]	Heat [per year]	Cold [per year]	Heat extreme [per day]	Cold extreme [per day]
Trieste	4.7 (2.3, 7.2)	14.0 (12.0, 15.7)	-9.3 (-10.9, -7.4)	0.36 (0.27, 0.43)	-0.05 (-0.08, -0.02)
Genoa	4.2 (0.8, 7.7)	15.3 (12.5, 17.2)	-11.1 (-13.1, -7.9)	0.31 (0.24, 0.38)	-0.09 (-0.11, -0.07)
Bologna	2.9 (0.9, 5.0)	13.6 (12.3, 14.8)	-10.7 (-12.4, -8.9)	0.3 (0.24, 0.35)	-0.03 (-0.07, 0.02)
Thessaloniki	2.7 (-0.4, 5.7)	13.6 (10.7, 16.1)	-10.9 (-12.8, -8.8)	0.32 (0.21, 0.42)	-0.06 (-0.08, -0.04)
Ljubljana	1.9 (0.1, 3.8)	6.9 (5.4, 8.3)	-5.0 (-6.3, -3.6)	0.18 (0.1, 0.24)	-0.01 (-0.03, 0.02)
Zagreb	1.7 (-0.8, 4.2)	8.9 (6.8, 10.7)	-7.1 (-9.0, -5.3)	0.17 (0.07, 0.26)	-0.02 (-0.05, 0.01)
Madrid	1.4 (-0.6, 3.4)	6.1 (4.8, 7.6)	-4.7 (-6.4, -3.1)	0.25 (0.18, 0.31)	-0.08 (-0.11, -0.04)
Vienna	1.4 (-0.5, 3.1)	6.9 (5.5, 8.5)	-5.5 (-7.0, -4.3)	0.3 (0.23, 0.36)	-0.03 (-0.06, 0.0)
Graz	1.4 (-0.7, 3.4)	5.8 (4.6, 7.6)	-4.5 (-6.4, -3.1)	0.27 (0.19, 0.33)	0.0 (-0.03, 0.04)
Bucharest	0.9 (-2.4, 3.9)	13.3 (10.9, 15.7)	-12.4 (-14.7, -10.3)	0.51 (0.4, 0.61)	-0.03 (-0.06, 0.01)
Geneva	0.5 (-0.4, 1.5)	4.0 (3.3, 4.6)	-3.4 (-4.1, -2.7)	0.18 (0.15, 0.2)	-0.01 (-0.02, 0.01)
Basel	0.4 (-0.9, 1.9)	6.6 (5.7, 7.4)	-6.2 (-7.3, -5.0)	0.33 (0.29, 0.37)	-0.02 (-0.05, 0.02)
Debrecen	0.3 (-2.4, 2.7)	7.6 (5.1, 9.6)	-7.3 (-8.8, -5.9)	0.19 (0.07, 0.3)	-0.02 (-0.04, 0.0)
Luxembourg	0.2 (-0.6, 1.1)	3.1 (2.6, 3.7)	-2.9 (-3.6, -2.2)	0.18 (0.15, 0.2)	0.02 (-0.01, 0.05)
Munich	0.0 (-1.2, 1.5)	3.8 (2.9, 4.7)	-3.8 (-4.8, -2.6)	0.24 (0.18, 0.29)	-0.02 (-0.05, 0.0)
Milan	-0.0 (-2.0, 1.9)	11.8 (10.6, 13.1)	-11.9 (-13.4, -10.2)	0.41 (0.35, 0.48)	-0.13 (-0.18, -0.09)
Frankfurt am Main	-0.2 (-1.0, 0.7)	3.6 (3.2, 4.1)	-3.8 (-4.6, -2.9)	0.21 (0.19, 0.23)	0.03 (0.01, 0.05)
Turin	-0.2 (-3.2, 2.8)	20.9 (18.8, 22.9)	-21.1 (-23.5, -18.7)	0.46 (0.37, 0.55)	-0.15 (-0.21, -0.1)
Kosice	-0.4 (-3.3, 2.0)	7.6 (5.0, 9.6)	-8.0 (-9.6, -6.6)	0.2 (0.09, 0.3)	-0.01 (-0.03, 0.01)
Zurich	-0.7 (-2.0, 0.8)	4.2 (3.4, 5.1)	-4.9 (-6.0, -3.8)	0.21 (0.17, 0.24)	0.01 (-0.01, 0.04)
Liege	-0.7 (-2.1, 0.7)	5.2 (4.4, 6.2)	-5.9 (-7.2, -4.8)	0.32 (0.28, 0.36)	-0.03 (-0.05, -0.0)
Toulouse	-0.7 (-2.1, 0.8)	5.3 (4.4, 6.2)	-6.0 (-7.2, -4.8)	0.27 (0.22, 0.31)	-0.12 (-0.15, -0.09)

Table S4: Mortality associated with UHI, per 100,000 urban adult population, local population age structure. Values in brackets indicate the 90% confidence interval bounds. (continued)

City	Net [per year]	Heat [per year]	Cold [per year]	Heat extreme [per day]	Cold extreme [per day]
Charleroi	-1.0 (-2.3, 0.5)	5.0 (4.1, 6.1)	-6.0 (-7.2, -4.9)	0.28 (0.23, 0.33)	-0.06 (-0.09, -0.02)
Palermo	-1.0 (-2.6, 0.5)	8.0 (6.8, 8.6)	-9.0 (-10.0, -7.4)	0.15 (0.11, 0.19)	-0.05 (-0.06, -0.04)
Cologne	-1.0 (-1.8, -0.2)	4.1 (3.8, 4.6)	-5.2 (-5.9, -4.5)	0.25 (0.23, 0.28)	0.01 (-0.01, 0.04)
Pecs	-1.1 (-4.4, 2.1)	8.8 (6.0, 11.6)	-9.9 (-11.9, -7.7)	0.26 (0.14, 0.37)	-0.02 (-0.06, 0.02)
Marseille	-1.3 (-3.5, 1.1)	7.1 (5.7, 8.3)	-8.4 (-10.2, -6.3)	0.34 (0.28, 0.4)	-0.14 (-0.17, -0.11)
Split	-1.4 (-4.1, 1.1)	9.1 (7.0, 10.9)	-10.5 (-12.7, -8.3)	0.2 (0.1, 0.28)	-0.06 (-0.08, -0.04)
Dusseldorf	-1.4 (-2.4, -0.3)	6.6 (6.1, 7.3)	-8.0 (-8.9, -7.2)	0.37 (0.34, 0.4)	0.03 (-0.01, 0.07)
Stockholm	-1.6 (-1.8, -1.3)	0.3 (0.2, 0.4)	-1.9 (-2.1, -1.6)	0.04 (0.03, 0.05)	-0.01 (-0.01, -0.0)
Murcia	-1.6 (-3.0, -0.2)	4.9 (4.0, 5.8)	-6.5 (-7.5, -5.5)	0.14 (0.09, 0.18)	-0.1 (-0.11, -0.08)
Warsaw	-1.7 (-3.6, 0.2)	4.6 (3.4, 6.0)	-6.3 (-7.8, -4.9)	0.3 (0.21, 0.37)	-0.02 (-0.04, 0.01)
Bilbao	-1.8 (-4.2, 0.9)	6.0 (4.8, 7.6)	-7.9 (-9.9, -6.0)	0.27 (0.21, 0.33)	-0.07 (-0.08, -0.06)
Nice	-1.9 (-3.1, -0.7)	3.9 (3.2, 4.5)	-5.8 (-6.9, -4.6)	0.16 (0.13, 0.18)	-0.05 (-0.05, -0.04)
Varna	-1.9 (-5.8, 1.9)	10.9 (7.5, 14.3)	-12.8 (-15.0, -10.4)	0.29 (0.19, 0.38)	-0.04 (-0.06, -0.02)
Bratislava	-2.0 (-4.1, -0.0)	4.6 (3.2, 6.2)	-6.6 (-8.2, -5.1)	0.2 (0.13, 0.27)	-0.01 (-0.04, 0.02)
Berlin	-2.0 (-3.0, -1.0)	3.9 (3.5, 4.4)	-5.9 (-6.9, -5.0)	0.28 (0.25, 0.31)	-0.01 (-0.03, 0.01)
Krakow	-2.1 (-4.4, 0.1)	6.8 (5.0, 8.6)	-8.9 (-10.4, -7.4)	0.28 (0.19, 0.35)	-0.02 (-0.03, -0.0)
Vilnius	-2.2 (-5.5, 0.7)	2.7 (-0.2, 5.2)	-4.9 (-7.1, -2.9)	0.08 (-0.17, 0.27)	-0.0 (-0.03, 0.04)
Brussels	-2.3 (-3.4, -1.1)	3.7 (3.2, 4.5)	-6.1 (-7.1, -5.1)	0.26 (0.22, 0.29)	-0.08 (-0.1, -0.04)
Budapest	-2.4 (-5.5, 0.4)	8.6 (6.4, 11.0)	-11.0 (-13.4, -8.9)	0.38 (0.27, 0.48)	-0.03 (-0.05, -0.0)
Miskolc	-2.6 (-7.7, 1.9)	14.2 (9.6, 18.3)	-16.8 (-19.9, -13.5)	0.29 (0.09, 0.46)	-0.03 (-0.09, 0.04)
Sofia	-2.8 (-6.0, 0.0)	7.4 (5.2, 9.8)	-10.2 (-12.6, -8.3)	0.28 (0.2, 0.37)	-0.01 (-0.02, 0.01)
Antwerp	-2.9 (-4.4, -1.3)	4.0 (3.3, 4.9)	-6.9 (-8.2, -5.6)	0.29 (0.24, 0.33)	-0.08 (-0.11, -0.04)

Table S4: Mortality associated with UHI, per 100,000 urban adult population, local population age structure. Values in brackets indicate the 90% confidence interval bounds. (continued)

City	Net [per year]	Heat [per year]	Cold [per year]	Heat extreme [per day]	Cold extreme [per day]
Cluj-Napoca	-2.9 (-6.7, 0.5)	11.0 (7.9, 13.8)	-13.9 (-16.2, -11.5)	0.21 (0.09, 0.33)	-0.04 (-0.06, -0.01)
Helsinki	-2.9 (-3.5, -2.3)	1.0 (0.6, 1.3)	-3.9 (-4.4, -3.3)	0.11 (0.07, 0.14)	-0.02 (-0.03, -0.02)
Ghent	-3.0 (-4.6, -1.4)	3.4 (2.7, 4.2)	-6.4 (-7.8, -5.0)	0.25 (0.2, 0.3)	-0.09 (-0.13, -0.04)
Leipzig	-3.0 (-4.4, -1.7)	6.1 (5.3, 7.1)	-9.2 (-10.4, -8.1)	0.25 (0.21, 0.29)	-0.03 (-0.06, 0.0)
Naples	-3.1 (-4.6, -1.6)	5.2 (4.3, 6.1)	-8.3 (-9.6, -7.0)	0.18 (0.13, 0.23)	-0.08 (-0.09, -0.06)
Riga	-3.2 (-5.4, -1.2)	1.8 (0.4, 3.3)	-5.0 (-6.7, -3.4)	0.05 (-0.07, 0.16)	-0.02 (-0.03, -0.0)
Gyor	-3.3 (-6.8, 0.0)	8.0 (5.5, 10.9)	-11.3 (-13.8, -9.3)	0.28 (0.16, 0.4)	-0.06 (-0.09, -0.01)
Amsterdam	-3.4 (-5.4, -1.1)	3.0 (2.2, 4.3)	-6.3 (-8.2, -4.7)	0.23 (0.16, 0.29)	-0.1 (-0.14, -0.05)
Hamburg	-3.5 (-4.5, -2.5)	2.5 (2.0, 3.0)	-6.0 (-7.0, -5.1)	0.22 (0.18, 0.27)	-0.09 (-0.1, -0.07)
Lille	-3.6 (-5.2, -2.0)	4.4 (3.7, 5.3)	-8.0 (-9.6, -6.6)	0.26 (0.2, 0.3)	-0.13 (-0.18, -0.06)
Tallinn	-3.8 (-5.1, -2.4)	1.6 (0.9, 2.5)	-5.4 (-6.7, -4.4)	0.14 (0.05, 0.22)	-0.02 (-0.03, -0.01)
Gdansk	-3.8 (-5.7, -1.9)	2.3 (1.5, 3.5)	-6.1 (-7.8, -4.6)	0.16 (0.1, 0.21)	-0.04 (-0.06, -0.01)
Paris	-3.8 (-6.2, -1.2)	4.7 (3.8, 5.7)	-8.5 (-10.8, -6.1)	0.32 (0.27, 0.36)	-0.13 (-0.19, -0.07)
Nantes	-3.9 (-5.3, -2.4)	4.7 (4.1, 5.4)	-8.6 (-9.9, -7.4)	0.26 (0.22, 0.29)	-0.12 (-0.16, -0.06)
Prague	-4.0 (-7.1, -1.1)	4.9 (3.3, 6.6)	-8.9 (-11.6, -6.4)	0.27 (0.18, 0.35)	-0.0 (-0.05, 0.05)
Oslo	-4.1 (-5.3, -2.8)	0.3 (0.0, 0.6)	-4.4 (-5.7, -2.9)	0.06 (0.02, 0.09)	0.0 (-0.01, 0.02)
Wroclaw	-4.1 (-6.0, -2.2)	4.4 (3.3, 5.8)	-8.6 (-10.1, -7.3)	0.18 (0.13, 0.24)	-0.04 (-0.06, -0.01)
Utrecht	-4.2 (-6.1, -2.1)	3.9 (3.1, 5.0)	-8.0 (-9.8, -6.5)	0.26 (0.19, 0.31)	-0.13 (-0.17, -0.07)
Rome	-4.2 (-5.7, -2.8)	9.0 (8.1, 10.0)	-13.3 (-14.5, -12.0)	0.25 (0.2, 0.29)	-0.09 (-0.11, -0.07)
Rotterdam	-4.4 (-6.3, -2.4)	3.7 (3.0, 5.0)	-8.2 (-10.0, -6.6)	0.26 (0.2, 0.31)	-0.1 (-0.14, -0.05)
Lyon	-4.7 (-6.8, -2.6)	7.3 (5.7, 8.8)	-11.9 (-13.5, -10.3)	0.34 (0.27, 0.41)	-0.21 (-0.24, -0.18)
Bari	-4.8 (-5.9, -3.6)	3.1 (2.7, 3.5)	-7.9 (-9.0, -6.7)	0.08 (0.06, 0.09)	-0.07 (-0.09, -0.06)

Table S4: Mortality associated with UHI, per 100,000 urban adult population, local population age structure. Values in brackets indicate the 90% confidence interval bounds. (continued)

City	Net [per year]	Heat [per year]	Cold [per year]	Heat extreme [per day]	Cold extreme [per day]
Montpellier	-4.8 (-6.3, -3.2)	6.2 (5.4, 7.2)	-11.0 (-12.4, -9.8)	0.32 (0.27, 0.36)	-0.1 (-0.13, -0.06)
Sevilla	-5.0 (-7.3, -2.8)	7.6 (6.1, 9.2)	-12.7 (-14.5, -10.8)	0.18 (0.09, 0.26)	-0.28 (-0.32, -0.24)
Malaga	-5.2 (-6.9, -3.4)	3.7 (3.1, 4.4)	-8.9 (-10.5, -7.3)	0.13 (0.1, 0.17)	-0.11 (-0.13, -0.1)
Athens	-5.3 (-8.1, -2.4)	9.8 (8.0, 11.6)	-15.0 (-17.6, -12.6)	0.38 (0.31, 0.44)	-0.05 (-0.08, -0.03)
Bordeaux	-5.6 (-6.9, -4.3)	4.8 (4.0, 5.7)	-10.4 (-11.5, -9.2)	0.32 (0.26, 0.36)	-0.18 (-0.21, -0.15)
Szeged	-5.8 (-9.5, -2.4)	8.3 (5.3, 11.3)	-14.2 (-16.7, -11.7)	0.22 (0.11, 0.33)	-0.11 (-0.15, -0.06)
Brasov	-5.9 (-10.3, -1.8)	5.7 (2.3, 9.2)	-11.6 (-14.2, -9.2)	0.18 (0.01, 0.34)	-0.04 (-0.06, -0.01)
Copenhagen	-6.1 (-7.0, -5.2)	0.6 (0.4, 0.9)	-6.7 (-7.5, -5.9)	0.07 (0.05, 0.1)	-0.1 (-0.13, -0.08)
Klaipeda	-6.6 (-9.9, -3.0)	2.6 (0.6, 5.1)	-9.2 (-12.1, -6.7)	0.11 (-0.09, 0.27)	-0.05 (-0.08, -0.01)
Strasbourg	-6.6 (-8.0, -5.3)	3.9 (3.0, 4.8)	-10.5 (-11.6, -9.4)	0.25 (0.19, 0.3)	-0.17 (-0.2, -0.13)
Leeds	-6.7 (-7.4, -6.1)	0.5 (0.3, 0.6)	-7.2 (-7.8, -6.5)	0.06 (0.04, 0.08)	-0.19 (-0.22, -0.17)
Valencia	-6.9 (-8.7, -4.9)	5.2 (4.1, 6.3)	-12.1 (-13.6, -10.4)	0.19 (0.13, 0.25)	-0.28 (-0.31, -0.25)
Dublin	-6.9 (-7.4, -6.3)	0.3 (0.1, 0.5)	-7.1 (-7.7, -6.6)	0.04 (0.02, 0.06)	-0.07 (-0.07, -0.06)
Edinburgh	-7.1 (-8.1, -6.0)	0.6 (0.4, 0.9)	-7.7 (-8.7, -6.7)	0.08 (0.06, 0.1)	-0.11 (-0.12, -0.09)
Barcelona	-8.4 (-10.6, -6.2)	6.3 (5.1, 7.6)	-14.7 (-16.8, -12.6)	0.3 (0.24, 0.34)	-0.15 (-0.18, -0.13)
Lisbon	-9.2 (-10.3, -8.1)	2.0 (1.8, 2.4)	-11.3 (-12.3, -10.2)	0.1 (0.09, 0.11)	-0.11 (-0.13, -0.09)
London	-11.3 (-13.2, -9.1)	2.3 (1.7, 3.0)	-13.7 (-15.4, -11.6)	0.27 (0.21, 0.33)	-0.23 (-0.27, -0.18)
Porto	-12.2 (-13.5, -10.7)	3.0 (2.5, 3.5)	-15.1 (-16.4, -13.9)	0.15 (0.13, 0.18)	-0.15 (-0.17, -0.12)
Glasgow	-16.0 (-17.9, -13.8)	1.1 (0.8, 1.5)	-17.1 (-19.0, -14.9)	0.14 (0.1, 0.18)	-0.24 (-0.28, -0.19)

Table S5: Economic impact of mortality associated with UHI, in 2021-EUR per urban adult inhabitant, local population age structure. Values in brackets indicate the 90% confidence interval bounds.

City	Net [EUR per year]	Heat [EUR per year]	Cold [EUR per year]	Heat extreme [EUR per day]	Cold extreme [EUR per day]
Trieste	184.4 (91.0, 281.9)	547.7 (469.3, 614.7)	-363.2 (-425.1, -289.4)	14.0 (10.48, 16.95)	-2.0 (-3.09, -0.87)
Genoa	164.1 (31.4, 300.0)	599.0 (487.7, 672.8)	-434.8 (-513.8, -308.5)	12.26 (9.32, 15.02)	-3.5 (-4.36, -2.64)
Bologna	112.6 (35.2, 193.7)	532.9 (482.1, 578.9)	-420.3 (-486.2, -349.9)	11.71 (9.4, 13.77)	-0.98 (-2.92, 0.94)
Thessaloniki	104.6 (-13.7, 221.6)	530.1 (419.5, 627.8)	-425.5 (-500.6, -342.7)	12.67 (8.26, 16.52)	-2.35 (-2.94, -1.74)
Ljubljana	73.3 (5.3, 147.7)	268.7 (212.0, 324.3)	-195.4 (-245.9, -140.7)	6.91 (3.92, 9.36)	-0.27 (-1.29, 0.76)
Zagreb	68.3 (-30.0, 165.8)	346.3 (264.4, 420.0)	-278.0 (-353.7, -207.2)	6.66 (2.72, 10.09)	-0.77 (-1.82, 0.31)
Madrid	55.7 (-24.2, 131.8)	240.0 (189.5, 296.2)	-184.4 (-249.8, -122.9)	9.9 (7.14, 12.13)	-2.95 (-4.41, -1.54)
Vienna	55.4 (-20.7, 122.8)	270.2 (214.5, 330.4)	-214.7 (-273.0, -168.2)	11.75 (8.88, 13.93)	-1.14 (-2.15, 0.01)
Graz	53.2 (-27.5, 131.7)	227.6 (178.3, 299.1)	-174.5 (-249.8, -122.9)	10.71 (7.53, 13.05)	0.17 (-1.16, 1.62)
Bucharest	34.6 (-94.8, 150.7)	519.4 (425.6, 614.9)	-484.8 (-575.3, -403.7)	19.92 (15.68, 23.79)	-1.11 (-2.38, 0.4)
Geneva	20.7 (-14.5, 59.5)	155.1 (130.6, 178.5)	-134.4 (-161.7, -104.3)	6.96 (5.79, 7.91)	-0.34 (-0.91, 0.29)
Basel	15.9 (-35.3, 73.6)	256.9 (223.4, 288.8)	-241.0 (-283.8, -194.4)	12.97 (11.41, 14.31)	-0.71 (-1.9, 0.61)
Debrecen	13.5 (-95.7, 105.4)	298.6 (201.3, 376.9)	-285.1 (-342.7, -231.5)	7.62 (2.69, 11.74)	-0.71 (-1.48, 0.16)
Luxembourg	8.9 (-24.1, 44.4)	121.0 (101.7, 143.7)	-112.1 (-140.8, -84.4)	6.88 (5.94, 7.68)	0.65 (-0.42, 1.9)
Munich	1.4 (-48.0, 57.4)	148.2 (111.9, 182.8)	-146.8 (-186.1, -102.5)	9.41 (7.14, 11.22)	-0.87 (-1.81, 0.12)
Milan	-1.9 (-78.1, 74.3)	461.7 (416.1, 511.5)	-463.6 (-525.0, -399.4)	16.16 (13.58, 18.68)	-5.21 (-6.88, -3.6)
Frankfurt am Main	-7.0 (-37.8, 29.1)	142.2 (125.7, 160.5)	-149.3 (-178.3, -112.7)	8.26 (7.33, 9.03)	1.17 (0.45, 1.94)
Turin	-9.7 (-125.6, 109.6)	816.4 (735.0, 896.4)	-826.1 (-917.0, -731.9)	18.02 (14.35, 21.43)	-5.99 (-8.16, -3.9)
Kosice	-17.3 (-130.9, 77.4)	296.1 (197.3, 376.4)	-313.3 (-374.1, -256.2)	8.01 (3.54, 11.74)	-0.47 (-1.18, 0.33)
Zurich	-27.2 (-76.7, 29.4)	165.1 (134.5, 200.2)	-192.3 (-236.0, -149.1)	8.15 (6.6, 9.54)	0.52 (-0.5, 1.56)
Liege	-27.4 (-81.1, 27.7)	203.3 (173.3, 241.2)	-230.7 (-280.0, -188.9)	12.54 (10.75, 14.11)	-1.22 (-2.12, -0.14)

Table S5: Economic impact of mortality associated with UHI, in 2021-EUR per urban adult inhabitant, local population age structure. Values in brackets indicate the 90% confidence interval bounds. (continued)

City	Net [EUR per year]	Heat [EUR per year]	Cold [EUR per year]	Heat extreme [EUR per day]	Cold extreme [EUR per day]
Toulouse	-27.5 (-82.5, 29.5)	207.5 (171.9, 242.6)	-235.0 (-282.5, -189.3)	10.39 (8.57, 12.03)	-4.75 (-5.73, -3.54)
Charleroi	-37.3 (-90.6, 19.9)	196.3 (162.2, 238.7)	-233.7 (-280.1, -190.8)	11.12 (9.06, 12.75)	-2.23 (-3.47, -0.75)
Palermo	-38.8 (-100.4, 21.4)	314.1 (264.8, 335.5)	-352.9 (-392.9, -287.8)	5.84 (4.16, 7.28)	-2.03 (-2.28, -1.75)
Cologne	-39.8 (-70.3, -8.3)	161.7 (147.6, 179.4)	-201.5 (-229.4, -175.1)	9.9 (8.92, 10.76)	0.58 (-0.34, 1.63)
Pecs	-43.0 (-170.9, 81.3)	342.4 (234.0, 451.9)	-385.4 (-465.8, -302.0)	10.23 (5.53, 14.31)	-0.88 (-2.25, 0.68)
Marseille	-50.6 (-137.0, 42.0)	276.7 (223.8, 322.9)	-327.3 (-400.6, -244.9)	13.47 (11.04, 15.67)	-5.53 (-6.52, -4.4)
Split	-52.8 (-158.9, 43.8)	356.1 (274.6, 425.2)	-408.9 (-497.2, -324.9)	7.71 (3.9, 10.85)	-2.42 (-3.1, -1.69)
Dusseldorf	-54.9 (-94.3, -11.3)	258.7 (239.1, 286.6)	-313.6 (-349.5, -280.8)	14.52 (13.24, 15.69)	1.01 (-0.38, 2.6)
Stockholm	-62.8 (-72.3, -51.5)	12.0 (8.4, 15.7)	-74.7 (-84.0, -63.8)	1.5 (1.09, 1.92)	-0.22 (-0.28, -0.15)
Murcia	-63.5 (-116.9, -9.7)	191.6 (154.7, 228.2)	-255.2 (-294.6, -213.3)	5.53 (3.56, 7.16)	-3.75 (-4.3, -3.17)
Warsaw	-65.0 (-141.9, 7.6)	180.0 (131.8, 236.4)	-245.0 (-306.4, -192.7)	11.69 (8.37, 14.46)	-0.62 (-1.41, 0.28)
Bilbao	-70.8 (-164.4, 33.4)	236.4 (187.7, 295.5)	-307.2 (-385.3, -236.2)	10.64 (8.26, 12.8)	-2.83 (-3.3, -2.34)
Nice	-74.0 (-120.9, -26.4)	153.9 (126.0, 174.3)	-227.9 (-268.8, -180.6)	6.16 (5.14, 7.08)	-1.81 (-2.07, -1.51)
Varna	-74.9 (-227.4, 76.2)	426.3 (291.6, 557.4)	-501.2 (-587.5, -404.7)	11.21 (7.32, 15.03)	-1.49 (-2.16, -0.71)
Bratislava	-76.9 (-162.0, -1.8)	179.6 (123.5, 242.7)	-256.5 (-322.3, -200.9)	7.88 (4.89, 10.39)	-0.43 (-1.54, 0.73)
Berlin	-79.1 (-117.5, -37.3)	153.0 (137.0, 171.9)	-232.1 (-268.0, -196.4)	11.03 (9.81, 12.04)	-0.24 (-0.98, 0.54)
Krakow	-82.5 (-170.9, 3.1)	264.6 (197.1, 334.7)	-347.1 (-407.5, -290.6)	10.84 (7.55, 13.7)	-0.64 (-1.19, -0.02)
Vilnius	-87.4 (-213.8, 28.8)	104.7 (-5.9, 202.9)	-192.2 (-279.4, -112.0)	2.96 (-6.77, 10.61)	-0.02 (-1.28, 1.64)
Brussels	-90.6 (-133.9, -44.2)	146.6 (125.6, 174.0)	-237.2 (-276.0, -201.0)	10.12 (8.64, 11.34)	-2.94 (-3.99, -1.63)
Budapest	-94.2 (-213.4, 15.6)	335.0 (248.9, 428.3)	-429.3 (-523.0, -347.5)	14.98 (10.59, 18.75)	-1.13 (-2.13, -0.03)
Miskolc	-102.7 (-302.3, 73.9)	553.8 (376.7, 714.8)	-656.5 (-779.8, -529.8)	11.29 (3.54, 17.82)	-1.09 (-3.39, 1.38)

Table S5: Economic impact of mortality associated with UHI, in 2021-EUR per urban adult inhabitant, local population age structure. Values in brackets indicate the 90% confidence interval bounds. (continued)

City	Net [EUR per year]	Heat [EUR per year]	Cold [EUR per year]	Heat extreme [EUR per day]	Cold extreme [EUR per day]
Sofia	-111.4 (-236.5, 0.2)	288.6 (204.4, 381.4)	-400.0 (-493.2, -323.5)	11.1 (7.83, 14.3)	-0.21 (-0.69, 0.29)
Antwerp	-111.8 (-173.1, -51.2)	156.4 (128.5, 190.4)	-267.9 (-322.3, -218.8)	11.29 (9.2, 13.04)	-3.05 (-4.44, -1.51)
Cluj-Napoca	-112.2 (-263.6, 19.7)	430.9 (307.8, 537.9)	-543.2 (-633.5, -448.5)	8.09 (3.36, 12.9)	-1.47 (-2.5, -0.31)
Helsinki	-113.9 (-135.1, -90.4)	38.4 (23.6, 52.3)	-152.4 (-172.8, -130.1)	4.27 (2.81, 5.63)	-0.86 (-1.08, -0.63)
Ghent	-119.2 (-180.3, -54.6)	132.3 (105.2, 165.8)	-251.5 (-304.5, -196.7)	9.89 (7.93, 11.61)	-3.36 (-5.09, -1.39)
Leipzig	-119.2 (-170.8, -68.0)	239.9 (207.2, 276.8)	-359.2 (-408.1, -315.1)	9.75 (8.18, 11.16)	-1.23 (-2.38, 0.04)
Naples	-121.6 (-179.1, -63.7)	203.1 (169.4, 238.9)	-324.8 (-376.8, -274.5)	7.15 (5.19, 8.89)	-2.99 (-3.48, -2.46)
Riga	-126.4 (-209.4, -46.0)	69.1 (15.7, 129.9)	-195.5 (-261.4, -133.3)	2.04 (-2.79, 6.2)	-0.71 (-1.27, -0.05)
Gyor	-128.0 (-265.0, 0.4)	314.3 (215.6, 425.0)	-442.4 (-539.9, -361.8)	11.13 (6.39, 15.6)	-2.19 (-3.68, -0.35)
Amsterdam	-131.7 (-212.4, -44.9)	115.9 (85.6, 167.1)	-247.5 (-321.6, -183.8)	9.14 (6.42, 11.39)	-4.01 (-5.49, -2.14)
Hamburg	-138.1 (-175.5, -97.3)	97.2 (78.5, 119.2)	-235.4 (-272.4, -197.9)	8.78 (7.06, 10.47)	-3.32 (-3.95, -2.6)
Lille	-140.1 (-204.3, -77.3)	172.3 (146.6, 207.1)	-312.4 (-373.7, -257.6)	9.98 (7.93, 11.69)	-4.92 (-6.93, -2.44)
Tallinn	-147.2 (-198.5, -94.5)	64.2 (35.3, 99.5)	-211.4 (-260.9, -170.9)	5.62 (2.12, 8.65)	-0.82 (-1.25, -0.34)
Gdansk	-148.4 (-222.4, -73.1)	90.9 (58.0, 136.6)	-239.3 (-303.9, -181.8)	6.19 (3.8, 8.3)	-1.53 (-2.43, -0.48)
Paris	-149.4 (-242.7, -46.8)	183.2 (147.0, 222.9)	-332.6 (-420.6, -237.8)	12.42 (10.5, 13.97)	-5.25 (-7.36, -2.9)
Nantes	-151.2 (-209.0, -94.0)	184.7 (159.2, 210.2)	-336.0 (-386.6, -288.0)	10.2 (8.63, 11.47)	-4.58 (-6.31, -2.54)
Prague	-157.9 (-278.0, -43.1)	190.2 (129.5, 258.4)	-348.2 (-454.3, -251.0)	10.63 (7.06, 13.86)	-0.01 (-1.88, 1.87)
Oslo	-160.6 (-205.3, -110.6)	13.2 (0.5, 23.7)	-173.8 (-221.0, -114.6)	2.18 (0.77, 3.44)	0.1 (-0.42, 0.68)
Wroclaw	-162.2 (-235.0, -87.3)	172.4 (127.1, 224.9)	-334.7 (-395.6, -284.3)	7.2 (5.16, 9.24)	-1.44 (-2.34, -0.32)
Utrecht	-162.6 (-240.3, -83.8)	151.4 (120.3, 195.6)	-314.0 (-383.4, -254.6)	10.09 (7.53, 12.1)	-4.89 (-6.65, -2.75)
Rome	-165.8 (-221.6, -108.6)	352.6 (315.5, 391.4)	-518.4 (-566.6, -469.0)	9.59 (7.8, 11.2)	-3.49 (-4.24, -2.65)

Table S5: Economic impact of mortality associated with UHI, in 2021-EUR per urban adult inhabitant, local population age structure. Values in brackets indicate the 90% confidence interval bounds. (continued)

City	Net [EUR per year]	Heat [EUR per year]	Cold [EUR per year]	Heat extreme [EUR per day]	Cold extreme [EUR per day]
Rotterdam	-172.4 (-248.0, -93.1)	146.5 (117.0, 194.4)	-318.9 (-390.4, -259.4)	10.26 (7.87, 12.23)	-3.96 (-5.63, -1.9)
Lyon	-182.0 (-265.4, -102.0)	284.1 (222.5, 343.3)	-466.2 (-527.9, -402.7)	13.37 (10.41, 16.01)	-8.27 (-9.28, -7.15)
Bari	-185.9 (-232.4, -141.1)	121.0 (104.4, 137.5)	-307.0 (-351.2, -261.1)	3.03 (2.25, 3.68)	-2.84 (-3.45, -2.21)
Montpellier	-187.0 (-246.9, -127.0)	244.1 (211.7, 280.4)	-431.1 (-483.4, -382.3)	12.5 (10.65, 14.09)	-3.86 (-5.21, -2.28)
Sevilla	-196.2 (-287.0, -110.8)	298.9 (237.8, 358.2)	-495.1 (-566.9, -422.1)	6.9 (3.56, 9.98)	-10.93 (-12.7, -9.19)
Malaga	-202.3 (-271.0, -131.4)	146.1 (122.1, 172.9)	-348.5 (-412.4, -284.1)	5.19 (3.79, 6.47)	-4.41 (-5.06, -3.75)
Athens	-205.4 (-317.5, -92.5)	382.9 (311.3, 454.7)	-588.3 (-686.7, -494.1)	14.86 (12.25, 17.24)	-2.12 (-2.96, -1.22)
Bordeaux	-217.5 (-271.1, -166.5)	188.6 (154.6, 222.1)	-406.2 (-451.5, -358.0)	12.33 (10.19, 14.14)	-7.05 (-8.13, -5.8)
Szeged	-228.1 (-373.0, -93.9)	325.9 (208.8, 441.9)	-554.0 (-652.7, -456.1)	8.61 (4.23, 12.8)	-4.27 (-5.97, -2.2)
Brasov	-231.8 (-403.1, -71.8)	222.4 (91.0, 361.4)	-454.3 (-555.6, -361.2)	7.11 (0.42, 13.42)	-1.48 (-2.31, -0.57)
Copenhagen	-239.9 (-273.1, -204.9)	22.3 (13.7, 36.9)	-262.3 (-295.1, -231.1)	2.93 (1.76, 3.96)	-4.07 (-4.94, -3.04)
Klaipeda	-256.6 (-388.3, -119.0)	101.7 (23.1, 200.9)	-358.4 (-471.6, -262.1)	4.15 (-3.33, 10.37)	-1.95 (-3.14, -0.45)
Strasbourg	-258.9 (-314.0, -206.6)	151.1 (115.4, 187.4)	-410.0 (-455.0, -366.9)	9.74 (7.52, 11.71)	-6.66 (-7.95, -5.07)
Leeds	-262.9 (-287.4, -238.3)	18.1 (11.8, 24.5)	-281.1 (-305.6, -255.8)	2.36 (1.59, 3.12)	-7.56 (-8.56, -6.56)
Valencia	-268.6 (-340.0, -193.2)	203.5 (160.7, 247.0)	-472.2 (-532.9, -406.9)	7.5 (4.92, 9.9)	-11.03 (-12.26, -9.8)
Dublin	-269.1 (-290.2, -247.1)	10.4 (5.5, 18.7)	-279.5 (-302.2, -259.6)	1.56 (0.59, 2.4)	-2.69 (-2.89, -2.5)
Edinburgh	-277.7 (-317.7, -233.0)	22.7 (17.1, 34.2)	-300.5 (-341.7, -260.1)	3.06 (2.15, 3.87)	-4.11 (-4.81, -3.39)
Barcelona	-330.3 (-415.1, -242.1)	245.2 (200.0, 297.7)	-575.6 (-656.4, -493.3)	11.54 (9.28, 13.42)	-6.01 (-7.06, -4.95)
Lisbon	-360.7 (-401.7, -317.5)	79.3 (68.7, 93.4)	-440.1 (-482.2, -400.5)	3.97 (3.48, 4.37)	-4.3 (-5.03, -3.51)
London	-442.0 (-514.8, -357.7)	91.8 (65.9, 118.2)	-534.0 (-603.4, -453.2)	10.63 (8.16, 12.84)	-8.92 (-10.65, -7.12)
Porto	-476.1 (-528.0, -418.9)	115.8 (99.4, 136.3)	-592.0 (-642.6, -544.5)	5.98 (4.95, 6.89)	-5.72 (-6.71, -4.65)

Table S5: Economic impact of mortality associated with UHI, in 2021-EUR per urban adult inhabitant, local population age structure. Values in brackets indicate the 90% confidence interval bounds. (continued)

City	Net [EUR per year]	Heat [EUR per year]	Cold [EUR per year]	Heat extreme [EUR per day]	Cold extreme [EUR per day]
Glasgow	-624.4 (-699.1, -538.3)	43.7 (29.8, 60.4)	-668.2 (-744.4, -584.1)	5.49 (3.76, 6.97)	-9.3 (-11.08, -7.29)

Table S6: Years of life lost associated with UHI, per 100,000 urban adult population, local population age structure. Values in brackets indicate the 90% confidence interval bounds.

City	Net [per year]	Heat [per year]	Cold [per year]	Heat extreme [per day]	Cold extreme [per day]
Trieste	60.1 (31.1, 90.1)	159.6 (135.2, 180.6)	-99.6 (-118.2, -78.1)	3.97 (2.91, 4.93)	-0.41 (-0.76, -0.03)
Genoa	55.9 (15.5, 95.3)	171.8 (138.5, 194.4)	-115.9 (-139.6, -80.0)	3.41 (2.49, 4.23)	-0.91 (-1.16, -0.66)
Bologna	41.5 (18.4, 66.4)	155.2 (140.1, 169.1)	-113.6 (-133.4, -93.3)	3.29 (2.6, 3.92)	0.05 (-0.54, 0.65)
Thessaloniki	39.4 (0.9, 75.9)	166.6 (133.0, 197.1)	-127.3 (-152.5, -99.5)	3.8 (2.21, 5.03)	-0.67 (-0.88, -0.47)
Ljubljana	32.5 (6.0, 59.6)	93.0 (73.5, 114.6)	-60.5 (-81.2, -42.3)	2.3 (1.2, 3.23)	0.1 (-0.3, 0.51)
Madrid	27.0 (-2.7, 55.4)	83.1 (65.1, 104.6)	-56.2 (-81.9, -35.2)	3.39 (2.32, 4.25)	-0.8 (-1.36, -0.25)
Graz	25.9 (-1.5, 50.3)	64.5 (50.6, 90.7)	-38.5 (-68.0, -24.3)	3.29 (2.16, 4.11)	0.38 (-0.1, 0.89)
Vienna	25.8 (-1.3, 49.4)	89.4 (69.5, 110.5)	-63.6 (-84.7, -46.5)	3.87 (2.75, 4.7)	-0.16 (-0.57, 0.28)
Zagreb	25.0 (-4.3, 53.2)	97.7 (73.5, 117.9)	-72.7 (-94.8, -49.7)	1.73 (0.55, 2.73)	-0.09 (-0.41, 0.24)
Bucharest	20.2 (-25.2, 58.8)	167.1 (132.4, 198.9)	-146.9 (-177.8, -116.3)	6.33 (4.85, 7.73)	-0.14 (-0.6, 0.4)
Basel	15.8 (-1.2, 35.0)	82.3 (70.4, 93.1)	-66.5 (-79.8, -50.9)	4.07 (3.49, 4.54)	0.16 (-0.28, 0.65)
Geneva	13.1 (-0.1, 27.1)	54.4 (44.4, 63.6)	-41.3 (-50.7, -30.3)	2.34 (1.89, 2.7)	0.06 (-0.17, 0.32)
Luxembourg	11.1 (-2.0, 24.5)	42.5 (35.3, 52.4)	-31.4 (-42.6, -21.4)	2.4 (2.0, 2.72)	0.59 (0.14, 1.09)
Milan	7.7 (-17.2, 33.4)	144.9 (130.1, 161.3)	-137.2 (-157.8, -115.9)	4.98 (4.12, 5.8)	-1.39 (-1.96, -0.83)
Debrecen	7.1 (-31.2, 38.1)	92.6 (59.2, 120.5)	-85.6 (-105.0, -67.2)	2.14 (0.39, 3.56)	-0.1 (-0.38, 0.2)
Munich	7.0 (-10.1, 26.7)	46.5 (33.3, 59.5)	-39.4 (-53.3, -24.6)	3.0 (2.17, 3.68)	-0.06 (-0.41, 0.31)
Turin	6.7 (-30.0, 43.9)	244.2 (218.4, 269.2)	-237.5 (-265.9, -208.2)	5.23 (4.04, 6.29)	-1.48 (-2.17, -0.79)
Frankfurt am Main	4.5 (-7.0, 17.3)	46.3 (40.1, 53.1)	-41.8 (-52.2, -27.4)	2.75 (2.38, 3.04)	0.63 (0.36, 0.91)
Liege	1.8 (-15.9, 21.0)	66.4 (55.8, 80.9)	-64.6 (-80.4, -51.5)	3.92 (3.25, 4.46)	-0.09 (-0.43, 0.29)
Toulouse	1.3 (-19.5, 22.8)	71.3 (58.7, 85.0)	-70.0 (-87.6, -54.5)	3.56 (2.81, 4.2)	-1.43 (-1.84, -0.94)
Zurich	-0.4 (-17.9, 19.1)	55.3 (43.1, 67.9)	-55.7 (-70.4, -40.4)	2.56 (2.02, 3.05)	0.45 (0.08, 0.82)
Charleroi	-1.5 (-21.4, 18.6)	64.2 (51.2, 79.2)	-65.7 (-82.6, -51.0)	3.53 (2.75, 4.13)	-0.35 (-0.83, 0.23)

Table S6: Years of life lost associated with UHI, per 100,000 urban adult population, local population age structure. Values in brackets indicate the 90% confidence interval bounds. (continued)

City	Net [per year]	Heat [per year]	Cold [per year]	Heat extreme [per day]	Cold extreme [per day]
Marseille	-2.6 (-35.1, 31.9)	94.1 (75.5, 112.4)	-96.7 (-124.1, -68.6)	4.56 (3.6, 5.41)	-1.66 (-2.07, -1.21)
Cologne	-2.8 (-14.1, 8.6)	55.6 (49.3, 62.9)	-58.4 (-67.4, -49.2)	3.2 (2.83, 3.52)	0.58 (0.2, 0.99)
Kosice	-3.2 (-48.6, 33.6)	104.9 (63.8, 135.7)	-108.0 (-131.1, -84.4)	2.63 (0.74, 4.24)	-0.02 (-0.33, 0.31)
Dusseldorf	-5.9 (-20.2, 8.8)	85.6 (77.5, 95.3)	-91.5 (-103.4, -80.9)	4.56 (4.1, 4.97)	0.82 (0.3, 1.4)
Palermo	-7.0 (-26.5, 11.9)	96.4 (80.5, 103.6)	-103.4 (-116.6, -82.7)	1.69 (1.12, 2.16)	-0.59 (-0.67, -0.5)
Pecs	-9.2 (-55.1, 32.4)	106.7 (68.9, 143.7)	-116.0 (-143.6, -86.0)	3.04 (1.43, 4.45)	-0.06 (-0.53, 0.52)
Split	-9.7 (-43.0, 20.4)	106.1 (81.2, 127.7)	-115.8 (-141.7, -89.3)	2.12 (0.92, 3.12)	-0.63 (-0.87, -0.39)
Bilbao	-10.6 (-42.9, 23.6)	83.6 (65.5, 105.0)	-94.2 (-118.5, -71.4)	3.38 (2.54, 4.11)	-0.85 (-1.02, -0.68)
Varna	-15.5 (-65.6, 30.3)	127.4 (82.7, 168.3)	-143.0 (-171.1, -112.8)	3.18 (1.92, 4.43)	-0.35 (-0.58, -0.1)
Murcia	-16.7 (-35.4, 1.5)	61.3 (47.7, 75.0)	-78.1 (-91.9, -63.5)	1.65 (0.95, 2.24)	-1.12 (-1.32, -0.92)
Warsaw	-18.1 (-45.4, 9.6)	60.5 (44.5, 81.4)	-78.6 (-101.2, -59.1)	3.96 (2.73, 4.99)	-0.07 (-0.4, 0.29)
Berlin	-18.2 (-32.9, -3.5)	53.0 (46.3, 60.9)	-71.2 (-85.2, -58.3)	3.78 (3.3, 4.15)	0.15 (-0.17, 0.45)
Stockholm	-19.3 (-22.7, -15.4)	3.4 (2.2, 4.7)	-22.7 (-26.0, -18.7)	0.43 (0.28, 0.58)	-0.05 (-0.08, -0.02)
Nice	-19.6 (-37.6, -2.1)	53.3 (43.7, 62.3)	-72.9 (-88.5, -57.1)	2.11 (1.69, 2.46)	-0.59 (-0.69, -0.48)
Brussels	-20.9 (-37.0, -4.5)	49.0 (41.3, 59.6)	-69.9 (-83.6, -58.6)	3.34 (2.76, 3.79)	-0.67 (-1.1, -0.15)
Bratislava	-21.7 (-53.3, 7.1)	58.0 (35.9, 80.8)	-79.7 (-104.9, -58.3)	2.55 (1.43, 3.49)	0.07 (-0.36, 0.53)
Krakow	-23.8 (-56.3, 6.0)	86.9 (62.1, 110.8)	-110.7 (-132.7, -90.6)	3.48 (2.28, 4.55)	-0.1 (-0.3, 0.13)
Budapest	-25.3 (-64.6, 12.7)	104.4 (75.2, 136.3)	-129.7 (-160.1, -102.4)	4.65 (3.18, 5.98)	-0.2 (-0.55, 0.21)
Miskolc	-25.3 (-98.6, 36.3)	173.4 (108.7, 228.0)	-198.7 (-242.5, -153.3)	3.21 (0.46, 5.43)	0.01 (-0.82, 0.91)
Sofia	-25.9 (-65.8, 11.7)	88.7 (58.6, 119.8)	-114.6 (-145.8, -85.9)	3.27 (2.04, 4.35)	0.03 (-0.13, 0.21)
Antwerp	-26.3 (-46.8, -7.2)	49.7 (40.4, 61.4)	-75.9 (-92.9, -61.3)	3.52 (2.81, 4.12)	-0.65 (-1.17, -0.13)
Vilnius	-26.8 (-72.6, 18.2)	29.2 (-9.1, 69.8)	-56.0 (-93.1, -25.9)	0.58 (-3.15, 3.54)	0.18 (-0.33, 0.82)

Table S6: Years of life lost associated with UHI, per 100,000 urban adult population, local population age structure. Values in brackets indicate the 90% confidence interval bounds. (continued)

City	Net [per year]	Heat [per year]	Cold [per year]	Heat extreme [per day]	Cold extreme [per day]
Cluj-Napoca	-27.9 (-76.8, 15.5)	127.8 (88.1, 162.1)	-155.7 (-185.3, -125.3)	2.08 (0.54, 3.68)	-0.26 (-0.6, 0.15)
Ghent	-28.5 (-48.7, -7.2)	42.4 (33.8, 54.5)	-70.9 (-88.3, -54.6)	3.11 (2.42, 3.7)	-0.67 (-1.33, 0.08)
Leipzig	-28.6 (-46.6, -10.4)	78.7 (67.2, 91.3)	-107.3 (-123.2, -92.4)	3.04 (2.47, 3.55)	-0.05 (-0.49, 0.43)
Lille	-30.9 (-52.9, -9.1)	54.0 (45.5, 66.9)	-84.9 (-107.1, -67.6)	3.12 (2.4, 3.75)	-1.07 (-1.84, -0.13)
Naples	-33.8 (-52.7, -14.8)	64.3 (52.9, 76.3)	-98.1 (-115.4, -81.4)	2.16 (1.49, 2.75)	-0.88 (-1.05, -0.7)
Gyor	-36.0 (-82.5, 6.8)	95.3 (60.6, 134.6)	-131.2 (-163.2, -104.1)	3.28 (1.61, 4.82)	-0.45 (-0.98, 0.21)
Amsterdam	-36.1 (-64.7, -5.2)	40.5 (28.9, 59.6)	-76.6 (-102.2, -55.5)	3.0 (2.02, 3.84)	-1.13 (-1.68, -0.44)
Hamburg	-37.7 (-51.7, -22.7)	30.8 (24.2, 39.0)	-68.5 (-81.8, -56.6)	2.75 (2.11, 3.37)	-0.92 (-1.18, -0.62)
Riga	-38.0 (-66.5, -9.2)	21.7 (2.7, 43.3)	-59.7 (-82.2, -36.7)	0.42 (-1.29, 1.9)	-0.17 (-0.37, 0.08)
Nantes	-38.3 (-60.5, -16.4)	66.3 (55.8, 76.2)	-104.6 (-123.4, -86.8)	3.54 (2.89, 4.04)	-1.12 (-1.85, -0.3)
Helsinki	-39.4 (-47.9, -30.1)	11.7 (6.5, 16.9)	-51.1 (-59.5, -42.3)	1.35 (0.77, 1.88)	-0.24 (-0.34, -0.15)
Prague	-42.5 (-82.4, -3.6)	55.3 (36.6, 80.0)	-97.8 (-137.7, -65.9)	3.28 (2.01, 4.38)	0.3 (-0.36, 0.96)
Utrecht	-42.8 (-68.2, -14.7)	50.3 (38.7, 67.7)	-93.1 (-116.5, -74.1)	3.19 (2.3, 3.88)	-1.27 (-1.92, -0.54)
Paris	-43.3 (-83.8, 0.9)	73.2 (58.6, 92.2)	-116.5 (-155.0, -78.9)	5.05 (4.2, 5.73)	-1.81 (-2.8, -0.79)
Rotterdam	-43.9 (-69.8, -18.1)	48.3 (36.8, 65.8)	-92.1 (-115.7, -73.7)	3.17 (2.35, 3.84)	-0.9 (-1.51, -0.17)
Rome	-46.6 (-65.6, -27.1)	114.4 (101.9, 127.3)	-161.0 (-177.2, -144.6)	2.95 (2.36, 3.48)	-0.95 (-1.21, -0.66)
Gdansk	-47.3 (-73.6, -19.4)	30.1 (18.5, 47.8)	-77.4 (-101.3, -56.9)	2.06 (1.12, 2.83)	-0.41 (-0.77, -0.01)
Tallinn	-48.3 (-67.0, -28.3)	19.2 (8.9, 32.9)	-67.5 (-86.5, -52.5)	1.68 (0.34, 2.84)	-0.19 (-0.36, 0.01)
Oslo	-49.3 (-65.4, -31.6)	0.8 (-8.5, 5.7)	-50.2 (-67.6, -27.9)	0.63 (0.13, 1.07)	0.15 (-0.05, 0.36)
Wroclaw	-50.1 (-76.6, -23.0)	57.5 (41.1, 77.1)	-107.6 (-130.0, -89.5)	2.34 (1.57, 3.07)	-0.3 (-0.66, 0.13)
Montpellier	-52.0 (-74.6, -30.0)	83.4 (71.7, 96.7)	-135.4 (-154.9, -117.7)	4.21 (3.51, 4.82)	-0.86 (-1.4, -0.24)
Athens	-53.0 (-91.4, -13.9)	127.9 (104.4, 153.0)	-181.0 (-214.8, -150.0)	4.71 (3.82, 5.54)	-0.53 (-0.82, -0.21)

Table S6: Years of life lost associated with UHI, per 100,000 urban adult population, local population age structure. Values in brackets indicate the 90% confidence interval bounds. (continued)

City	Net [per year]	Heat [per year]	Cold [per year]	Heat extreme [per day]	Cold extreme [per day]
Bari	-54.3 (-69.0, -39.4)	37.9 (32.4, 43.6)	-92.2 (-107.0, -77.7)	0.9 (0.64, 1.12)	-0.79 (-1.0, -0.57)
Lyon	-54.3 (-86.0, -24.1)	94.7 (72.7, 118.2)	-149.0 (-173.8, -126.3)	4.54 (3.37, 5.56)	-2.73 (-3.13, -2.27)
Sevilla	-59.3 (-93.0, -29.4)	97.6 (75.5, 118.6)	-156.9 (-181.7, -132.2)	2.06 (0.86, 3.16)	-3.44 (-4.07, -2.79)
Malaga	-60.9 (-85.8, -36.2)	49.6 (40.8, 59.6)	-110.5 (-134.3, -86.8)	1.66 (1.16, 2.12)	-1.38 (-1.62, -1.14)
Szeged	-64.0 (-111.9, -19.0)	98.6 (60.9, 135.6)	-162.6 (-195.1, -129.4)	2.43 (0.96, 3.8)	-1.1 (-1.71, -0.37)
Bordeaux	-65.2 (-85.2, -45.3)	63.3 (50.5, 75.7)	-128.5 (-145.7, -111.6)	4.18 (3.32, 4.88)	-2.18 (-2.63, -1.67)
Copenhagen	-73.3 (-85.0, -61.1)	6.3 (3.7, 11.1)	-79.6 (-91.3, -69.3)	0.84 (0.45, 1.18)	-1.15 (-1.49, -0.79)
Brasov	-75.5 (-136.6, -17.3)	63.2 (17.4, 113.5)	-138.8 (-177.0, -106.2)	1.79 (-0.63, 4.1)	-0.34 (-0.65, 0.0)
Leeds	-76.3 (-85.6, -67.2)	4.7 (2.5, 6.8)	-81.0 (-90.4, -71.7)	0.62 (0.31, 0.9)	-2.19 (-2.58, -1.82)
Strasbourg	-76.8 (-97.2, -57.8)	47.3 (35.0, 61.1)	-124.1 (-140.8, -109.4)	3.07 (2.21, 3.8)	-1.82 (-2.37, -1.15)
Klaipeda	-78.8 (-126.0, -28.6)	31.0 (2.9, 66.5)	-109.8 (-150.8, -74.9)	0.94 (-1.58, 3.19)	-0.47 (-0.94, 0.11)
Valencia	-81.1 (-106.2, -55.1)	66.7 (52.0, 82.0)	-147.8 (-170.0, -126.0)	2.28 (1.37, 3.14)	-3.5 (-3.94, -3.05)
Edinburgh	-81.1 (-95.5, -65.9)	6.4 (4.5, 10.4)	-87.6 (-102.7, -73.7)	0.89 (0.54, 1.18)	-1.13 (-1.4, -0.86)
Dublin	-90.7 (-98.8, -82.1)	2.5 (0.9, 5.4)	-93.2 (-101.8, -85.4)	0.38 (0.01, 0.71)	-0.9 (-0.98, -0.83)
Barcelona	-100.7 (-129.7, -69.3)	81.3 (65.4, 100.7)	-182.0 (-209.2, -154.5)	3.67 (2.88, 4.34)	-1.79 (-2.16, -1.41)
Lisbon	-115.1 (-130.1, -99.0)	28.9 (24.4, 34.4)	-144.0 (-158.8, -130.0)	1.33 (1.15, 1.48)	-1.29 (-1.55, -1.01)
Porto	-149.8 (-169.5, -129.3)	39.7 (33.1, 48.5)	-189.5 (-207.4, -172.8)	1.93 (1.53, 2.26)	-1.68 (-2.05, -1.28)
London	-152.5 (-182.5, -118.3)	32.7 (22.8, 43.0)	-185.3 (-214.1, -153.4)	3.86 (2.83, 4.75)	-3.15 (-3.86, -2.42)
Glasgow	-183.5 (-212.5, -151.6)	12.5 (8.1, 18.6)	-195.9 (-224.7, -164.6)	1.59 (0.96, 2.13)	-2.59 (-3.27, -1.87)

Table S7: Economic impact of years of life lost associated with UHI, in 2021-EUR per urban adult inhabitant, local population age structure. Values in brackets indicate the 90% confidence interval bounds.

City	Net [EUR per year]	Heat [EUR per year]	Cold [EUR per year]	Heat extreme [EUR per day]	Cold extreme [EUR per day]
Trieste	27.6 (14.3, 41.4)	73.4 (62.2, 83.1)	-45.8 (-54.4, -35.9)	1.83 (1.34, 2.27)	-0.19 (-0.35, -0.02)
Genoa	25.7 (7.1, 43.9)	79.0 (63.7, 89.4)	-53.3 (-64.2, -36.8)	1.57 (1.14, 1.95)	-0.42 (-0.53, -0.3)
Bologna	19.1 (8.4, 30.5)	71.4 (64.4, 77.8)	-52.3 (-61.4, -42.9)	1.51 (1.2, 1.8)	0.02 (-0.25, 0.3)
Thessaloniki	18.1 (0.4, 34.9)	76.7 (61.2, 90.7)	-58.5 (-70.2, -45.8)	1.75 (1.02, 2.31)	-0.31 (-0.4, -0.22)
Ljubljana	14.9 (2.8, 27.4)	42.8 (33.8, 52.7)	-27.8 (-37.3, -19.4)	1.06 (0.55, 1.48)	0.05 (-0.14, 0.23)
Madrid	12.4 (-1.2, 25.5)	38.2 (30.0, 48.1)	-25.8 (-37.7, -16.2)	1.56 (1.07, 1.96)	-0.37 (-0.62, -0.12)
Graz	11.9 (-0.7, 23.1)	29.7 (23.3, 41.7)	-17.7 (-31.3, -11.2)	1.51 (0.99, 1.89)	0.17 (-0.05, 0.41)
Vienna	11.8 (-0.6, 22.7)	41.1 (32.0, 50.8)	-29.3 (-39.0, -21.4)	1.78 (1.27, 2.16)	-0.07 (-0.26, 0.13)
Zagreb	11.5 (-2.0, 24.5)	44.9 (33.8, 54.3)	-33.4 (-43.6, -22.9)	0.8 (0.25, 1.26)	-0.04 (-0.19, 0.11)
Bucharest	9.3 (-11.6, 27.1)	76.9 (60.9, 91.5)	-67.6 (-81.8, -53.5)	2.91 (2.23, 3.55)	-0.06 (-0.27, 0.18)
Basel	7.3 (-0.6, 16.1)	37.9 (32.4, 42.8)	-30.6 (-36.7, -23.4)	1.87 (1.61, 2.09)	0.07 (-0.13, 0.3)
Geneva	6.0 (-0.0, 12.5)	25.0 (20.4, 29.2)	-19.0 (-23.3, -14.0)	1.08 (0.87, 1.24)	0.03 (-0.08, 0.15)
Luxembourg	5.1 (-0.9, 11.3)	19.6 (16.3, 24.1)	-14.5 (-19.6, -9.8)	1.11 (0.92, 1.25)	0.27 (0.06, 0.5)
Milan	3.5 (-7.9, 15.4)	66.6 (59.8, 74.2)	-63.1 (-72.6, -53.3)	2.29 (1.89, 2.67)	-0.64 (-0.9, -0.38)
Debrecen	3.2 (-14.4, 17.5)	42.6 (27.2, 55.4)	-39.4 (-48.3, -30.9)	0.99 (0.18, 1.64)	-0.05 (-0.17, 0.09)
Munich	3.2 (-4.6, 12.3)	21.4 (15.3, 27.4)	-18.1 (-24.5, -11.3)	1.38 (1.0, 1.69)	-0.03 (-0.19, 0.14)
Turin	3.1 (-13.8, 20.2)	112.3 (100.5, 123.8)	-109.3 (-122.3, -95.8)	2.41 (1.86, 2.89)	-0.68 (-1.0, -0.36)
Frankfurt am Main	2.1 (-3.2, 8.0)	21.3 (18.4, 24.4)	-19.2 (-24.0, -12.6)	1.26 (1.09, 1.4)	0.29 (0.17, 0.42)
Liege	0.8 (-7.3, 9.7)	30.5 (25.7, 37.2)	-29.7 (-37.0, -23.7)	1.8 (1.49, 2.05)	-0.04 (-0.2, 0.13)
Toulouse	0.6 (-9.0, 10.5)	32.8 (27.0, 39.1)	-32.2 (-40.3, -25.1)	1.64 (1.29, 1.93)	-0.66 (-0.85, -0.43)
Zurich	-0.2 (-8.2, 8.8)	25.4 (19.8, 31.2)	-25.6 (-32.4, -18.6)	1.18 (0.93, 1.4)	0.21 (0.04, 0.38)

Table S7: Economic impact of years of life lost associated with UHI, in 2021-EUR per urban adult inhabitant, local population age structure. Values in brackets indicate the 90% confidence interval bounds. (continued)

City	Net [EUR per year]	Heat [EUR per year]	Cold [EUR per year]	Heat extreme [EUR per day]	Cold extreme [EUR per day]
Charleroi	-0.7 (-9.8, 8.6)	29.5 (23.5, 36.4)	-30.2 (-38.0, -23.4)	1.63 (1.26, 1.9)	-0.16 (-0.38, 0.11)
Marseille	-1.2 (-16.1, 14.7)	43.3 (34.7, 51.7)	-44.5 (-57.1, -31.5)	2.1 (1.66, 2.49)	-0.77 (-0.95, -0.56)
Cologne	-1.3 (-6.5, 3.9)	25.6 (22.7, 28.9)	-26.9 (-31.0, -22.6)	1.47 (1.3, 1.62)	0.27 (0.09, 0.46)
Kosice	-1.4 (-22.3, 15.4)	48.2 (29.3, 62.4)	-49.7 (-60.3, -38.8)	1.21 (0.34, 1.95)	-0.01 (-0.15, 0.14)
Dusseldorf	-2.7 (-9.3, 4.1)	39.4 (35.6, 43.9)	-42.1 (-47.5, -37.2)	2.1 (1.88, 2.28)	0.38 (0.14, 0.65)
Palermo	-3.2 (-12.2, 5.5)	44.3 (37.0, 47.6)	-47.6 (-53.6, -38.0)	0.78 (0.52, 0.99)	-0.27 (-0.31, -0.23)
Pecs	-4.3 (-25.3, 14.9)	49.1 (31.7, 66.1)	-53.3 (-66.0, -39.6)	1.4 (0.66, 2.05)	-0.03 (-0.25, 0.24)
Split	-4.5 (-19.8, 9.4)	48.8 (37.4, 58.8)	-53.3 (-65.2, -41.1)	0.98 (0.42, 1.44)	-0.29 (-0.4, -0.18)
Bilbao	-4.9 (-19.7, 10.9)	38.4 (30.1, 48.3)	-43.3 (-54.5, -32.8)	1.56 (1.17, 1.89)	-0.39 (-0.47, -0.31)
Varna	-7.2 (-30.2, 13.9)	58.6 (38.0, 77.4)	-65.8 (-78.7, -51.9)	1.46 (0.89, 2.04)	-0.16 (-0.27, -0.04)
Murcia	-7.7 (-16.3, 0.7)	28.2 (21.9, 34.5)	-35.9 (-42.3, -29.2)	0.76 (0.44, 1.03)	-0.52 (-0.61, -0.42)
Warsaw	-8.3 (-20.9, 4.4)	27.8 (20.5, 37.5)	-36.2 (-46.6, -27.2)	1.82 (1.25, 2.29)	-0.03 (-0.18, 0.13)
Berlin	-8.4 (-15.1, -1.6)	24.4 (21.3, 28.0)	-32.8 (-39.2, -26.8)	1.74 (1.52, 1.91)	0.07 (-0.08, 0.21)
Stockholm	-8.9 (-10.4, -7.1)	1.6 (1.0, 2.1)	-10.4 (-12.0, -8.6)	0.2 (0.13, 0.27)	-0.02 (-0.03, -0.01)
Nice	-9.0 (-17.3, -1.0)	24.5 (20.1, 28.7)	-33.5 (-40.7, -26.3)	0.97 (0.78, 1.13)	-0.27 (-0.32, -0.22)
Brussels	-9.6 (-17.0, -2.1)	22.5 (19.0, 27.4)	-32.2 (-38.4, -27.0)	1.54 (1.27, 1.74)	-0.31 (-0.51, -0.07)
Bratislava	-10.0 (-24.5, 3.3)	26.7 (16.5, 37.2)	-36.7 (-48.3, -26.8)	1.17 (0.66, 1.61)	0.03 (-0.17, 0.24)
Krakow	-10.9 (-25.9, 2.8)	40.0 (28.6, 51.0)	-50.9 (-61.0, -41.7)	1.6 (1.05, 2.09)	-0.05 (-0.14, 0.06)
Budapest	-11.6 (-29.7, 5.8)	48.0 (34.6, 62.7)	-59.7 (-73.6, -47.1)	2.14 (1.46, 2.75)	-0.09 (-0.25, 0.1)
Miskolc	-11.6 (-45.4, 16.7)	79.8 (50.0, 104.9)	-91.4 (-111.6, -70.5)	1.48 (0.21, 2.5)	0.0 (-0.38, 0.42)
Sofia	-11.9 (-30.3, 5.4)	40.8 (26.9, 55.1)	-52.7 (-67.1, -39.5)	1.51 (0.94, 2.0)	0.02 (-0.06, 0.1)

Table S7: Economic impact of years of life lost associated with UHI, in 2021-EUR per urban adult inhabitant, local population age structure. Values in brackets indicate the 90% confidence interval bounds. (continued)

City	Net [EUR per year]	Heat [EUR per year]	Cold [EUR per year]	Heat extreme [EUR per day]	Cold extreme [EUR per day]
Antwerp	-12.1 (-21.5, -3.3)	22.9 (18.6, 28.2)	-34.9 (-42.7, -28.2)	1.62 (1.29, 1.9)	-0.3 (-0.54, -0.06)
Vilnius	-12.3 (-33.4, 8.4)	13.4 (-4.2, 32.1)	-25.7 (-42.8, -11.9)	0.27 (-1.45, 1.63)	0.08 (-0.15, 0.38)
Cluj-Napoca	-12.8 (-35.3, 7.1)	58.8 (40.5, 74.6)	-71.6 (-85.2, -57.6)	0.95 (0.25, 1.69)	-0.12 (-0.28, 0.07)
Ghent	-13.1 (-22.4, -3.3)	19.5 (15.5, 25.1)	-32.6 (-40.6, -25.1)	1.43 (1.11, 1.7)	-0.31 (-0.61, 0.04)
Leipzig	-13.2 (-21.4, -4.8)	36.2 (30.9, 42.0)	-49.4 (-56.7, -42.5)	1.4 (1.14, 1.63)	-0.03 (-0.22, 0.2)
Lille	-14.2 (-24.3, -4.2)	24.8 (20.9, 30.8)	-39.1 (-49.2, -31.1)	1.44 (1.1, 1.73)	-0.49 (-0.85, -0.06)
Naples	-15.5 (-24.3, -6.8)	29.6 (24.3, 35.1)	-45.1 (-53.1, -37.4)	0.99 (0.69, 1.26)	-0.41 (-0.48, -0.32)
Gyor	-16.5 (-38.0, 3.1)	43.8 (27.9, 61.9)	-60.4 (-75.1, -47.9)	1.51 (0.74, 2.22)	-0.21 (-0.45, 0.1)
Amsterdam	-16.6 (-29.8, -2.4)	18.6 (13.3, 27.4)	-35.2 (-47.0, -25.5)	1.38 (0.93, 1.77)	-0.52 (-0.77, -0.2)
Hamburg	-17.3 (-23.8, -10.5)	14.2 (11.1, 17.9)	-31.5 (-37.6, -26.0)	1.27 (0.97, 1.55)	-0.42 (-0.54, -0.29)
Riga	-17.5 (-30.6, -4.2)	10.0 (1.2, 19.9)	-27.5 (-37.8, -16.9)	0.19 (-0.59, 0.87)	-0.08 (-0.17, 0.04)
Nantes	-17.6 (-27.8, -7.6)	30.5 (25.7, 35.1)	-48.1 (-56.8, -39.9)	1.63 (1.33, 1.86)	-0.52 (-0.85, -0.14)
Helsinki	-18.1 (-22.0, -13.8)	5.4 (3.0, 7.8)	-23.5 (-27.4, -19.5)	0.62 (0.35, 0.87)	-0.11 (-0.16, -0.07)
Prague	-19.6 (-37.9, -1.6)	25.4 (16.8, 36.8)	-45.0 (-63.3, -30.3)	1.51 (0.93, 2.02)	0.14 (-0.17, 0.44)
Utrecht	-19.7 (-31.4, -6.8)	23.1 (17.8, 31.2)	-42.8 (-53.6, -34.1)	1.47 (1.06, 1.79)	-0.58 (-0.88, -0.25)
Paris	-19.9 (-38.6, 0.4)	33.7 (27.0, 42.4)	-53.6 (-71.3, -36.3)	2.32 (1.93, 2.63)	-0.83 (-1.29, -0.36)
Rotterdam	-20.2 (-32.1, -8.3)	22.2 (16.9, 30.3)	-42.4 (-53.2, -33.9)	1.46 (1.08, 1.77)	-0.41 (-0.69, -0.08)
Rome	-21.4 (-30.2, -12.5)	52.6 (46.9, 58.6)	-74.0 (-81.5, -66.5)	1.36 (1.08, 1.6)	-0.44 (-0.56, -0.31)
Gdansk	-21.8 (-33.9, -8.9)	13.8 (8.5, 22.0)	-35.6 (-46.6, -26.2)	0.95 (0.52, 1.3)	-0.19 (-0.36, -0.0)
Tallinn	-22.2 (-30.8, -13.0)	8.8 (4.1, 15.2)	-31.1 (-39.8, -24.2)	0.77 (0.16, 1.31)	-0.09 (-0.17, 0.0)
Oslo	-22.7 (-30.1, -14.5)	0.4 (-3.9, 2.6)	-23.1 (-31.1, -12.9)	0.29 (0.06, 0.49)	0.07 (-0.02, 0.17)

Table S7: Economic impact of years of life lost associated with UHI, in 2021-EUR per urban adult inhabitant, local population age structure. Values in brackets indicate the 90% confidence interval bounds. (continued)

City	Net [EUR per year]	Heat [EUR per year]	Cold [EUR per year]	Heat extreme [EUR per day]	Cold extreme [EUR per day]
Wroclaw	-23.1 (-35.2, -10.6)	26.4 (18.9, 35.5)	-49.5 (-59.8, -41.2)	1.08 (0.72, 1.41)	-0.14 (-0.3, 0.06)
Montpellier	-23.9 (-34.3, -13.8)	38.3 (33.0, 44.5)	-62.3 (-71.2, -54.1)	1.93 (1.61, 2.22)	-0.4 (-0.64, -0.11)
Athens	-24.4 (-42.0, -6.4)	58.8 (48.0, 70.4)	-83.2 (-98.8, -69.0)	2.17 (1.76, 2.55)	-0.24 (-0.38, -0.1)
Bari	-25.0 (-31.8, -18.1)	17.5 (14.9, 20.0)	-42.4 (-49.2, -35.7)	0.41 (0.29, 0.52)	-0.36 (-0.46, -0.26)
Lyon	-25.0 (-39.6, -11.1)	43.6 (33.4, 54.4)	-68.6 (-79.9, -58.1)	2.09 (1.55, 2.56)	-1.26 (-1.44, -1.04)
Sevilla	-27.3 (-42.8, -13.5)	44.9 (34.7, 54.5)	-72.2 (-83.6, -60.8)	0.95 (0.4, 1.45)	-1.58 (-1.87, -1.29)
Malaga	-28.0 (-39.5, -16.6)	22.8 (18.8, 27.4)	-50.8 (-61.8, -39.9)	0.76 (0.54, 0.98)	-0.64 (-0.74, -0.53)
Szeged	-29.4 (-51.5, -8.7)	45.4 (28.0, 62.4)	-74.8 (-89.8, -59.5)	1.12 (0.44, 1.75)	-0.51 (-0.78, -0.17)
Bordeaux	-30.0 (-39.2, -20.8)	29.1 (23.2, 34.8)	-59.1 (-67.0, -51.3)	1.92 (1.53, 2.25)	-1.0 (-1.21, -0.77)
Copenhagen	-33.7 (-39.1, -28.1)	2.9 (1.7, 5.1)	-36.6 (-42.0, -31.9)	0.39 (0.21, 0.54)	-0.53 (-0.68, -0.36)
Brasov	-34.7 (-62.8, -8.0)	29.1 (8.0, 52.2)	-63.8 (-81.4, -48.8)	0.82 (-0.29, 1.89)	-0.16 (-0.3, 0.0)
Leeds	-35.1 (-39.4, -30.9)	2.1 (1.2, 3.1)	-37.2 (-41.6, -33.0)	0.28 (0.14, 0.41)	-1.01 (-1.19, -0.84)
Strasbourg	-35.3 (-44.7, -26.6)	21.8 (16.1, 28.1)	-57.1 (-64.8, -50.3)	1.41 (1.02, 1.75)	-0.84 (-1.09, -0.53)
Klaipeda	-36.2 (-58.0, -13.1)	14.3 (1.3, 30.6)	-50.5 (-69.4, -34.5)	0.43 (-0.73, 1.47)	-0.22 (-0.43, 0.05)
Valencia	-37.3 (-48.8, -25.4)	30.7 (23.9, 37.7)	-68.0 (-78.2, -58.0)	1.05 (0.63, 1.44)	-1.61 (-1.81, -1.4)
Edinburgh	-37.3 (-43.9, -30.3)	3.0 (2.1, 4.8)	-40.3 (-47.3, -33.9)	0.41 (0.25, 0.54)	-0.52 (-0.64, -0.39)
Dublin	-41.7 (-45.4, -37.8)	1.2 (0.4, 2.5)	-42.9 (-46.8, -39.3)	0.18 (0.01, 0.33)	-0.41 (-0.45, -0.38)
Barcelona	-46.3 (-59.7, -31.9)	37.4 (30.1, 46.3)	-83.7 (-96.3, -71.1)	1.69 (1.33, 2.0)	-0.82 (-0.99, -0.65)
Lisbon	-52.9 (-59.8, -45.5)	13.3 (11.2, 15.8)	-66.2 (-73.0, -59.8)	0.61 (0.53, 0.68)	-0.59 (-0.71, -0.46)
Porto	-68.9 (-77.9, -59.5)	18.3 (15.2, 22.3)	-87.2 (-95.4, -79.5)	0.89 (0.7, 1.04)	-0.77 (-0.94, -0.59)
London	-70.2 (-83.9, -54.4)	15.1 (10.5, 19.8)	-85.2 (-98.5, -70.5)	1.78 (1.3, 2.19)	-1.45 (-1.78, -1.11)

Table S7: Economic impact of years of life lost associated with UHI, in 2021-EUR per urban adult inhabitant, local population age structure. Values in brackets indicate the 90% confidence interval bounds. (continued)

City	Net [EUR per year]	Heat [EUR per year]	Cold [EUR per year]	Heat extreme [EUR per day]	Cold extreme [EUR per day]
Glasgow	-84.4 (-97.8, -69.7)	5.7 (3.7, 8.5)	-90.1 (-103.4, -75.7)	0.73 (0.44, 0.98)	-1.19 (-1.5, -0.86)

Table S8: Mortality associated with UHI, per 100,000 urban adult population, age-standardised. Values in brackets indicate the 90% confidence interval bounds.

City	Net [per year]	Heat [per year]	Cold [per year]	Heat extreme [per day]	Cold extreme [per day]
Trieste	3.3 (1.7, 5.0)	9.4 (8.0, 10.6)	-6.1 (-7.2, -4.9)	0.24 (0.18, 0.29)	-0.03 (-0.05, -0.01)
Genoa	2.9 (0.7, 5.3)	10.0 (8.2, 11.3)	-7.1 (-8.5, -5.0)	0.2 (0.15, 0.25)	-0.06 (-0.07, -0.04)
Thessaloniki	2.7 (-0.4, 5.7)	13.6 (10.8, 16.1)	-11.0 (-12.9, -8.8)	0.33 (0.21, 0.43)	-0.06 (-0.08, -0.04)
Bologna	2.3 (0.8, 3.9)	10.1 (9.1, 11.0)	-7.8 (-9.1, -6.5)	0.22 (0.18, 0.26)	-0.01 (-0.05, 0.03)
Ljubljana	2.0 (0.1, 4.2)	7.6 (6.0, 9.2)	-5.6 (-7.0, -4.0)	0.2 (0.11, 0.27)	-0.01 (-0.04, 0.02)
Zagreb	1.9 (-0.9, 4.6)	10.0 (7.6, 12.1)	-8.1 (-10.2, -6.0)	0.19 (0.08, 0.29)	-0.02 (-0.05, 0.01)
Vienna	1.6 (-0.7, 3.7)	8.1 (6.4, 9.9)	-6.4 (-8.2, -5.0)	0.35 (0.26, 0.42)	-0.04 (-0.07, -0.0)
Madrid	1.5 (-0.6, 3.5)	6.3 (5.0, 7.8)	-4.8 (-6.6, -3.2)	0.26 (0.19, 0.32)	-0.08 (-0.12, -0.04)
Graz	1.5 (-0.8, 3.7)	6.4 (5.0, 8.4)	-4.9 (-7.0, -3.5)	0.3 (0.21, 0.37)	0.0 (-0.03, 0.04)
Bucharest	1.0 (-3.1, 4.7)	16.8 (13.9, 19.9)	-15.8 (-18.8, -13.3)	0.65 (0.51, 0.77)	-0.04 (-0.08, 0.01)
Geneva	0.6 (-0.4, 1.7)	4.3 (3.6, 5.0)	-3.7 (-4.5, -2.9)	0.19 (0.16, 0.22)	-0.01 (-0.03, 0.01)
Basel	0.5 (-0.8, 1.8)	6.1 (5.3, 6.9)	-5.7 (-6.7, -4.5)	0.31 (0.27, 0.34)	-0.01 (-0.04, 0.02)
Debrecen	0.4 (-2.9, 3.2)	9.1 (6.2, 11.5)	-8.8 (-10.5, -7.2)	0.24 (0.09, 0.36)	-0.02 (-0.05, 0.0)
Luxembourg	0.2 (-0.9, 1.4)	4.0 (3.3, 4.7)	-3.8 (-4.7, -2.9)	0.23 (0.2, 0.25)	0.02 (-0.02, 0.06)
Milan	0.0 (-1.7, 1.7)	10.3 (9.3, 11.4)	-10.3 (-11.7, -8.8)	0.36 (0.3, 0.42)	-0.11 (-0.15, -0.08)
Munich	0.0 (-1.4, 1.6)	4.4 (3.3, 5.4)	-4.3 (-5.5, -3.0)	0.28 (0.21, 0.33)	-0.03 (-0.05, 0.0)
Turin	-0.1 (-2.5, 2.4)	16.7 (15.0, 18.4)	-16.8 (-18.7, -14.9)	0.37 (0.29, 0.44)	-0.12 (-0.16, -0.08)
Frankfurt am Main	-0.3 (-1.2, 0.9)	4.4 (3.9, 5.0)	-4.7 (-5.6, -3.6)	0.26 (0.23, 0.28)	0.04 (0.01, 0.06)
Toulouse	-0.5 (-1.6, 0.8)	4.4 (3.7, 5.2)	-4.9 (-5.9, -3.9)	0.22 (0.18, 0.26)	-0.1 (-0.12, -0.07)
Liege	-0.7 (-2.0, 0.7)	5.2 (4.4, 6.1)	-5.8 (-7.1, -4.8)	0.32 (0.27, 0.36)	-0.03 (-0.05, -0.0)
Kosice	-0.8 (-5.0, 2.7)	11.3 (7.7, 14.2)	-12.1 (-14.3, -9.9)	0.31 (0.15, 0.45)	-0.02 (-0.05, 0.01)
Zurich	-0.8 (-2.2, 0.8)	4.7 (3.9, 5.7)	-5.5 (-6.8, -4.3)	0.23 (0.19, 0.27)	0.01 (-0.01, 0.04)

Table S8: Mortality associated with UHI, per 100,000 urban adult population, age-standardised. Values in brackets indicate the 90% confidence interval bounds. (continued)

City	Net [per year]	Heat [per year]	Cold [per year]	Heat extreme [per day]	Cold extreme [per day]
Marseille	-0.9 (-2.8, 1.1)	5.9 (4.8, 6.9)	-6.8 (-8.4, -5.1)	0.29 (0.23, 0.34)	-0.12 (-0.14, -0.09)
Palermo	-1.0 (-2.5, 0.6)	7.8 (6.6, 8.3)	-8.8 (-9.8, -7.1)	0.14 (0.1, 0.18)	-0.05 (-0.06, -0.04)
Charleroi	-1.0 (-2.4, 0.5)	5.1 (4.2, 6.2)	-6.1 (-7.3, -5.0)	0.29 (0.24, 0.33)	-0.06 (-0.09, -0.02)
Cologne	-1.2 (-2.1, -0.3)	4.7 (4.3, 5.2)	-5.9 (-6.7, -5.1)	0.29 (0.26, 0.31)	0.01 (-0.01, 0.04)
Pecs	-1.3 (-4.8, 2.2)	9.7 (6.6, 12.7)	-10.9 (-13.2, -8.6)	0.29 (0.16, 0.41)	-0.03 (-0.07, 0.02)
Bilbao	-1.3 (-3.3, 0.8)	5.1 (4.0, 6.3)	-6.4 (-8.0, -4.9)	0.22 (0.17, 0.27)	-0.06 (-0.07, -0.05)
Dusseldorf	-1.4 (-2.4, -0.3)	6.7 (6.1, 7.4)	-8.0 (-9.0, -7.2)	0.37 (0.34, 0.4)	0.03 (-0.01, 0.07)
Nice	-1.5 (-2.5, -0.5)	3.3 (2.7, 3.7)	-4.8 (-5.7, -3.8)	0.13 (0.11, 0.15)	-0.04 (-0.04, -0.03)
Split	-1.5 (-4.5, 1.1)	9.9 (7.6, 11.9)	-11.5 (-13.9, -9.1)	0.22 (0.11, 0.3)	-0.07 (-0.09, -0.05)
Warsaw	-1.7 (-3.7, 0.2)	4.7 (3.5, 6.2)	-6.4 (-8.1, -5.0)	0.31 (0.22, 0.38)	-0.02 (-0.04, 0.01)
Murcia	-1.9 (-3.5, -0.3)	5.7 (4.6, 6.8)	-7.6 (-8.8, -6.4)	0.17 (0.11, 0.21)	-0.11 (-0.13, -0.1)
Stockholm	-2.0 (-2.2, -1.6)	0.4 (0.3, 0.5)	-2.3 (-2.6, -2.0)	0.05 (0.03, 0.06)	-0.01 (-0.01, -0.0)
Berlin	-2.2 (-3.3, -1.1)	4.2 (3.8, 4.7)	-6.5 (-7.4, -5.5)	0.3 (0.27, 0.33)	-0.01 (-0.03, 0.01)
Krakow	-2.4 (-4.9, 0.1)	7.6 (5.7, 9.6)	-10.0 (-11.8, -8.4)	0.31 (0.22, 0.39)	-0.02 (-0.03, -0.0)
Varna	-2.6 (-7.5, 2.2)	13.6 (9.4, 17.6)	-16.1 (-18.8, -13.1)	0.36 (0.24, 0.48)	-0.05 (-0.07, -0.03)
Vilnius	-2.6 (-6.4, 0.8)	3.2 (-0.1, 6.0)	-5.8 (-8.3, -3.4)	0.09 (-0.19, 0.32)	-0.0 (-0.04, 0.05)
Budapest	-2.6 (-6.0, 0.4)	9.3 (6.9, 11.9)	-12.0 (-14.6, -9.7)	0.42 (0.3, 0.52)	-0.03 (-0.06, -0.0)
Antwerp	-2.7 (-4.1, -1.2)	3.8 (3.1, 4.7)	-6.5 (-7.8, -5.3)	0.28 (0.22, 0.32)	-0.07 (-0.11, -0.04)
Bratislava	-2.8 (-5.6, -0.2)	6.3 (4.4, 8.4)	-9.0 (-11.3, -7.1)	0.28 (0.17, 0.36)	-0.02 (-0.06, 0.02)
Lille	-2.8 (-4.2, -1.5)	3.7 (3.1, 4.4)	-6.5 (-7.8, -5.3)	0.21 (0.17, 0.25)	-0.1 (-0.14, -0.04)
Brussels	-2.9 (-4.2, -1.4)	4.6 (4.0, 5.5)	-7.5 (-8.7, -6.4)	0.32 (0.27, 0.36)	-0.09 (-0.13, -0.05)
Leipzig	-2.9 (-4.2, -1.6)	5.9 (5.1, 6.8)	-8.8 (-10.0, -7.7)	0.24 (0.2, 0.27)	-0.03 (-0.06, 0.0)

Table S8: Mortality associated with UHI, per 100,000 urban adult population, age-standardised. Values in brackets indicate the 90% confidence interval bounds. (continued)

City	Net [per year]	Heat [per year]	Cold [per year]	Heat extreme [per day]	Cold extreme [per day]
Ghent	-2.9 (-4.5, -1.3)	3.3 (2.6, 4.1)	-6.3 (-7.6, -4.9)	0.25 (0.2, 0.29)	-0.08 (-0.13, -0.03)
Nantes	-3.1 (-4.3, -1.8)	4.0 (3.4, 4.5)	-7.0 (-8.1, -6.0)	0.22 (0.18, 0.25)	-0.09 (-0.13, -0.05)
Miskolc	-3.2 (-8.9, 2.0)	16.4 (11.2, 21.0)	-19.5 (-23.2, -15.8)	0.34 (0.12, 0.53)	-0.04 (-0.11, 0.03)
Riga	-3.3 (-5.4, -1.2)	1.8 (0.4, 3.4)	-5.0 (-6.7, -3.4)	0.05 (-0.07, 0.16)	-0.02 (-0.03, -0.0)
Naples	-3.4 (-5.0, -1.8)	5.6 (4.7, 6.6)	-9.0 (-10.5, -7.6)	0.2 (0.14, 0.25)	-0.08 (-0.1, -0.07)
Helsinki	-3.7 (-4.3, -2.9)	1.3 (0.8, 1.7)	-4.9 (-5.6, -4.2)	0.14 (0.09, 0.18)	-0.03 (-0.04, -0.02)
Hamburg	-3.7 (-4.7, -2.6)	2.6 (2.1, 3.2)	-6.3 (-7.3, -5.3)	0.23 (0.19, 0.28)	-0.09 (-0.11, -0.07)
Cluj-Napoca	-3.7 (-8.6, 0.5)	13.9 (10.1, 17.4)	-17.7 (-20.6, -14.7)	0.27 (0.12, 0.42)	-0.05 (-0.08, -0.01)
Sofia	-3.8 (-7.8, -0.1)	9.4 (6.7, 12.4)	-13.1 (-16.1, -10.7)	0.36 (0.26, 0.47)	-0.01 (-0.02, 0.01)
Lyon	-3.8 (-5.6, -2.1)	6.0 (4.7, 7.3)	-9.8 (-11.2, -8.4)	0.28 (0.22, 0.34)	-0.18 (-0.2, -0.15)
Montpellier	-3.9 (-5.2, -2.6)	5.2 (4.5, 6.0)	-9.1 (-10.2, -8.0)	0.27 (0.23, 0.3)	-0.08 (-0.11, -0.04)
Rome	-3.9 (-5.2, -2.5)	8.4 (7.5, 9.3)	-12.3 (-13.4, -11.1)	0.23 (0.18, 0.27)	-0.08 (-0.1, -0.06)
Gyor	-4.2 (-8.4, -0.2)	10.0 (7.0, 13.5)	-14.2 (-17.2, -11.7)	0.36 (0.21, 0.5)	-0.07 (-0.12, -0.02)
Gdansk	-4.2 (-6.2, -2.1)	2.5 (1.6, 3.8)	-6.7 (-8.5, -5.1)	0.17 (0.11, 0.23)	-0.04 (-0.07, -0.01)
Tallinn	-4.3 (-5.8, -2.8)	1.9 (1.0, 2.9)	-6.2 (-7.6, -5.0)	0.17 (0.06, 0.25)	-0.02 (-0.04, -0.01)
Paris	-4.5 (-7.3, -1.4)	5.5 (4.4, 6.6)	-10.0 (-12.6, -7.1)	0.37 (0.31, 0.42)	-0.16 (-0.22, -0.09)
Bari	-4.5 (-5.6, -3.4)	2.9 (2.5, 3.4)	-7.5 (-8.5, -6.4)	0.07 (0.05, 0.09)	-0.07 (-0.08, -0.05)
Wroclaw	-4.5 (-6.6, -2.4)	4.8 (3.6, 6.3)	-9.3 (-11.0, -7.9)	0.2 (0.14, 0.26)	-0.04 (-0.07, -0.01)
Bordeaux	-4.5 (-5.7, -3.4)	4.0 (3.3, 4.7)	-8.6 (-9.5, -7.5)	0.26 (0.22, 0.3)	-0.15 (-0.17, -0.12)
Prague	-4.8 (-8.4, -1.4)	5.7 (3.9, 7.8)	-10.6 (-13.7, -7.6)	0.32 (0.21, 0.42)	-0.0 (-0.06, 0.05)
Rotterdam	-4.8 (-7.0, -2.6)	4.1 (3.2, 5.3)	-8.9 (-10.9, -7.2)	0.29 (0.22, 0.34)	-0.11 (-0.16, -0.06)
Amsterdam	-4.9 (-7.8, -1.8)	4.2 (3.1, 6.0)	-9.1 (-11.8, -6.8)	0.33 (0.24, 0.41)	-0.15 (-0.2, -0.08)

Table S8: Mortality associated with UHI, per 100,000 urban adult population, age-standardised. Values in brackets indicate the 90% confidence interval bounds. (continued)

City	Net [per year]	Heat [per year]	Cold [per year]	Heat extreme [per day]	Cold extreme [per day]
Utrecht	-5.2 (-7.5, -2.7)	4.7 (3.7, 6.0)	-9.8 (-12.0, -8.0)	0.31 (0.23, 0.37)	-0.16 (-0.21, -0.09)
Athens	-5.3 (-8.2, -2.4)	9.9 (8.0, 11.7)	-15.2 (-17.7, -12.7)	0.38 (0.32, 0.44)	-0.05 (-0.08, -0.03)
Strasbourg	-5.4 (-6.6, -4.3)	3.2 (2.4, 4.0)	-8.6 (-9.6, -7.7)	0.21 (0.16, 0.25)	-0.14 (-0.17, -0.1)
Sevilla	-6.1 (-8.9, -3.5)	9.2 (7.4, 11.1)	-15.4 (-17.6, -13.1)	0.22 (0.11, 0.31)	-0.34 (-0.39, -0.29)
Oslo	-6.2 (-7.9, -4.3)	0.6 (0.1, 1.0)	-6.7 (-8.5, -4.5)	0.08 (0.03, 0.13)	0.0 (-0.02, 0.02)
Malaga	-6.2 (-8.3, -4.1)	4.4 (3.7, 5.2)	-10.6 (-12.6, -8.7)	0.16 (0.12, 0.2)	-0.13 (-0.15, -0.11)
Szeged	-6.5 (-10.5, -2.7)	9.2 (5.9, 12.4)	-15.6 (-18.4, -12.9)	0.24 (0.12, 0.36)	-0.12 (-0.17, -0.06)
Klaipeda	-7.0 (-10.5, -3.2)	2.8 (0.7, 5.4)	-9.7 (-12.7, -7.2)	0.11 (-0.09, 0.28)	-0.05 (-0.09, -0.01)
Valencia	-7.0 (-8.9, -5.0)	5.3 (4.2, 6.4)	-12.3 (-13.9, -10.6)	0.2 (0.13, 0.26)	-0.29 (-0.32, -0.26)
Brasov	-7.3 (-12.6, -2.4)	7.2 (3.2, 11.5)	-14.6 (-17.7, -11.7)	0.24 (0.03, 0.43)	-0.05 (-0.08, -0.02)
Copenhagen	-7.6 (-8.7, -6.5)	0.7 (0.4, 1.2)	-8.3 (-9.4, -7.3)	0.09 (0.06, 0.13)	-0.13 (-0.16, -0.1)
Barcelona	-8.0 (-10.0, -5.8)	5.9 (4.9, 7.2)	-13.9 (-15.9, -11.9)	0.28 (0.22, 0.33)	-0.14 (-0.17, -0.12)
Leeds	-8.1 (-8.8, -7.3)	0.6 (0.4, 0.8)	-8.6 (-9.4, -7.9)	0.07 (0.05, 0.1)	-0.23 (-0.26, -0.2)
Edinburgh	-8.9 (-10.2, -7.5)	0.7 (0.5, 1.1)	-9.7 (-11.0, -8.4)	0.1 (0.07, 0.12)	-0.13 (-0.16, -0.11)
Lisbon	-9.0 (-10.0, -7.9)	2.0 (1.7, 2.3)	-10.9 (-12.0, -10.0)	0.1 (0.09, 0.11)	-0.11 (-0.13, -0.09)
Dublin	-10.6 (-11.4, -9.8)	0.4 (0.2, 0.8)	-11.1 (-11.9, -10.3)	0.06 (0.03, 0.1)	-0.11 (-0.11, -0.1)
Porto	-13.5 (-14.9, -11.9)	3.3 (2.8, 3.8)	-16.7 (-18.1, -15.4)	0.17 (0.14, 0.19)	-0.16 (-0.19, -0.13)
London	-17.0 (-19.7, -13.8)	3.5 (2.5, 4.5)	-20.5 (-23.2, -17.5)	0.41 (0.31, 0.49)	-0.34 (-0.41, -0.27)
Glasgow	-17.5 (-19.6, -15.1)	1.2 (0.8, 1.7)	-18.7 (-20.8, -16.4)	0.15 (0.11, 0.19)	-0.26 (-0.31, -0.21)

Table S9: Economic impact of mortality associated with UHI, in 2021-EUR per urban adult inhabitant, age-standardised. Values in brackets indicate the 90% confidence interval bounds.

City	Net [EUR per year]	Heat [EUR per year]	Cold [EUR per year]	Heat extreme [EUR per day]	Cold extreme [EUR per day]
Trieste	128.6 (64.7, 195.4)	368.0 (314.2, 413.3)	-239.4 (-281.4, -189.8)	9.33 (6.97, 11.38)	-1.22 (-1.98, -0.42)
Genoa	114.8 (27.0, 205.7)	392.4 (318.7, 442.3)	-277.6 (-330.9, -195.5)	7.94 (5.96, 9.77)	-2.22 (-2.79, -1.66)
Thessaloniki	105.0 (-14.8, 223.3)	533.4 (423.6, 631.1)	-428.3 (-503.2, -344.8)	12.75 (8.27, 16.64)	-2.37 (-2.97, -1.75)
Bologna	89.4 (31.0, 151.4)	394.5 (356.5, 428.9)	-305.1 (-354.2, -252.5)	8.59 (6.85, 10.12)	-0.5 (-1.96, 0.98)
Ljubljana	79.9 (4.8, 162.5)	297.9 (234.8, 359.5)	-217.9 (-273.4, -157.5)	7.68 (4.37, 10.36)	-0.33 (-1.45, 0.79)
Zagreb	74.1 (-37.0, 181.0)	389.8 (297.2, 474.9)	-315.7 (-399.4, -236.5)	7.56 (3.26, 11.4)	-0.92 (-2.1, 0.28)
Vienna	63.2 (-25.7, 142.8)	315.1 (250.0, 386.6)	-251.8 (-320.7, -197.3)	13.71 (10.36, 16.27)	-1.38 (-2.56, -0.03)
Madrid	58.8 (-24.3, 137.6)	247.6 (195.5, 306.1)	-188.8 (-256.6, -125.4)	10.22 (7.34, 12.54)	-3.02 (-4.53, -1.54)
Graz	58.1 (-30.6, 143.7)	249.9 (195.6, 327.3)	-191.7 (-272.9, -135.2)	11.73 (8.22, 14.28)	0.18 (-1.27, 1.74)
Bucharest	38.5 (-123.0, 184.2)	657.6 (541.9, 777.5)	-619.1 (-733.3, -518.4)	25.27 (19.83, 30.2)	-1.54 (-3.13, 0.31)
Geneva	22.9 (-15.7, 65.4)	169.2 (142.4, 195.0)	-146.3 (-176.5, -113.3)	7.62 (6.33, 8.66)	-0.36 (-0.99, 0.33)
Basel	17.6 (-30.1, 71.4)	239.0 (207.5, 269.1)	-221.3 (-261.2, -177.6)	12.07 (10.6, 13.32)	-0.56 (-1.69, 0.69)
Debrecen	14.0 (-115.2, 123.5)	356.9 (244.3, 449.0)	-343.0 (-411.1, -280.5)	9.27 (3.52, 14.03)	-0.93 (-1.82, 0.09)
Luxembourg	7.7 (-34.3, 54.0)	155.0 (130.6, 183.5)	-147.3 (-184.6, -111.9)	8.83 (7.64, 9.85)	0.66 (-0.71, 2.24)
Milan	0.5 (-65.8, 67.4)	402.2 (362.4, 445.9)	-401.7 (-456.0, -345.6)	14.05 (11.81, 16.25)	-4.48 (-5.94, -3.08)
Munich	0.3 (-55.8, 64.3)	170.4 (128.9, 210.4)	-170.1 (-215.3, -119.1)	10.81 (8.23, 12.88)	-1.04 (-2.11, 0.1)
Turin	-4.0 (-96.9, 92.2)	652.9 (587.7, 717.9)	-656.9 (-729.6, -581.5)	14.35 (11.4, 17.11)	-4.67 (-6.43, -2.98)
Frankfurt am Main	-10.2 (-47.0, 33.5)	173.4 (153.5, 195.5)	-183.6 (-218.6, -140.3)	10.05 (8.93, 10.98)	1.37 (0.5, 2.31)
Toulouse	-18.2 (-64.4, 30.1)	173.1 (142.8, 203.2)	-191.2 (-231.0, -152.8)	8.67 (7.07, 10.08)	-3.88 (-4.73, -2.84)
Liege	-25.6 (-78.3, 28.5)	201.5 (172.1, 239.4)	-227.1 (-276.7, -186.3)	12.44 (10.66, 14.0)	-1.17 (-2.07, -0.1)
Kosice	-30.2 (-195.6, 103.8)	441.0 (300.4, 555.5)	-471.3 (-560.2, -387.1)	12.23 (5.79, 17.54)	-0.91 (-1.9, 0.22)
Zurich	-31.3 (-87.3, 32.3)	185.2 (151.5, 224.8)	-216.5 (-266.0, -168.0)	9.18 (7.46, 10.74)	0.56 (-0.58, 1.72)

Table S9: Economic impact of mortality associated with UHI, in 2021-EUR per urban adult inhabitant, age-standardised. Values in brackets indicate the 90% confidence interval bounds. (continued)

City	Net [EUR per year]	Heat [EUR per year]	Cold [EUR per year]	Heat extreme [EUR per day]	Cold extreme [EUR per day]
Marseille	-35.6 (-108.9, 42.5)	230.6 (186.4, 270.0)	-266.2 (-327.6, -198.5)	11.23 (9.16, 13.12)	-4.52 (-5.37, -3.54)
Palermo	-37.1 (-97.2, 21.6)	305.2 (257.1, 326.0)	-342.3 (-381.3, -279.1)	5.66 (4.03, 7.07)	-1.96 (-2.22, -1.69)
Charleroi	-37.8 (-92.5, 21.1)	200.3 (164.7, 243.2)	-238.1 (-286.0, -194.8)	11.35 (9.24, 12.99)	-2.27 (-3.53, -0.76)
Cologne	-47.7 (-82.5, -12.1)	183.0 (167.1, 202.8)	-230.6 (-262.1, -201.2)	11.23 (10.16, 12.22)	0.57 (-0.46, 1.76)
Pecs	-49.7 (-188.8, 87.2)	377.8 (258.7, 496.7)	-427.5 (-515.0, -337.3)	11.37 (6.22, 15.88)	-1.09 (-2.57, 0.6)
Bilbao	-52.6 (-130.9, 32.5)	197.8 (156.4, 248.1)	-250.5 (-313.8, -191.7)	8.75 (6.76, 10.55)	-2.3 (-2.7, -1.89)
Dusseldorf	-54.3 (-93.8, -10.4)	260.5 (240.4, 288.4)	-314.8 (-350.4, -281.5)	14.58 (13.29, 15.77)	1.05 (-0.35, 2.67)
Nice	-59.2 (-99.1, -19.5)	128.6 (105.1, 146.4)	-187.8 (-222.4, -148.5)	5.15 (4.26, 5.93)	-1.5 (-1.73, -1.25)
Split	-60.3 (-176.2, 44.5)	388.1 (296.2, 463.6)	-448.4 (-542.2, -357.3)	8.48 (4.32, 11.85)	-2.67 (-3.42, -1.9)
Warsaw	-66.2 (-145.0, 8.6)	185.0 (136.0, 241.7)	-251.2 (-314.8, -196.6)	12.02 (8.63, 14.9)	-0.62 (-1.44, 0.33)
Murcia	-74.7 (-137.1, -12.5)	223.4 (180.5, 265.8)	-298.1 (-344.3, -249.4)	6.46 (4.18, 8.36)	-4.38 (-5.02, -3.72)
Stockholm	-76.4 (-87.9, -62.8)	14.6 (10.3, 19.2)	-91.0 (-102.3, -77.8)	1.84 (1.34, 2.34)	-0.27 (-0.35, -0.19)
Berlin	-87.5 (-128.6, -42.7)	165.0 (147.8, 185.2)	-252.5 (-291.2, -214.0)	11.89 (10.6, 12.96)	-0.31 (-1.11, 0.52)
Krakow	-93.5 (-192.7, 2.5)	297.9 (222.9, 376.5)	-391.4 (-459.8, -328.1)	12.22 (8.53, 15.43)	-0.73 (-1.36, -0.04)
Varna	-99.8 (-293.1, 85.4)	530.0 (367.3, 687.9)	-629.8 (-734.1, -511.4)	14.09 (9.19, 18.75)	-1.93 (-2.73, -0.98)
Vilnius	-102.9 (-249.5, 31.7)	124.3 (-5.7, 236.1)	-227.2 (-325.8, -133.5)	3.61 (-7.57, 12.34)	-0.09 (-1.52, 1.79)
Budapest	-103.2 (-232.8, 16.4)	365.2 (271.1, 466.7)	-468.4 (-569.5, -378.7)	16.33 (11.56, 20.42)	-1.25 (-2.34, -0.05)
Antwerp	-104.7 (-160.9, -47.3)	149.3 (122.8, 182.3)	-253.8 (-305.7, -206.8)	10.79 (8.78, 12.44)	-2.85 (-4.18, -1.4)
Bratislava	-108.0 (-220.5, -7.0)	245.3 (170.2, 328.9)	-353.3 (-441.1, -278.8)	10.75 (6.76, 14.09)	-0.77 (-2.23, 0.78)
Lille	-110.4 (-165.4, -57.0)	143.6 (121.9, 173.1)	-254.0 (-306.2, -208.6)	8.31 (6.58, 9.79)	-3.88 (-5.63, -1.73)
Brussels	-112.0 (-165.9, -55.7)	181.1 (155.3, 214.6)	-293.2 (-340.2, -249.0)	12.55 (10.71, 14.03)	-3.64 (-4.98, -2.03)
Leipzig	-112.4 (-162.3, -62.4)	231.9 (200.0, 267.8)	-344.3 (-391.7, -301.9)	9.39 (7.86, 10.75)	-1.09 (-2.21, 0.14)

Table S9: Economic impact of mortality associated with UHI, in 2021-EUR per urban adult inhabitant, age-standardised. Values in brackets indicate the 90% confidence interval bounds. (continued)

City	Net [EUR per year]	Heat [EUR per year]	Cold [EUR per year]	Heat extreme [EUR per day]	Cold extreme [EUR per day]
Ghent	-115.1 (-175.7, -52.4)	129.5 (103.3, 161.8)	-244.6 (-296.9, -191.1)	9.69 (7.75, 11.39)	-3.23 (-4.97, -1.27)
Nantes	-120.1 (-169.8, -71.0)	155.0 (133.5, 176.6)	-275.1 (-318.4, -234.6)	8.54 (7.19, 9.63)	-3.63 (-5.11, -1.86)
Miskolc	-123.7 (-349.8, 78.1)	639.8 (437.9, 821.4)	-763.5 (-906.7, -618.7)	13.28 (4.51, 20.66)	-1.51 (-4.16, 1.29)
Riga	-127.5 (-210.4, -46.8)	69.7 (15.4, 132.2)	-197.2 (-263.9, -134.2)	2.06 (-2.78, 6.27)	-0.72 (-1.28, -0.03)
Naples	-132.5 (-194.4, -69.9)	219.9 (183.5, 258.8)	-352.5 (-408.8, -297.9)	7.76 (5.64, 9.63)	-3.25 (-3.78, -2.68)
Helsinki	-143.6 (-170.0, -114.2)	49.4 (31.2, 66.8)	-193.1 (-218.4, -165.1)	5.47 (3.65, 7.13)	-1.11 (-1.39, -0.83)
Hamburg	-143.7 (-182.7, -101.0)	101.2 (81.6, 124.1)	-244.9 (-283.7, -205.8)	9.15 (7.34, 10.91)	-3.46 (-4.11, -2.7)
Cluj-Napoca	-146.3 (-334.3, 18.9)	545.2 (393.0, 678.7)	-691.5 (-805.4, -574.2)	10.49 (4.54, 16.43)	-2.0 (-3.27, -0.55)
Sofia	-146.8 (-304.6, -5.6)	366.7 (261.9, 485.0)	-513.6 (-630.1, -417.5)	14.2 (10.06, 18.29)	-0.32 (-0.92, 0.3)
Lyon	-148.0 (-219.1, -80.5)	235.5 (183.4, 286.2)	-383.5 (-436.5, -329.8)	11.12 (8.57, 13.36)	-6.85 (-7.7, -5.88)
Montpellier	-151.2 (-202.8, -100.4)	203.9 (176.8, 234.5)	-355.1 (-399.5, -313.4)	10.43 (8.81, 11.79)	-3.03 (-4.18, -1.68)
Rome	-152.4 (-204.0, -99.3)	326.9 (292.5, 362.9)	-479.4 (-524.0, -433.9)	8.87 (7.2, 10.37)	-3.21 (-3.91, -2.43)
Gyor	-162.3 (-326.5, -8.7)	392.8 (272.4, 527.1)	-555.1 (-673.8, -458.5)	13.98 (8.18, 19.47)	-2.91 (-4.72, -0.71)
Gdansk	-162.7 (-242.8, -81.8)	99.4 (63.3, 148.8)	-262.1 (-332.3, -198.9)	6.76 (4.17, 9.05)	-1.69 (-2.66, -0.55)
Tallinn	-167.9 (-226.0, -108.4)	73.8 (40.8, 113.4)	-241.7 (-297.1, -195.8)	6.46 (2.47, 9.88)	-0.96 (-1.43, -0.42)
Paris	-176.4 (-285.3, -56.3)	214.1 (171.1, 259.9)	-390.5 (-492.6, -278.6)	14.51 (12.27, 16.31)	-6.18 (-8.62, -3.44)
Bari	-176.7 (-220.9, -133.8)	115.3 (99.5, 131.1)	-292.0 (-334.1, -248.4)	2.88 (2.14, 3.51)	-2.7 (-3.28, -2.09)
Wroclaw	-176.9 (-256.9, -95.6)	188.0 (139.0, 245.3)	-365.0 (-431.7, -310.2)	7.85 (5.63, 10.07)	-1.57 (-2.55, -0.36)
Bordeaux	-177.6 (-223.3, -133.7)	156.9 (128.5, 185.3)	-334.6 (-373.3, -293.6)	10.29 (8.45, 11.82)	-5.79 (-6.71, -4.71)
Prague	-188.0 (-327.6, -53.7)	224.7 (153.2, 303.7)	-412.8 (-536.0, -298.7)	12.5 (8.36, 16.24)	-0.12 (-2.3, 2.08)
Rotterdam	-189.5 (-272.1, -103.6)	158.6 (125.8, 208.8)	-348.1 (-426.5, -282.3)	11.14 (8.57, 13.28)	-4.38 (-6.2, -2.17)
Amsterdam	-193.2 (-306.9, -69.6)	162.7 (121.1, 234.1)	-356.0 (-461.0, -265.2)	12.99 (9.19, 16.12)	-5.85 (-7.92, -3.23)

Table S9: Economic impact of mortality associated with UHI, in 2021-EUR per urban adult inhabitant, age-standardised. Values in brackets indicate the 90% confidence interval bounds. (continued)

City	Net [EUR per year]	Heat [EUR per year]	Cold [EUR per year]	Heat extreme [EUR per day]	Cold extreme [EUR per day]
Utrecht	-201.5 (-294.4, -107.0)	182.7 (144.3, 236.5)	-384.2 (-467.9, -313.4)	12.25 (9.16, 14.65)	-6.07 (-8.16, -3.49)
Athens	-206.6 (-319.8, -92.4)	386.6 (314.0, 459.2)	-593.2 (-692.7, -498.0)	14.99 (12.36, 17.4)	-2.14 (-2.98, -1.23)
Strasbourg	-212.4 (-259.1, -169.8)	124.8 (94.6, 156.3)	-337.2 (-375.3, -301.1)	8.07 (6.18, 9.73)	-5.4 (-6.53, -4.04)
Sevilla	-239.2 (-348.5, -136.9)	361.4 (288.3, 432.9)	-600.6 (-687.2, -513.7)	8.42 (4.47, 12.09)	-13.27 (-15.39, -11.19)
Oslo	-241.5 (-308.1, -167.5)	22.2 (4.4, 37.4)	-263.8 (-333.5, -176.2)	3.3 (1.18, 5.16)	0.09 (-0.67, 0.95)
Malaga	-243.0 (-323.5, -159.1)	173.0 (145.1, 204.3)	-416.0 (-490.9, -340.2)	6.19 (4.56, 7.67)	-5.27 (-6.04, -4.5)
Szeged	-252.7 (-412.3, -104.3)	358.0 (230.3, 486.3)	-610.8 (-717.9, -502.7)	9.54 (4.78, 14.05)	-4.77 (-6.62, -2.52)
Klaipeda	-271.8 (-409.6, -126.7)	107.8 (26.4, 211.7)	-379.6 (-495.9, -279.6)	4.48 (-3.45, 11.09)	-2.09 (-3.35, -0.53)
Valencia	-273.6 (-346.5, -196.6)	207.5 (163.9, 251.8)	-481.2 (-543.1, -414.5)	7.64 (5.02, 10.09)	-11.25 (-12.51, -10.0)
Brasov	-286.3 (-493.3, -93.7)	282.7 (124.6, 450.5)	-569.1 (-692.9, -458.5)	9.23 (1.1, 16.73)	-1.94 (-2.94, -0.83)
Copenhagen	-298.1 (-338.6, -253.5)	28.0 (17.3, 46.5)	-326.2 (-366.5, -287.1)	3.68 (2.23, 4.97)	-5.1 (-6.16, -3.87)
Barcelona	-311.7 (-392.8, -226.9)	232.5 (189.7, 283.1)	-544.3 (-621.6, -466.6)	10.94 (8.76, 12.75)	-5.66 (-6.66, -4.64)
Leeds	-316.2 (-345.0, -286.9)	21.9 (14.3, 29.5)	-338.1 (-367.4, -307.7)	2.86 (1.95, 3.75)	-9.09 (-10.29, -7.9)
Edinburgh	-349.3 (-399.4, -294.2)	28.7 (21.5, 42.9)	-378.0 (-429.6, -326.9)	3.85 (2.72, 4.87)	-5.19 (-6.07, -4.28)
Lisbon	-350.6 (-390.5, -308.7)	77.2 (66.9, 91.0)	-427.9 (-468.8, -389.4)	3.86 (3.38, 4.25)	-4.18 (-4.89, -3.41)
Dublin	-415.5 (-447.1, -382.4)	17.0 (9.1, 30.0)	-432.6 (-467.0, -402.9)	2.54 (1.08, 3.83)	-4.15 (-4.46, -3.87)
Porto	-526.6 (-583.0, -464.6)	127.2 (109.4, 149.4)	-653.9 (-709.3, -602.5)	6.59 (5.46, 7.6)	-6.37 (-7.44, -5.22)
London	-663.9 (-771.5, -539.9)	137.3 (99.1, 176.3)	-801.4 (-905.9, -683.0)	15.85 (12.15, 19.11)	-13.36 (-15.87, -10.72)
Glasgow	-684.5 (-765.3, -588.9)	48.0 (32.9, 66.2)	-732.7 (-814.2, -641.4)	6.02 (4.13, 7.62)	-10.24 (-12.14, -8.06)

Table S10: Years of life lost associated with UHI, per 100,000 urban adult population, age-standardised. Values in brackets indicate the 90% confidence interval bounds.

City	Net [per year]	Heat [per year]	Cold [per year]	Heat extreme [per day]	Cold extreme [per day]
Trieste	48.1 (25.1, 71.3)	120.9 (101.8, 137.5)	-72.9 (-87.7, -56.4)	2.96 (2.12, 3.72)	-0.24 (-0.52, 0.06)
Genoa	45.2 (13.5, 76.0)	127.6 (102.1, 145.5)	-82.4 (-100.9, -55.3)	2.48 (1.78, 3.12)	-0.64 (-0.84, -0.45)
Thessaloniki	39.6 (1.7, 76.5)	167.7 (134.2, 198.2)	-128.1 (-153.4, -100.6)	3.83 (2.22, 5.06)	-0.67 (-0.88, -0.47)
Bologna	37.0 (17.4, 57.5)	127.6 (115.0, 139.5)	-90.6 (-107.4, -73.5)	2.66 (2.09, 3.2)	0.15 (-0.35, 0.67)
Ljubljana	34.2 (6.3, 63.7)	100.6 (79.7, 123.6)	-66.4 (-88.0, -46.8)	2.5 (1.31, 3.5)	0.08 (-0.33, 0.52)
Madrid	28.2 (-2.8, 57.9)	86.9 (68.0, 109.4)	-58.7 (-85.6, -36.6)	3.55 (2.43, 4.45)	-0.84 (-1.42, -0.27)
Vienna	27.8 (-2.3, 53.9)	100.9 (78.8, 125.1)	-73.0 (-96.3, -53.9)	4.38 (3.15, 5.31)	-0.22 (-0.67, 0.26)
Graz	27.3 (-2.2, 53.4)	70.6 (55.2, 98.2)	-43.3 (-74.6, -26.6)	3.58 (2.34, 4.46)	0.38 (-0.13, 0.93)
Zagreb	26.0 (-5.0, 56.3)	104.8 (79.4, 126.6)	-78.7 (-102.1, -54.6)	1.88 (0.63, 2.94)	-0.12 (-0.45, 0.24)
Bucharest	21.1 (-31.6, 65.8)	196.7 (157.4, 234.0)	-175.6 (-211.2, -141.4)	7.48 (5.77, 9.09)	-0.23 (-0.75, 0.39)
Basel	16.1 (-0.5, 34.6)	79.1 (67.5, 89.5)	-63.0 (-75.9, -48.0)	3.91 (3.34, 4.36)	0.18 (-0.25, 0.67)
Geneva	13.8 (-0.5, 29.0)	58.9 (48.3, 68.6)	-45.2 (-55.3, -33.3)	2.55 (2.07, 2.94)	0.05 (-0.19, 0.33)
Luxembourg	10.8 (-4.4, 26.4)	51.1 (42.8, 62.4)	-40.3 (-53.1, -28.4)	2.9 (2.44, 3.28)	0.59 (0.08, 1.17)
Milan	8.2 (-14.8, 31.4)	131.1 (117.5, 146.1)	-123.0 (-141.9, -103.6)	4.5 (3.71, 5.23)	-1.22 (-1.74, -0.71)
Turin	7.9 (-23.9, 40.0)	208.2 (185.7, 230.1)	-200.3 (-224.9, -174.7)	4.42 (3.39, 5.35)	-1.19 (-1.78, -0.59)
Debrecen	7.1 (-33.5, 40.7)	102.7 (66.1, 132.7)	-95.5 (-116.7, -75.5)	2.43 (0.53, 3.96)	-0.14 (-0.44, 0.19)
Munich	7.1 (-11.9, 29.0)	52.5 (37.7, 66.7)	-45.4 (-60.7, -28.4)	3.38 (2.47, 4.13)	-0.1 (-0.48, 0.31)
Frankfurt am Main	3.9 (-9.2, 18.8)	54.8 (47.4, 62.7)	-50.8 (-62.9, -34.6)	3.23 (2.81, 3.56)	0.69 (0.38, 1.02)
Toulouse	3.4 (-16.0, 22.9)	63.9 (52.4, 76.5)	-60.5 (-76.4, -46.7)	3.18 (2.49, 3.77)	-1.24 (-1.63, -0.78)
Liege	2.0 (-16.1, 21.6)	66.8 (56.1, 81.6)	-64.8 (-80.9, -51.9)	3.95 (3.27, 4.5)	-0.09 (-0.43, 0.3)
Marseille	0.7 (-29.8, 32.4)	84.2 (67.4, 101.2)	-83.5 (-109.0, -58.4)	4.07 (3.19, 4.86)	-1.44 (-1.82, -1.02)
Zurich	-1.5 (-20.7, 19.7)	60.5 (47.4, 74.3)	-61.9 (-78.1, -45.3)	2.84 (2.24, 3.37)	0.46 (0.06, 0.87)

Table S10: Years of life lost associated with UHI, per 100,000 urban adult population, age-standardised. Values in brackets indicate the 90% confidence interval bounds. (continued)

City	Net [per year]	Heat [per year]	Cold [per year]	Heat extreme [per day]	Cold extreme [per day]
Charleroi	-1.9 (-22.1, 18.4)	65.3 (51.9, 80.5)	-67.2 (-83.9, -52.5)	3.6 (2.8, 4.19)	-0.37 (-0.87, 0.22)
Cologne	-4.4 (-16.7, 7.9)	60.9 (54.0, 68.8)	-65.4 (-75.3, -55.6)	3.54 (3.13, 3.89)	0.59 (0.18, 1.03)
Kosice	-5.7 (-60.7, 38.4)	132.7 (84.3, 170.0)	-138.4 (-166.3, -110.2)	3.45 (1.21, 5.28)	-0.11 (-0.46, 0.26)
Dusseldorf	-5.8 (-20.4, 9.2)	87.2 (78.8, 97.0)	-93.0 (-105.0, -82.2)	4.64 (4.16, 5.05)	0.84 (0.31, 1.44)
Palermo	-6.7 (-25.9, 12.0)	95.1 (79.4, 102.2)	-101.8 (-114.8, -81.4)	1.66 (1.1, 2.13)	-0.58 (-0.66, -0.49)
Bilbao	-6.8 (-35.4, 23.0)	74.9 (58.5, 94.2)	-81.7 (-103.0, -61.5)	2.96 (2.21, 3.62)	-0.74 (-0.88, -0.58)
Pecs	-10.2 (-56.5, 32.5)	111.0 (72.3, 149.4)	-121.2 (-149.0, -90.7)	3.18 (1.52, 4.63)	-0.09 (-0.58, 0.49)
Split	-10.8 (-44.6, 20.8)	110.4 (84.8, 132.8)	-121.2 (-148.3, -93.9)	2.23 (1.0, 3.25)	-0.67 (-0.9, -0.42)
Nice	-16.3 (-33.0, -0.2)	47.9 (39.0, 56.3)	-64.2 (-78.5, -50.0)	1.88 (1.5, 2.21)	-0.52 (-0.61, -0.42)
Warsaw	-18.9 (-47.7, 10.4)	63.4 (46.7, 85.6)	-82.4 (-106.1, -61.9)	4.15 (2.84, 5.23)	-0.08 (-0.42, 0.3)
Varna	-19.2 (-74.5, 32.0)	142.7 (93.9, 186.9)	-161.9 (-193.1, -129.3)	3.61 (2.23, 4.96)	-0.42 (-0.66, -0.14)
Murcia	-19.6 (-40.7, 0.8)	69.4 (54.5, 84.4)	-89.0 (-104.4, -72.9)	1.89 (1.12, 2.55)	-1.29 (-1.51, -1.06)
Berlin	-20.0 (-35.2, -4.5)	55.7 (48.9, 64.1)	-75.7 (-90.4, -62.4)	3.98 (3.48, 4.37)	0.14 (-0.19, 0.45)
Stockholm	-22.5 (-26.4, -18.2)	4.0 (2.6, 5.5)	-26.6 (-30.3, -22.1)	0.51 (0.34, 0.68)	-0.06 (-0.09, -0.03)
Lille	-24.9 (-45.3, -5.0)	48.1 (40.4, 60.0)	-73.0 (-93.3, -57.6)	2.78 (2.12, 3.36)	-0.85 (-1.58, 0.02)
Antwerp	-25.4 (-45.6, -6.4)	48.9 (39.8, 60.5)	-74.2 (-91.1, -59.6)	3.47 (2.75, 4.06)	-0.63 (-1.14, -0.1)
Krakow	-26.6 (-62.0, 6.1)	95.8 (68.9, 122.0)	-122.4 (-146.4, -100.6)	3.85 (2.55, 5.0)	-0.12 (-0.34, 0.13)
Budapest	-27.4 (-69.0, 12.7)	112.4 (81.0, 146.3)	-139.9 (-172.4, -110.3)	5.0 (3.42, 6.43)	-0.22 (-0.61, 0.21)
Brussels	-27.5 (-46.5, -7.9)	59.4 (50.5, 71.7)	-86.8 (-103.5, -73.0)	4.09 (3.41, 4.64)	-0.89 (-1.39, -0.28)
Leipzig	-27.9 (-46.5, -9.4)	79.7 (67.9, 92.6)	-107.7 (-124.0, -92.3)	3.07 (2.48, 3.59)	-0.02 (-0.47, 0.49)
Bratislava	-27.9 (-65.4, 4.1)	71.1 (45.8, 97.8)	-99.0 (-127.7, -73.8)	3.12 (1.81, 4.22)	-0.0 (-0.49, 0.53)
Ghent	-28.3 (-48.8, -6.6)	42.5 (33.8, 54.8)	-70.8 (-88.4, -54.3)	3.13 (2.41, 3.73)	-0.66 (-1.34, 0.1)

Table S10: Years of life lost associated with UHI, per 100,000 urban adult population, age-standardised. Values in brackets indicate the 90% confidence interval bounds. (continued)

City	Net [per year]	Heat [per year]	Cold [per year]	Heat extreme [per day]	Cold extreme [per day]
Miskolc	-28.4 (-105.0, 35.8)	185.4 (118.3, 242.5)	-213.8 (-259.3, -166.8)	3.49 (0.66, 5.83)	-0.06 (-0.92, 0.87)
Vilnius	-30.1 (-79.8, 18.0)	33.9 (-8.5, 77.0)	-64.0 (-102.9, -31.0)	0.73 (-3.23, 3.92)	0.16 (-0.39, 0.86)
Nantes	-31.4 (-51.8, -11.4)	59.9 (49.9, 69.3)	-91.3 (-108.1, -74.6)	3.17 (2.57, 3.64)	-0.91 (-1.59, -0.14)
Sofia	-32.6 (-78.5, 11.2)	104.2 (70.3, 140.3)	-136.8 (-172.8, -105.7)	3.88 (2.49, 5.12)	0.01 (-0.18, 0.21)
Cluj-Napoca	-34.2 (-90.5, 15.3)	149.8 (104.7, 189.4)	-184.0 (-217.7, -148.9)	2.53 (0.8, 4.36)	-0.35 (-0.75, 0.1)
Naples	-36.1 (-56.1, -16.2)	67.9 (55.9, 80.4)	-104.0 (-121.9, -86.4)	2.29 (1.59, 2.91)	-0.94 (-1.11, -0.75)
Riga	-38.5 (-67.2, -9.5)	22.0 (2.6, 43.7)	-60.4 (-83.2, -37.1)	0.43 (-1.31, 1.93)	-0.17 (-0.38, 0.08)
Hamburg	-39.6 (-54.3, -24.0)	32.2 (25.4, 40.6)	-71.8 (-85.8, -59.2)	2.89 (2.21, 3.53)	-0.96 (-1.24, -0.66)
Gyor	-42.0 (-94.5, 5.8)	109.2 (70.5, 152.1)	-151.2 (-186.9, -121.2)	3.79 (1.94, 5.48)	-0.57 (-1.15, 0.13)
Rome	-43.7 (-61.9, -25.1)	108.7 (96.9, 121.0)	-152.4 (-167.9, -136.7)	2.79 (2.23, 3.3)	-0.89 (-1.14, -0.62)
Montpellier	-44.3 (-65.2, -24.2)	74.8 (64.3, 87.3)	-119.2 (-137.2, -102.9)	3.76 (3.1, 4.33)	-0.68 (-1.18, -0.1)
Helsinki	-45.7 (-55.4, -35.4)	14.1 (8.2, 20.0)	-59.8 (-69.2, -50.0)	1.6 (0.96, 2.21)	-0.3 (-0.41, -0.19)
Lyon	-46.7 (-76.3, -19.6)	83.9 (64.4, 105.6)	-130.6 (-153.8, -110.5)	4.03 (2.95, 4.98)	-2.41 (-2.79, -1.97)
Rotterdam	-47.1 (-73.8, -20.6)	50.3 (38.6, 68.3)	-97.4 (-121.2, -78.2)	3.32 (2.49, 4.02)	-0.98 (-1.61, -0.23)
Prague	-49.0 (-93.3, -5.5)	63.2 (41.5, 90.2)	-112.2 (-155.2, -76.1)	3.69 (2.29, 4.9)	0.28 (-0.45, 1.01)
Amsterdam	-49.2 (-84.8, -11.4)	50.2 (36.8, 73.1)	-99.5 (-130.8, -72.8)	3.83 (2.61, 4.84)	-1.53 (-2.2, -0.69)
Utrecht	-50.8 (-79.1, -20.0)	56.5 (44.0, 75.2)	-107.3 (-133.0, -86.0)	3.63 (2.63, 4.41)	-1.51 (-2.22, -0.71)
Gdansk	-50.8 (-78.7, -21.1)	32.2 (19.8, 50.7)	-83.0 (-108.1, -61.3)	2.2 (1.21, 3.02)	-0.45 (-0.83, -0.02)
Paris	-52.3 (-97.2, -2.5)	83.2 (66.6, 103.7)	-135.4 (-178.8, -92.0)	5.72 (4.78, 6.49)	-2.12 (-3.21, -0.97)
Bari	-52.4 (-66.7, -38.0)	36.8 (31.4, 42.3)	-89.1 (-103.5, -75.0)	0.87 (0.62, 1.09)	-0.76 (-0.96, -0.55)
Tallinn	-53.4 (-73.7, -31.8)	21.5 (10.2, 36.3)	-75.0 (-95.5, -58.7)	1.88 (0.45, 3.15)	-0.22 (-0.41, -0.01)
Athens	-53.9 (-92.9, -14.2)	129.7 (105.9, 155.2)	-183.7 (-218.0, -152.3)	4.78 (3.88, 5.62)	-0.53 (-0.83, -0.22)

Table S10: Years of life lost associated with UHI, per 100,000 urban adult population, age-standardised. Values in brackets indicate the 90% confidence interval bounds. (continued)

City	Net [per year]	Heat [per year]	Cold [per year]	Heat extreme [per day]	Cold extreme [per day]
Wroclaw	-54.6 (-82.7, -25.4)	62.2 (44.4, 83.5)	-116.8 (-140.7, -97.1)	2.54 (1.71, 3.32)	-0.34 (-0.73, 0.12)
Bordeaux	-56.5 (-75.1, -38.3)	56.5 (44.6, 68.0)	-113.0 (-129.2, -97.2)	3.73 (2.94, 4.39)	-1.9 (-2.33, -1.43)
Strasbourg	-67.1 (-85.9, -49.8)	42.0 (30.6, 54.9)	-109.1 (-124.7, -95.4)	2.72 (1.92, 3.4)	-1.55 (-2.08, -0.94)
Szeged	-67.8 (-117.3, -21.1)	103.6 (63.7, 142.1)	-171.4 (-205.0, -137.1)	2.57 (1.04, 4.0)	-1.18 (-1.8, -0.43)
Sevilla	-69.0 (-106.6, -35.3)	111.8 (86.7, 135.4)	-180.7 (-208.9, -152.6)	2.4 (1.06, 3.63)	-3.97 (-4.68, -3.25)
Malaga	-69.5 (-96.6, -42.0)	55.1 (45.7, 66.1)	-124.7 (-150.6, -98.5)	1.87 (1.33, 2.37)	-1.56 (-1.82, -1.3)
Oslo	-70.2 (-91.8, -45.8)	3.9 (-7.8, 9.4)	-74.2 (-97.4, -43.6)	0.91 (0.24, 1.52)	0.15 (-0.11, 0.44)
Klaipeda	-80.6 (-129.1, -30.3)	31.8 (3.8, 67.5)	-112.4 (-153.9, -77.5)	0.99 (-1.58, 3.26)	-0.49 (-0.97, 0.09)
Valencia	-83.1 (-108.7, -56.5)	68.2 (53.2, 83.8)	-151.3 (-173.9, -129.0)	2.34 (1.4, 3.21)	-3.58 (-4.03, -3.12)
Brasov	-84.8 (-151.3, -21.2)	73.8 (23.3, 128.3)	-158.7 (-200.0, -123.2)	2.17 (-0.45, 4.67)	-0.43 (-0.76, -0.06)
Copenhagen	-85.2 (-98.2, -71.4)	7.4 (4.5, 12.8)	-92.7 (-105.5, -81.1)	0.99 (0.55, 1.38)	-1.37 (-1.73, -0.96)
Leeds	-89.5 (-99.8, -79.1)	5.6 (3.2, 8.0)	-95.1 (-105.3, -84.6)	0.74 (0.39, 1.05)	-2.58 (-3.0, -2.16)
Barcelona	-98.6 (-127.1, -67.4)	80.0 (64.2, 99.2)	-178.6 (-205.7, -151.7)	3.61 (2.83, 4.28)	-1.75 (-2.11, -1.37)
Edinburgh	-99.9 (-117.2, -81.9)	8.0 (5.6, 12.6)	-107.9 (-125.7, -91.4)	1.09 (0.7, 1.45)	-1.42 (-1.73, -1.1)
Lisbon	-112.3 (-127.0, -96.6)	28.4 (24.0, 33.8)	-140.8 (-155.3, -127.1)	1.3 (1.12, 1.44)	-1.25 (-1.52, -0.98)
Dublin	-125.8 (-136.4, -114.8)	4.0 (1.8, 7.9)	-129.8 (-141.0, -119.7)	0.61 (0.12, 1.03)	-1.25 (-1.35, -1.16)
Porto	-157.3 (-177.7, -136.1)	41.3 (34.6, 50.1)	-198.6 (-217.1, -181.2)	2.02 (1.61, 2.36)	-1.78 (-2.16, -1.37)
Glasgow	-194.4 (-223.7, -162.0)	13.3 (8.7, 19.5)	-207.7 (-237.4, -175.6)	1.69 (1.05, 2.25)	-2.76 (-3.46, -2.03)
London	-212.5 (-251.8, -167.4)	45.0 (31.5, 58.7)	-257.6 (-295.6, -214.6)	5.28 (3.95, 6.46)	-4.36 (-5.3, -3.41)

Table S11: Economic impact of years of life lost associated with UHI, in 2021-EUR per urban adult inhabitant, age-standardised. Values in brackets indicate the 90% confidence interval bounds.

City	Net [EUR per year]	Heat [EUR per year]	Cold [EUR per year]	Heat extreme [EUR per day]	Cold extreme [EUR per day]
Trieste	22.1 (11.6, 32.8)	55.6 (46.8, 63.3)	-33.5 (-40.4, -25.9)	1.36 (0.97, 1.71)	-0.11 (-0.24, 0.03)
Genoa	20.8 (6.2, 34.9)	58.7 (47.0, 66.9)	-37.9 (-46.4, -25.4)	1.14 (0.82, 1.43)	-0.29 (-0.38, -0.21)
Thessaloniki	18.2 (0.8, 35.2)	77.1 (61.7, 91.2)	-58.9 (-70.6, -46.3)	1.76 (1.02, 2.33)	-0.31 (-0.41, -0.22)
Bologna	17.0 (8.0, 26.4)	58.7 (52.9, 64.2)	-41.7 (-49.4, -33.8)	1.23 (0.96, 1.47)	0.07 (-0.16, 0.31)
Ljubljana	15.7 (2.9, 29.3)	46.3 (36.7, 56.9)	-30.5 (-40.5, -21.5)	1.15 (0.6, 1.61)	0.04 (-0.15, 0.24)
Madrid	13.0 (-1.3, 26.6)	40.0 (31.3, 50.3)	-27.0 (-39.4, -16.9)	1.63 (1.12, 2.05)	-0.39 (-0.66, -0.12)
Vienna	12.8 (-1.1, 24.8)	46.4 (36.3, 57.5)	-33.6 (-44.3, -24.8)	2.02 (1.45, 2.44)	-0.1 (-0.31, 0.12)
Graz	12.6 (-1.0, 24.6)	32.5 (25.4, 45.2)	-19.9 (-34.3, -12.2)	1.65 (1.08, 2.05)	0.17 (-0.06, 0.43)
Zagreb	12.0 (-2.3, 25.9)	48.2 (36.5, 58.2)	-36.2 (-46.9, -25.1)	0.86 (0.29, 1.35)	-0.05 (-0.21, 0.11)
Bucharest	9.7 (-14.5, 30.3)	90.5 (72.4, 107.6)	-80.8 (-97.2, -65.1)	3.44 (2.65, 4.18)	-0.11 (-0.35, 0.18)
Basel	7.4 (-0.2, 15.9)	36.4 (31.0, 41.2)	-29.0 (-34.9, -22.1)	1.8 (1.54, 2.01)	0.08 (-0.11, 0.31)
Geneva	6.3 (-0.2, 13.3)	27.1 (22.2, 31.6)	-20.8 (-25.4, -15.3)	1.17 (0.95, 1.35)	0.02 (-0.09, 0.15)
Luxembourg	5.0 (-2.0, 12.1)	23.5 (19.7, 28.7)	-18.5 (-24.4, -13.1)	1.33 (1.12, 1.51)	0.27 (0.04, 0.54)
Milan	3.8 (-6.8, 14.5)	60.3 (54.1, 67.2)	-56.6 (-65.3, -47.7)	2.07 (1.71, 2.41)	-0.56 (-0.8, -0.33)
Turin	3.6 (-11.0, 18.4)	95.8 (85.4, 105.8)	-92.1 (-103.4, -80.4)	2.03 (1.56, 2.46)	-0.55 (-0.82, -0.27)
Debrecen	3.3 (-15.4, 18.7)	47.2 (30.4, 61.0)	-44.0 (-53.7, -34.8)	1.12 (0.25, 1.82)	-0.07 (-0.2, 0.09)
Munich	3.3 (-5.5, 13.3)	24.1 (17.3, 30.7)	-20.9 (-27.9, -13.1)	1.56 (1.14, 1.9)	-0.04 (-0.22, 0.14)
Frankfurt am Main	1.8 (-4.2, 8.7)	25.2 (21.8, 28.8)	-23.4 (-28.9, -15.9)	1.49 (1.29, 1.64)	0.32 (0.17, 0.47)
Toulouse	1.5 (-7.4, 10.5)	29.4 (24.1, 35.2)	-27.8 (-35.1, -21.5)	1.46 (1.14, 1.73)	-0.57 (-0.75, -0.36)
Liege	0.9 (-7.4, 9.9)	30.7 (25.8, 37.5)	-29.8 (-37.2, -23.9)	1.82 (1.5, 2.07)	-0.04 (-0.2, 0.14)
Marseille	0.3 (-13.7, 14.9)	38.7 (31.0, 46.6)	-38.4 (-50.1, -26.9)	1.87 (1.47, 2.23)	-0.66 (-0.84, -0.47)
Zurich	-0.7 (-9.5, 9.1)	27.8 (21.8, 34.2)	-28.5 (-35.9, -20.8)	1.31 (1.03, 1.55)	0.21 (0.03, 0.4)

Table S11: Economic impact of years of life lost associated with UHI, in 2021-EUR per urban adult inhabitant, age-standardised. Values in brackets indicate the 90% confidence interval bounds. (continued)

City	Net [EUR per year]	Heat [EUR per year]	Cold [EUR per year]	Heat extreme [EUR per day]	Cold extreme [EUR per day]
Charleroi	-0.9 (-10.2, 8.5)	30.0 (23.9, 37.0)	-30.9 (-38.6, -24.1)	1.66 (1.29, 1.93)	-0.17 (-0.4, 0.1)
Cologne	-2.0 (-7.7, 3.6)	28.0 (24.9, 31.7)	-30.1 (-34.6, -25.6)	1.63 (1.44, 1.79)	0.27 (0.08, 0.47)
Kosice	-2.6 (-27.9, 17.7)	61.0 (38.8, 78.2)	-63.7 (-76.5, -50.7)	1.59 (0.56, 2.43)	-0.05 (-0.21, 0.12)
Dusseldorf	-2.7 (-9.4, 4.2)	40.1 (36.2, 44.6)	-42.8 (-48.3, -37.8)	2.13 (1.92, 2.32)	0.39 (0.14, 0.66)
Palermo	-3.1 (-11.9, 5.5)	43.7 (36.5, 47.0)	-46.8 (-52.8, -37.4)	0.77 (0.51, 0.98)	-0.27 (-0.31, -0.23)
Bilbao	-3.1 (-16.3, 10.6)	34.4 (26.9, 43.3)	-37.6 (-47.4, -28.3)	1.36 (1.02, 1.66)	-0.34 (-0.41, -0.27)
Pecs	-4.7 (-26.0, 14.9)	51.1 (33.3, 68.7)	-55.8 (-68.5, -41.7)	1.46 (0.7, 2.13)	-0.04 (-0.27, 0.23)
Split	-5.0 (-20.5, 9.6)	50.8 (39.0, 61.1)	-55.7 (-68.2, -43.2)	1.03 (0.46, 1.5)	-0.31 (-0.42, -0.19)
Nice	-7.5 (-15.2, -0.1)	22.0 (17.9, 25.9)	-29.5 (-36.1, -23.0)	0.87 (0.69, 1.02)	-0.24 (-0.28, -0.19)
Warsaw	-8.7 (-22.0, 4.8)	29.2 (21.5, 39.4)	-37.9 (-48.8, -28.5)	1.91 (1.31, 2.41)	-0.03 (-0.19, 0.14)
Varna	-8.8 (-34.3, 14.7)	65.6 (43.2, 86.0)	-74.5 (-88.8, -59.5)	1.66 (1.03, 2.28)	-0.19 (-0.3, -0.06)
Murcia	-9.0 (-18.7, 0.4)	31.9 (25.1, 38.8)	-40.9 (-48.0, -33.5)	0.87 (0.51, 1.18)	-0.59 (-0.69, -0.49)
Berlin	-9.2 (-16.2, -2.1)	25.6 (22.5, 29.5)	-34.8 (-41.6, -28.7)	1.83 (1.6, 2.01)	0.06 (-0.09, 0.21)
Stockholm	-10.4 (-12.1, -8.4)	1.8 (1.2, 2.5)	-12.2 (-13.9, -10.2)	0.24 (0.16, 0.31)	-0.03 (-0.04, -0.01)
Lille	-11.4 (-20.8, -2.3)	22.1 (18.6, 27.6)	-33.6 (-42.9, -26.5)	1.28 (0.97, 1.54)	-0.39 (-0.73, 0.01)
Antwerp	-11.7 (-21.0, -2.9)	22.5 (18.3, 27.8)	-34.1 (-41.9, -27.4)	1.59 (1.26, 1.87)	-0.29 (-0.52, -0.05)
Krakow	-12.3 (-28.5, 2.8)	44.1 (31.7, 56.1)	-56.3 (-67.3, -46.3)	1.77 (1.17, 2.3)	-0.06 (-0.16, 0.06)
Budapest	-12.6 (-31.7, 5.8)	51.7 (37.2, 67.3)	-64.3 (-79.3, -50.7)	2.3 (1.57, 2.96)	-0.1 (-0.28, 0.1)
Brussels	-12.6 (-21.4, -3.6)	27.3 (23.2, 33.0)	-39.9 (-47.6, -33.6)	1.88 (1.57, 2.13)	-0.41 (-0.64, -0.13)
Leipzig	-12.9 (-21.4, -4.3)	36.7 (31.2, 42.6)	-49.5 (-57.0, -42.5)	1.41 (1.14, 1.65)	-0.01 (-0.21, 0.22)
Bratislava	-12.9 (-30.1, 1.9)	32.7 (21.1, 45.0)	-45.5 (-58.8, -33.9)	1.44 (0.83, 1.94)	-0.0 (-0.23, 0.24)

Table S11: Economic impact of years of life lost associated with UHI, in 2021-EUR per urban adult inhabitant, age-standardised. Values in brackets indicate the 90% confidence interval bounds. (continued)

City	Net [EUR per year]	Heat [EUR per year]	Cold [EUR per year]	Heat extreme [EUR per day]	Cold extreme [EUR per day]
Ghent	-13.0 (-22.4, -3.0)	19.6 (15.5, 25.2)	-32.6 (-40.7, -25.0)	1.44 (1.11, 1.72)	-0.3 (-0.62, 0.05)
Miskolc	-13.1 (-48.3, 16.5)	85.3 (54.4, 111.6)	-98.3 (-119.3, -76.7)	1.61 (0.3, 2.68)	-0.03 (-0.42, 0.4)
Vilnius	-13.9 (-36.7, 8.3)	15.6 (-3.9, 35.4)	-29.4 (-47.3, -14.3)	0.34 (-1.48, 1.8)	0.07 (-0.18, 0.4)
Nantes	-14.4 (-23.8, -5.2)	27.5 (22.9, 31.9)	-42.0 (-49.7, -34.3)	1.46 (1.18, 1.67)	-0.42 (-0.73, -0.07)
Sofia	-15.0 (-36.1, 5.2)	47.9 (32.4, 64.5)	-62.9 (-79.5, -48.6)	1.79 (1.15, 2.35)	0.01 (-0.08, 0.1)
Cluj-Napoca	-15.7 (-41.6, 7.0)	68.9 (48.2, 87.1)	-84.6 (-100.2, -68.5)	1.16 (0.37, 2.01)	-0.16 (-0.34, 0.05)
Naples	-16.6 (-25.8, -7.5)	31.2 (25.7, 37.0)	-47.9 (-56.1, -39.8)	1.05 (0.73, 1.34)	-0.43 (-0.51, -0.34)
Riga	-17.7 (-30.9, -4.4)	10.1 (1.2, 20.1)	-27.8 (-38.3, -17.1)	0.2 (-0.6, 0.89)	-0.08 (-0.17, 0.04)
Hamburg	-18.2 (-25.0, -11.0)	14.8 (11.7, 18.7)	-33.0 (-39.5, -27.2)	1.33 (1.02, 1.63)	-0.44 (-0.57, -0.3)
Gyor	-19.3 (-43.5, 2.7)	50.2 (32.4, 70.0)	-69.6 (-86.0, -55.8)	1.74 (0.89, 2.52)	-0.26 (-0.53, 0.06)
Rome	-20.1 (-28.5, -11.5)	50.0 (44.6, 55.7)	-70.1 (-77.2, -62.9)	1.28 (1.02, 1.52)	-0.41 (-0.52, -0.28)
Montpellier	-20.4 (-30.0, -11.1)	34.4 (29.6, 40.2)	-54.8 (-63.1, -47.3)	1.73 (1.43, 1.99)	-0.31 (-0.54, -0.04)
Helsinki	-21.0 (-25.5, -16.3)	6.5 (3.8, 9.2)	-27.5 (-31.8, -23.0)	0.74 (0.44, 1.01)	-0.14 (-0.19, -0.09)
Lyon	-21.5 (-35.1, -9.0)	38.6 (29.6, 48.6)	-60.1 (-70.7, -50.8)	1.86 (1.36, 2.29)	-1.11 (-1.28, -0.91)
Rotterdam	-21.7 (-34.0, -9.5)	23.1 (17.8, 31.4)	-44.8 (-55.8, -36.0)	1.53 (1.15, 1.85)	-0.45 (-0.74, -0.11)
Prague	-22.5 (-42.9, -2.5)	29.1 (19.1, 41.5)	-51.6 (-71.4, -35.0)	1.7 (1.05, 2.26)	0.13 (-0.21, 0.46)
Amsterdam	-22.6 (-39.0, -5.2)	23.1 (16.9, 33.6)	-45.8 (-60.2, -33.5)	1.76 (1.2, 2.22)	-0.7 (-1.01, -0.32)
Utrecht	-23.4 (-36.4, -9.2)	26.0 (20.2, 34.6)	-49.4 (-61.2, -39.6)	1.67 (1.21, 2.03)	-0.7 (-1.02, -0.33)
Gdansk	-23.4 (-36.2, -9.7)	14.8 (9.1, 23.3)	-38.2 (-49.7, -28.2)	1.01 (0.56, 1.39)	-0.21 (-0.38, -0.01)
Paris	-24.0 (-44.7, -1.1)	38.2 (30.6, 47.7)	-62.3 (-82.3, -42.3)	2.63 (2.2, 2.98)	-0.98 (-1.48, -0.44)
Bari	-24.1 (-30.7, -17.5)	16.9 (14.4, 19.4)	-41.0 (-47.6, -34.5)	0.4 (0.28, 0.5)	-0.35 (-0.44, -0.25)

Table S11: Economic impact of years of life lost associated with UHI, in 2021-EUR per urban adult inhabitant, age-standardised. Values in brackets indicate the 90% confidence interval bounds. (continued)

City	Net [EUR per year]	Heat [EUR per year]	Cold [EUR per year]	Heat extreme [EUR per day]	Cold extreme [EUR per day]
Tallinn	-24.6 (-33.9, -14.6)	9.9 (4.7, 16.7)	-34.5 (-43.9, -27.0)	0.87 (0.21, 1.45)	-0.1 (-0.19, -0.01)
Athens	-24.8 (-42.7, -6.5)	59.7 (48.7, 71.4)	-84.5 (-100.3, -70.0)	2.2 (1.79, 2.59)	-0.25 (-0.38, -0.1)
Wroclaw	-25.1 (-38.0, -11.7)	28.6 (20.4, 38.4)	-53.7 (-64.7, -44.7)	1.17 (0.78, 1.53)	-0.16 (-0.34, 0.06)
Bordeaux	-26.0 (-34.5, -17.6)	26.0 (20.5, 31.3)	-52.0 (-59.4, -44.7)	1.72 (1.35, 2.02)	-0.88 (-1.07, -0.66)
Strasbourg	-30.9 (-39.5, -22.9)	19.3 (14.1, 25.2)	-50.2 (-57.3, -43.9)	1.25 (0.88, 1.57)	-0.71 (-0.96, -0.43)
Szeged	-31.2 (-54.0, -9.7)	47.7 (29.3, 65.4)	-78.9 (-94.3, -63.1)	1.18 (0.48, 1.84)	-0.54 (-0.83, -0.2)
Sevilla	-31.7 (-49.0, -16.3)	51.4 (39.9, 62.3)	-83.1 (-96.1, -70.2)	1.11 (0.49, 1.67)	-1.83 (-2.15, -1.5)
Malaga	-32.0 (-44.4, -19.3)	25.4 (21.0, 30.4)	-57.3 (-69.3, -45.3)	0.86 (0.61, 1.09)	-0.72 (-0.84, -0.6)
Oslo	-32.3 (-42.2, -21.0)	1.8 (-3.6, 4.3)	-34.1 (-44.8, -20.1)	0.42 (0.11, 0.7)	0.07 (-0.05, 0.2)
Klaipeda	-37.1 (-59.4, -13.9)	14.6 (1.8, 31.1)	-51.7 (-70.8, -35.7)	0.46 (-0.73, 1.5)	-0.23 (-0.45, 0.04)
Valencia	-38.2 (-50.0, -26.0)	31.4 (24.5, 38.6)	-69.6 (-80.0, -59.3)	1.08 (0.65, 1.48)	-1.65 (-1.85, -1.43)
Brasov	-39.0 (-69.6, -9.7)	34.0 (10.7, 59.0)	-73.0 (-92.0, -56.7)	1.0 (-0.2, 2.15)	-0.2 (-0.35, -0.03)
Copenhagen	-39.2 (-45.2, -32.9)	3.4 (2.1, 5.9)	-42.6 (-48.5, -37.3)	0.45 (0.25, 0.64)	-0.63 (-0.8, -0.44)
Leeds	-41.2 (-45.9, -36.4)	2.6 (1.5, 3.7)	-43.7 (-48.5, -38.9)	0.34 (0.18, 0.48)	-1.18 (-1.38, -0.99)
Barcelona	-45.4 (-58.5, -31.0)	36.8 (29.5, 45.6)	-82.2 (-94.6, -69.8)	1.66 (1.3, 1.97)	-0.81 (-0.97, -0.63)
Edinburgh	-46.0 (-53.9, -37.7)	3.7 (2.6, 5.8)	-49.6 (-57.8, -42.1)	0.5 (0.32, 0.67)	-0.65 (-0.8, -0.5)
Lisbon	-51.7 (-58.4, -44.4)	13.1 (11.0, 15.5)	-64.8 (-71.4, -58.5)	0.6 (0.52, 0.66)	-0.58 (-0.7, -0.45)
Dublin	-57.9 (-62.7, -52.8)	1.8 (0.8, 3.6)	-59.7 (-64.8, -55.1)	0.28 (0.05, 0.47)	-0.58 (-0.62, -0.53)
Porto	-72.3 (-81.7, -62.6)	19.0 (15.9, 23.1)	-91.3 (-99.9, -83.3)	0.93 (0.74, 1.08)	-0.82 (-0.99, -0.63)
Glasgow	-89.4 (-102.9, -74.5)	6.1 (4.0, 9.0)	-95.6 (-109.2, -80.8)	0.78 (0.48, 1.03)	-1.27 (-1.59, -0.93)
London	-97.8 (-115.8, -77.0)	20.7 (14.5, 27.0)	-118.5 (-136.0, -98.7)	2.43 (1.82, 2.97)	-2.0 (-2.44, -1.57)

Supplementary References

1. Batista e Silva, F. *et al.* Uncovering temporal changes in Europe's population density patterns using a data fusion approach. *Nature Communications* **11,** 1–11. https://doi.org/10.1038/s41467-020-18344-5 (2020).
2. Jay, O. *et al.* Reducing the health effects of hot weather and heat extremes: from personal cooling strategies to green cities. *The Lancet* **398,** 709–724. doi:10.1016/S0140-6736(21)01209-5. http://www.thelancet.com/article/S0140673621012095/fulltext (2021).
3. Pace, M. *et al. Revision of the European Standard Population - Report of Eurostat's task force - 2013 edition* tech. rep. (Publications Office of the European Union, Luxembourg, 2013). doi:10.2785/11470.
4. Manoli, G., Fatichi, S., Bou-Zeid, E. & Katul, G. G. Seasonal hysteresis of surface urban heat islands. *Proceedings of the National Academy of Sciences of the United States of America* **117,** 7082–7089. doi:10.1073/PNAS.1917554117. https://www.pnas.org/doi/abs/10.1073/pnas.1917554117 (2020).
5. *English indices of deprivation 2019 - GOV.UK* Accessed: 2023-05-10. 2019. https://www.gov.uk/government/statistics/english-indices-of-deprivation-2019.
