# Supplementary material for: Economic valuation of temperature-related mortality attributed to urban heat islands in European cities
Source: Nat Commun. 2023 Nov 17;14:7438. doi: 10.1038/s41467-023-43135-z (PMC10656443; doi:10.1038/s41467-023-43135-z)
Supplement: Supplementary file 3 — Reporting Summary [file 41467_2023_43135_MOESM3_ESM.pdf]

Corresponding author(s): Wan Ting Katty Huang, Gabriele Manoli
Last updated by author(s): Oct 10, 2023

Reporting Summary

Nature Portfolio wishes to improve the reproducibility of the work that we publish. This form provides structure for consistency and transparency in reporting. For further information on Nature Portfolio policies, see our Editorial Policies and the Editorial Policy Checklist.

Statistics

For all statistical analyses, confirm that the following items are present in the figure legend, table legend, main text, or Methods section.

n/a	Confirmed	
☐	☒	The exact sample size (*n*) for each experimental group/condition, given as a discrete number and unit of measurement
☒	☐	A statement on whether measurements were taken from distinct samples or whether the same sample was measured repeatedly
☐	☒	The statistical test(s) used AND whether they are one- or two-sided<br>*Only common tests should be described solely by name; describe more complex techniques in the Methods section.*
☒	☐	A description of all covariates tested
☒	☐	A description of any assumptions or corrections, such as tests of normality and adjustment for multiple comparisons
☐	☒	A full description of the statistical parameters including central tendency (e.g. means) or other basic estimates (e.g. regression coefficient) AND variation (e.g. standard deviation) or associated estimates of uncertainty (e.g. confidence intervals)
☐	☒	For null hypothesis testing, the test statistic (e.g. *F*, *t*, *r*) with confidence intervals, effect sizes, degrees of freedom and *P* value noted<br>*Give P values as exact values whenever suitable.*
☒	☐	For Bayesian analysis, information on the choice of priors and Markov chain Monte Carlo settings
☒	☐	For hierarchical and complex designs, identification of the appropriate level for tests and full reporting of outcomes
☐	☒	Estimates of effect sizes (e.g. Cohen's *d*, Pearson's *r*), indicating how they were calculated

Our web collection on statistics for biologists contains articles on many of the points above.

Software and code

Policy information about availability of computer code

Data collection	No software was used for data collection
Data analysis	Mortality attribution analyses in R, version 4.0.5, and subsequent data analyses in Python, version 3.9, were performed through custom code. Though written by the author, these analysis codes do not contain novel methodology, and are available on GitHub at https://github.com/hkatty/Paper_UHI_mortality_Europe.

For manuscripts utilizing custom algorithms or software that are central to the research but not yet described in published literature, software must be made available to editors and reviewers. We strongly encourage code deposition in a community repository (e.g. GitHub). See the Nature Portfolio guidelines for submitting code & software for further information.

Data

Policy information about availability of data

All manuscripts must include a data availability statement. This statement should provide the following information, where applicable:

- Accession codes, unique identifiers, or web links for publicly available datasets
- A description of any restrictions on data availability
- For clinical datasets or third party data, please ensure that the statement adheres to our policy

Data generated from the current study, including attributed mortality, years of life lost, and associated economic assessments, have been deposited on Zenodo and can be accessed at https://doi.org/10.5281/zenodo.7986841.

Data used in the current study are described in detail in the Data section under Methodology above. UrbClim data can be obtained from Copernicus Climate Change Service (https://doi.org/10.24381/cds.c6459d3a), elevation map from MERIT DEM (http://hydro.iis.u-tokyo.ac.jp/~yamadai/MERIT_DEM), population density from NASA SEDAC (https://doi.org/10.7927/H49C6VHW), land imperviousness data from Copernicus Land Monitoring Service (https://land.copernicus.eu/pan-european/high-resolution-layers/imperviousness), Köppen-Geiger climate classification from https://www.gloh2o.org/koppen/, Eurostat data from https://ec.europa.eu/eurostat, data on mortality associated with PM2.5 from the supplementary materials associated with Khomenko et al. 2021 (https://doi.org/10.1016/S2542-5196(20)30272-2), data on mortality associated with ozone from the European Environment Agency (https://www.eea.europa.eu/data-and-maps/data/air-quality-health-risk-assessments, permalink: https://www.eea.europa.eu/ds_resolveuid/86ef37b3bf844299b978867c86cf99e7), and temperature-mortality relationships from https://doi.org/10.5281/zenodo.7672108.

Research involving human participants, their data, or biological material

Policy information about studies with human participants or human data. See also policy information about sex, gender (identity/presentation), and sexual orientation and race, ethnicity and racism.

Reporting on sex and gender	*Use the terms sex (biological attribute) and gender (shaped by social and cultural circumstances) carefully in order to avoid confusing both terms. Indicate if findings apply to only one sex or gender; describe whether sex and gender were considered in study design; whether sex and/or gender was determined based on self-reporting or assigned and methods used. Provide in the source data disaggregated sex and gender data, where this information has been collected, and if consent has been obtained for sharing of individual-level data; provide overall numbers in this Reporting Summary. Please state if this information has not been collected. Report sex- and gender-based analyses where performed, justify reasons for lack of sex- and gender-based analysis.*
Reporting on race, ethnicity, or other socially relevant groupings	*Please specify the socially constructed or socially relevant categorization variable(s) used in your manuscript and explain why they were used. Please note that such variables should not be used as proxies for other socially constructed/relevant variables (for example, race or ethnicity should not be used as a proxy for socioeconomic status). Provide clear definitions of the relevant terms used, how they were provided (by the participants/respondents, the researchers, or third parties), and the method(s) used to classify people into the different categories (e.g. self-report, census or administrative data, social media data, etc.) Please provide details about how you controlled for confounding variables in your analyses.*
Population characteristics	*Describe the covariate-relevant population characteristics of the human research participants (e.g. age, genotypic information, past and current diagnosis and treatment categories). If you filled out the behavioural & social sciences study design questions and have nothing to add here, write "See above."*
Recruitment	*Describe how participants were recruited. Outline any potential self-selection bias or other biases that may be present and how these are likely to impact results.*
Ethics oversight	*Identify the organization(s) that approved the study protocol.*

Note that full information on the approval of the study protocol must also be provided in the manuscript.

Field-specific reporting

Please select the one below that is the best fit for your research. If you are not sure, read the appropriate sections before making your selection.

☐ Life sciences ☐ Behavioural & social sciences ☒ Ecological, evolutionary & environmental sciences

For a reference copy of the document with all sections, see nature.com/documents/nr-reporting-summary-flat.pdf

Ecological, evolutionary & environmental sciences study design

All studies must disclose on these points even when the disclosure is negative.

Study description	The study involved analysis of temperature, land cover, and population data with established epidemiological exposure-response relationships, and subsequent data analyses to interpret the results.
Research sample	The study sample includes all adult populations in 85 European cities. The choice of cities was constrained by availability of both high resolution modelled temperature data from the Copernicus Climate Change Service (https://doi.org/10.24381/cds.c6459d3a) and modelled epidemiological exposure-response relationship for the city from Masselot et al. 2023 (https://doi.org/10.1016/S2542-5196(23)00023-2). The sample is meant to be representative of adult inhabitants in major cities across Europe.
Sampling strategy	As noted above, sampling was constrained by availability of data.
Data collection	Data were obtained by the first author through researching and downloading of published data in the literature and on data repositories, and in one instance, for the exposure-response relationships, the provision of data from a co-author, who have since published the data. A co-author who conceived the project was aware of the existence of the core datasets relevant for this study (that of temperature and the exposure-response relationships) prior to initiating the work, and subsequent data needed for the analysis were found through online search engines and core data repositories such as that for Eurostat.
Timing and spatial scale	Data were downloaded mainly between November 2021 and January 2022. The exposure-response relationships were updated during the revision stage in June 2023 to be consistent with the version published with the peer-reviewed paper. Other data were

	not resampled nor updated as they involved mostly static published data from prior years. Data were only obtained for European cities as this ensured the analysis was performed with a consistent dataset for all cities.
Data exclusions	Model grids containing water bodies or with elevations 100 m higher or lower than the city domain population density-weighted average elevation are excluded. The former is excluded as residential population on water bodies is minimal. The latter is excluded due to elevation's impact on temperature independently of surface properties.
Reproducibility	The experiment (i.e. data analysis) was performed for the entirety of the most suitable data currently available, thus no attempts were made to repeat the experiment. For future reproducibility checks, a different set of data providing the same or similar measures would be required.
Randomization	In one part of the analysis, cities were grouped into Köppen-Geiger climate groups, which is a widely used classification of climate types. The purpose of the grouping was only to provide an additional layer of interpretation. It did not affect the findings themselves.
Blinding	Blinding was not relevant in this study, as analyses were performed for all data (all cities and all age groups) equally through computer code. Characteristics of each city and age group only became relevant in the interpretation stage to speculate on possible reasons behind the findings.

Did the study involve field work? ☐ Yes ☒ No

Reporting for specific materials, systems and methods

We require information from authors about some types of materials, experimental systems and methods used in many studies. Here, indicate whether each material, system or method listed is relevant to your study. If you are not sure if a list item applies to your research, read the appropriate section before selecting a response.

Materials & experimental systems

n/a	Involved in the study
☒	☐ Antibodies
☒	☐ Eukaryotic cell lines
☒	☐ Palaeontology and archaeology
☒	☐ Animals and other organisms
☒	☐ Clinical data
☒	☐ Dual use research of concern
☒	☐ Plants

Methods

n/a	Involved in the study
☒	☐ ChIP-seq
☒	☐ Flow cytometry
☒	☐ MRI-based neuroimaging
